# APOBEC3C coordinates DDX5 in R-loop resolution and dynamic control of Chk1-mediated stress-responsive circuitry as a prerequisite for gemcitabine resistance in p53-deficient cells

**DOI:** 10.1038/s41419-025-08215-6

**Published:** 2026-01-07

**Authors:** Li Tao, Yang Zhao, Zhuangzhaung Jiang, Shujing Kong, Yanlin Ding, Tengyang Ni, Weimin Wang, Yanqing Liu

**Affiliations:** 1 Department of Pharmacy, College of Medicine, Yangzhou University, Yangzhou, Jiangsu China; 2 The Key Laboratory of Syndrome Differentiation and Treatment of Gastric Cancer of the State Administration of Traditional Chinese Medicine, Yangzhou University, Yangzhou, Jiangsu China; 3 Department of Medicine, Linfen Vocational and Technical College, Linfen, Shanxi China; 4 School of Traditional Chinese Pharmacy, China Pharmaceutical University, Nanjing, Jiangsu China; 5 Department of Biochemistry and Structural Biology, University of Texas Health Science Center at San Antonio, San Antonio, TX USA; 6 Department of Oncology, Yixing Hospital Affiliated to Medical College of Yangzhou University, Yixing, Jiangsu China

**Keywords:** Cancer therapeutic resistance, Checkpoint signalling

## Abstract

Genomic instability is a hallmark of cancer, encompassing both sequence and structural alterations that drive tumor evolution and heterogeneity. The APOBEC3 family of deoxycytidine deaminases has emerged as a major source of mutagenic activity in cancers. R-loops are RNA-DNA hybrids and structural barriers that interfere with replication and transcription. Among the APOBEC3 family, APOBEC3C (A3C) is particularly worthy of attention for its upregulation, driving the DNA replication stress tolerance in response to replication stress-inducing drug gemcitabine. However, the molecular mechanisms of gemcitabine resistance and regulatory circuitries mediated by A3C remain largely unknown, especially in checkpoint-deficient tumors. Initially, we screened that A3C was a putative transcriptional target of p53, and p53-deficient H1299 cells harboring A3C elicited a chemoresistant phenotype upon gemcitabine treatment both in vitro and in vivo. A3C expression enhanced Chk1-dependent S-phase checkpoint activation, thus slowing down replication fork progression and facilitating DNA repair. Pull-down assay and proteomic analysis identified that A3C had a specific interaction with the RNA helicase DDX5, which coordinately played critical roles in R-loop resolution. In contrast to A3C, DDX5 expression attenuated Chk1-dependent S-phase checkpoint activation. Knockdown of DDX5 in A3C-proficient H1299 cells attenuated gemcitabine-induced Chk1 activation and enhanced the therapeutic index of gemcitabine by promoting R-loop accumulation. Therefore, we conclude that A3C/DDX5/R-loop complex may impair the sensitivity of gemcitabine by modulating Chk1 dynamics and DNA replication/damage response machinery.

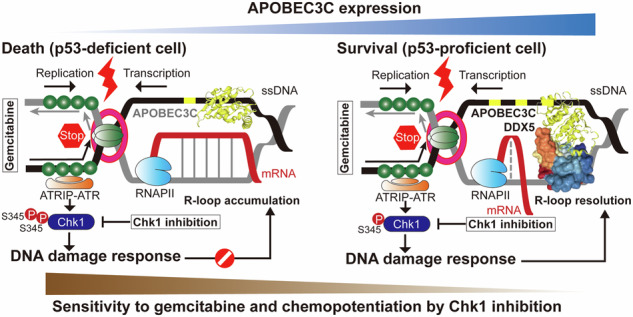

## Introduction

The apolipoprotein B mRNA editing enzyme catalytic polypeptide-like 3 (APOBEC3 or A3) family of cytidine deaminases has emerged as a major source of genomic instability in cancer by operating on single-stranded (ss) DNA and driving the C-to-U/T mutation [1, 2]. This family comprises seven members (A3A, A3B, A3C, A3D, A3F, A3G and A3H), encoded by a tandemly arranged gene cluster on chromosome 22. Importantly, A3 has not only been implicated in tumor evolution but also associated with therapeutic resistance. Anticancer therapies, including DNA-damaging agents and targeted inhibitors, can induce the expression and activity of A3 enzymes in tumors post-treatment [3]. Most previous studies have primarily focused on A3A and A3B due to their well-established roles in promoting mutagenesis and cancer progression [4]. A3C is a single zinc-dependent cytidine deaminase that differs from its paralogs A3A and A3B in both structure and activity [5]. Growing evidence indicates that A3C is associated with a characteristic mutational signature in pancreatic cancer [6], and may also contribute to resistance against gemcitabine, an antimetabolite widely used in chemotherapy. A3C has been shown to facilitate gemcitabine resistance by promoting the resolution of stalled replication forks, thereby enabling cells to survive genotoxic stress [7]. However, the key determinants through which A3C modulates cellular responses to gemcitabine beyond replication fork stabilization remain poorly understood. A deeper investigation into the broader role of A3C in gemcitabine resistance may reveal novel therapeutic targets to overcome drug resistance.

In addition to sequence instability induced by A3 enzymes, DNA structural instability caused by R-loop also plays a critical role in genomic stability and therapeutic response. R-loops are three-stranded, co-transcriptional nucleic acid structures when the nascent RNA hybridizes with the template DNA strand. To timely remove harmful R-loops and preserve genomic integrity, several RNA helicases belonging to the DExD/H-box (DDX) family have been reported to unwind the RNA: DNA hybrids to facilitate R-loop disassembly. In particular, the role of DDX5 in R-loop resolution has been formally established [8–11]. Moreover, DDX5 is essential for homologous recombination repair at R-loop sites while BRCA2/RAD51 supports DDX5 to manipulate double-strand breaks (DSBs) [9, 12]. Noticeably, the non-template DNA in R-loop structure is exposed as ssDNA, which can be easily attacked by DNA cytidine deaminases that generate DNA breaks and the replication fork stalls at the R-loop lesions [13]. Importantly, a recent study demonstrated that A3B directly binds to R-loop structures and promotes their clearance, thereby reshaping the genomic R-loop landscape and contributing to transcription-associated mutagenesis in cancer cells [14]. Hence, the cellular crosstalk of deaminases with helicases at R-loop sites raises questions as to how they are also involved and regulated in R-loop biology that anchors the DNA damage response (DDR) machinery and therapeutic response to chemotherapy.

As one of the most frequently mutated tumor suppressors in human cancers, loss of p53 function impairs the canonical DNA damage checkpoint, rendering tumor cells unable to effectively arrest the cell cycle or undergo apoptosis in response to genotoxic stress. As a compensatory mechanism, p53-deficient cells often become highly reliant on the ATR/Chk1 signaling to survive replication-associated stress and maintain genome integrity. Since persistent R-loop formation can be a major source of structural barriers to DNA replication and transcription. ATR/Chk1 has been reported to be a primary sensor to safeguard the genome against R-loop [15–17]. In this context, the ability to resolve or tolerate R-loops becomes heavily dependent on Chk1 signaling in p53-deficient cells. In the present study, we aim to uncover mechanistic link between A3 proteins, R-loop hemostasis, checkpoint signaling and cancer therapy. Our data demonstrate that A3C interacts with the RNA helicase DDX5 and regulated R-loop homeostasis by dynamically modulating Chk1 phosphorylation under gemcitabine-induced replication stress, which facilitates gemcitabine tolerance in p53-defective lung cancer cells. These findings suggest that A3C/DDX5/R-loop axis might clue for the efficacy of chemotherapy and combination therapies associated with DNA damage response pathways especially in tumors lacking functional p53.

## Materials and methods

### Cell lines and cell cultures

The H1299 and A549 non-small-cell lung cancer (NSCLC) cell lines were maintained in RPMI-1640 containing 10% fetal bovine serum (FBS). HEK-293T cells were cultured in DMEM medium supplemented with 10% FBS. The DNA (STR) profiles of these cells within 6 months of use were authenticated by GENEWIZ, Inc (Suzhou, China) using GenePrint® 10 System (Promega, cat. no. B9051). Cultures were negative for mycoplasma after testing with EZ-PCR mycoplasma test kit (Biological Industries, cat. no. 20-700-20).

### Chemicals and reagents

Gemcitabine hydrochloride ≥98% (HPLC purity) was purchased from Sigma-Aldrich (cat. no. G6423). A 50 mM stock solution was prepared in ddH_2_O, and cells that were treated with an equal volume of ddH_2_O served as controls. Chk1 inhibitors including MK-8776 (cat. no. S2735) and AZD-7762 (cat. no. S1532) were purchased from Selleck Chemicals. A 50 mM stock solution was prepared in 100% DMSO, and cells that were treated with an equal volume of DMSO served as controls.

### Patient samples and immunochemistry

Formalin-fixed paraffin-embedded (FFPE) primary lung adenocarcinoma specimens from three independent patients were obtained from the Biobank of Yixing People’s Hospital Affiliated to Yangzhou University. Patients provided informed consent in accordance with an established protocol approved by the Ethics Committee of Yixing People’s Hospital, Affiliated to Yangzhou University (approval no. 2025-063-01), which was performed in accordance with the principles of the Declaration of Helsinki. To assess A3C expression in patient tissues, sections were dewaxed, rehydrated, and underwent antigen retrieval. Subsequently, the tissue slides were blocked with 5% normal goat serum and incubated with a 1:100 dilution of anti-APOBEC3C (Proteintech, cat. no. 10591-1-AP) followed by incubation with the UltraSensitive^TM^ SP (Rabbit) IHC Kit (MaiXin Biotechnology, cat. no. KIT-9710). Images were captured using the Leica inverted microscope (DMi8) under a 20× lens.

### Gene overexpression or knockdown

The open reading frame of human *TP53* sequence-verified cDNA (clone ID: 3544714, purchased from Dharmacon) was subcloned to pcDNA3.1(+) by BamHI (up) and NotI (down). For the generation of the hot spot mutations (R175H, R248W and R273H) of p53, the appropriate residue of the wild-type p53 was mutated via overlapping polymerase chain reactions using the specific mutation-containing primers and the QuikChange II XL site-directed mutagenesis kit (Agilent, cat. no. 200522). All the mutants were generated and fully sequenced by GENEWIZ, Inc. CDA (RNA accession: NM_001785, cat. no. RC208922), A3C (RNA accession: NM_014508, cat. no. RC203971), human Myc-DDK-tagged ORF clone and corresponding pCMV6-Entry (cat. no. PS100001) vectors were purchased from OriGene Technologies, Inc. C97S/C100S mutants of A3C were generated via overlapping polymerase chain reactions (PCRs) using the specific mutation-containing primers and fully sequenced by GENEWIZ. HA-tagged DDX5 (RNA accession: NM_004396.5) expression vector was purchased from GeneCopoeia (cat. no. EX-F0232-M07). ppyCAG_RNaseH1_WT (plasmid no. 111906) and ppyCAG_RNaseH1_D210N (plasmid no. 111904) were ordered from Addgene. Transfections were performed using opti-MEM medium (Gibco, cat. no. 31985070) plus X-tremeGENE HP DNA transfection reagent (Roche, cat. no. 06366236001), according to the manufacturer’s directions. Stably transfected cells expressing the target gene were obtained by selection for neomycin resistance using 1 mg/mL G418 (Gibco, cat. no. 10131027) for 5 days and single colonies were harvested by serial dilution in 96-well plates.

For small interfering RNA (siRNA)-mediated gene knockdown, AccuTarget™ predesigned nonspecific scrambled control, p53 siRNA (accession: NM_000546.5, siRNA ID: 7157) were purchased from Bioneer. Transfection of individual siRNA and control siRNA (100 pmol for each siRNA) was performed using Lipofectamine RNAiMAX (Thermo Fisher Scientific, cat. no. 13778150) according to the manufacturer’s protocol. For small hairpin RNAs (shRNA)-mediated gene knockdown, lentiviral particles carrying A3C (cat. no. HSH173688, accession: NM_014508.3) or DDX5 (cat. no. HSH152471, accession: NM_004396.5)-targeting shRNA sequence and scrambled control were ordered from GeneCopoeia. Cells were transduced with 5 MOI of single shRNA viral particles or pooled viral cocktail plus 8 μg/mL Polybrene for 24 h. For stable knockdown, cells were selected with 2 μg/mL puromycin in the first 3 days and resistant clones were routinely maintained with 0.5 μg/mL puromycin.

### Real-time PCR

RNA was extracted from cells using RNeasy Mini Kit (Qiagen, cat. no. 74104) followed by cDNA synthesis using SuperScript™ III Reverse Transcriptase (Invitrogen, cat. no. 18080093). Quantitative PCR analysis was performed on a 7900HT Fast Real-Time PCR System using Fast SYBR® Green Master Mix protocol (Applied Biosystems, cat. no. 4385612). The primer sequences (Supplementary Table. 1) were verified with almost equivalent amplification efficiencies and specific to measure the mRNA levels of each APOBEC3 gene [18].

### Cell viability and drug interaction analysis

Cell viability was determined by MTS assay. Briefly, cells were seeded in 96-well cell culture plates and treated with drugs as indicated. After incubation for the indicated time period, 20 μL of the One Solution reagent (Promega, cat. no. G3582) was added to each well and incubation was continued for additional 1–4 h. Absorbance was measured at 490 nm using Enspire Multilabel Plate Reader (PerkinElmer). The effect of indicated agents on cell viability was assessed as relative cell viability (%) compared with vehicle-treated control cells.

The drug combination effect on cell viability was evaluated by established mathematical models. The Combenefit software utilizes the Loewe additivity, Bliss independence and highest single agent (HSA) models to quantify drug interaction. A dose-response matrix (experimental data) is generated according to the combination of dose-response curves of each drug combination, and the program will calculate the degree of combined effect in a heat map, where blue color signifies a synergistic effect and yellow color signifies an antagonistic effect [19]. The CompuSyn software applies the combination index (CI) to express the properties of drug interaction, which is based on the median-effect principle developed by Chou and Talalay [20]. CI values below 1 indicate a synergistic effect of the drug combination, while CI values ranging from 0.1 to 0.3 usually signify strong synergism.

### Colony formation assay

Briefly, H1299 cells stably transfected with A3C or empty vector counterpart were inoculated into 6-well culture plates in triplicates (500 cells/ well) and allowed to attach for 24 h. Cells were challenged with low concentrations of gemcitabine in the presence or absence of Chk1 inhibitors. At the end of culture for 2 weeks, cells were fixed and stained with crystalline violet solution. Colonies were visualized and counted using ImageJ software. Colony formation efficiency was measured by colony size, which took the intensity (colony surface area) into account using the ColonyArea ImageJ plugin. All the data were normalized to vehicle controls and expressed as fold matched control (% of colony formation).

### EdU incorporation assay

To assess DNA replication, H1299 cells stably transfected with A3C or an empty vector counterpart were inoculated into 60-mm Petri dishes in triplicate and treated with gemcitabine in the presence or absence of Chk1 inhibitors. After 24 h incubation time, cells were pulsed with 10 μM EdU (5-ethynyl-2’-deoxyuridine) for 2 h and detected using a Click-iT™ Plus EdU Alexa Fluor 647 Flow Cytometry Kit (Molecular Probes, cat. no. C10634). Cytofluorometric acquisitions were performed on LSRFortessa cytofluorometer (Becton Dickinson).

### Alkaline comet assay

H1299 cells stably transfected with A3C or empty vector counterpart were inoculated into 60-mm Petri dishes in triplicate and treated with gemcitabine in the presence or absence of Chk1 inhibitors. After 48 h, cells were harvested and detected for DNA strand breaks using a comet assay kit according to the manufacturer’s instructions (Abcam, cat. no. ab238544). Cell suspension at a density of 2 × 10^5^ cells/mL was mixed with comet agarose and then added onto comet slide in triplicate for each group. After gelling in a refrigerator, the slide was immersed in cold lysis buffer followed by electrophoresis buffer for DNA electrophoresis. After neutralization and dehydration, slide was stained with the green DNA stain. The slide was imaged using a fluorescence microscope. A total of 50 cells were randomly counted from each group. The ImageJ software was used to quantify tail moment, which was calculated by the grey values of a comet tail/grey values of whole region × tail length × 100%.

### Metaphase spreading assay

H1299 cells stably transfected with A3C or empty vector counterpart were inoculated into 60-mm Petri dishes in triplicates and treated with gemcitabine in the presence or absence of Chk1 inhibitors. After 48 h, cells were further treated with 0.2 μg/mL colcemid (Biological Industries, cat. no. 12-004-1D) for 4 h. Cells were trypsinized and treated in a dropwise manner with prewarmed 0.075 M KCl. After 30 min incubation at 37 °C, cells were fixed in freshly prepared methanol∶ acetic acid mix (3:1), and dropped onto coverslips and stained with Giemsa solution. Chromosome morphology was examined under microscopy with 100× magnification. Chromosome morphology evaluation was performed with at least 20 nuclei, and the percentage of cells with aberrant chromosomes adjacent to the nuclei was analyzed.

### Western blot analysis

Whole-cell lysates were prepared with RIPA buffer containing protease inhibitor cocktail (Roche, cat. no. 11873580001) and 1% phosphatase inhibitor cocktail #2 and #3 (Sigma, cat. no. P0044 and P5726). Equal amounts of cell lysates (25 μg) were resolved by 10% SDS-PAGE and transferred onto PVDF membranes. Membranes were incubated with monoclonal antibodies against: p53-DO-1 (Abcam, cat. no. ab1101), APOBEC3C (Proteintech, cat. no. 10591-1-AP), phospho-Chk1 Ser345 (CST, cat. no. 2348), Chk1 (CST, cat. no. 2360), phospho-RPA32/RPA2 Ser8 (CST, cat. no. 54762), RPA32/RPA2 (CST, cat. no. 35869), phospho-H2AX Ser139 or γH2AX (Sigma-Aldrich, Cat. no. 05-636) at dilution of 1∶1000, GAPDH (Abcam, cat. no. ab8245) at dilution of 1∶10,000, and FLAG (Sigma, cat. no. F3165) at a dilution of 1∶5000. Membranes were incubated with HRP-conjugated anti-rabbit or anti-mouse IgGs (Proteintech, cat. no.SA00001-2 and SA00001-1) at a dilution of 1∶10,000 followed by enhanced chemiluminescence (Millipore, cat. no. WBKLS0500) and visualized with a ChemiDoc XRS system (Bio-Rad).

### Immunoprecipitation and proteomic analysis

H1299 cells were transiently expressed with FLAG-tagged A3C. After 48 h transfection, cells were lysed with IP Lysis Buffer containing 25 mM Tris (pH 7.5), 150 mM sodium chloride, 1% NP-40, 1 mM EDTA, 5% Glycerol, complete protease inhibitor cocktail. Cell lysates were incubated on ice for 30 min and lysates were clarified by centrifugation at 12,000 *g* for 15 min at 4 °C. Protein concentration was quantified using BCA assay and 0.5 mg clarified lysates were incubated with 40 μl anti-FLAG M2 affinity gel (Sigma, cat. no. A2220) on a rotating wheel at 4 °C overnight. The beads were subsequently washed thrice with ice-cold RIPA lysis buffer, and subsequently the bound proteins were eluted by boiling the beads at 95 °C for 15 min with 50 μl 4× NuPAGE LDS sample buffer supplemented with 1 mM DTT. Protein complexes were purified on an SDS-polyacrylamide gel, followed by tryptic digestion and desalting using trifluoroacetic acid on a C18 spin column plate. Peptides were separated and analyzed using a liquid chromatography-tandem mass spectrometry (UltiMate 3000 RSLCnano system coupled with Q Exactive HF-X mass spectrometer, Thermo Fisher Scientific). Peptide identification was searched with Proteome Discoverer 1.4 software (Thermo Scientific) against Uniprot human complete proteome database (Proteome ID: UP000005640). To find functional protein network that interact with A3C, bioinformatics analysis of all the protein data was performed using the online tool Metascape. The Molecular Complex Detection (MCODE) algorithm was applied to identify densely connected network components. All proteins were listed in Supplementary Table 2. To validate the interaction between A3C and DDX5, the immunoprecipitated proteins were separated by SDS-PAGE and Western blot was performed with the indicated antibodies.

### Quantitation of gemcitabine in cellular extracts

Equal numbers of H1299 cells, which were stably transfected with CDA, A3C or empty vectors, were plated into 35 mm Petri dishes prior to treatment. For time course analysis, 20 μg/mL of gemcitabine was added to each well for 15, 30, 60, 90 and 120 min after incubation. For dose course analysis, 1.25, 2.5, 5, 10 and 20 μg/mL of gemcitabine was added to each well for 120 min incubation. After treatment, all the dishes were then washed with PBS thrice. The cell samples were lysed in 100 μL deionized water and snap frozen in liquid nitrogen. To get homogenized cell lysates, cells were thawed at 37 °C and subjected to ultrasonication. Blank cell lysates were prepared in H1299 cells for the calibration standards and the quality control (QC) samples. For protein precipitation, samples were mixed with ice-cold methanol in a 1∶9 ratio (v/v), vortexed, and incubated at –20 °C overnight. The mixtures were centrifuged at 12,000 rpm for 30 min to pellet proteins. High-throughput ultra-high performance liquid chromatography-tandem mass spectrometry (UHPLC-MS/MS) was performed to detect the intracellular gemcitabine using a Thermo Scientific™ Vanquish™ UHPLC system with a TSQ Altis triple-quad mass spectrometer, a binary pump, split loop autosampler, column compartment and both diode array and charged aerosol detectors. The internal standard (IS) floxuridine was obtained from National Institute for the Control of Pharmaceutical and Biological Products (cat. no. Z140660). Floxuridine was added to each sample at a final concentration of 500 ng/ml. LC method: mobile phases solvent A contained 0.5% formic acid mobile phase B contained 0.1% formic acid and acetonitrile: 0.5%; column: Waters Cortecs UHPLC HILIC 2.7 μm 4.6 × 75 mm, flow rate: 0.5 mL/min. Retention time of floxuridine and gemcitabine: 1.59, 1.67 and 2.67 min. The lower limit of quantification (LLOQ) of the UHPLC-UV assay was 1 ng/mL for gemcitabine. Mass spectrometry scan method: all the analytes were ionised by electrospray ionisation (ESI). Pressure for positive and negative ion modes was 3800 and 2500V, respectively. Ion source temperature: 350 °C. The analysis was performed in three biologically independent experiments.

### Bioinformatic analysis

A comparison of gene expression patterns of the APOBEC3 family between tumor and normal tissues in lung adenocarcinoma was performed using the web tool TNMplot, in which the molecular profiles of cancerous and adjacent tissues were obtained from the Cancer Genome Atlas (TCGA), while the molecular profiles of healthy samples were from the Genotype-Tissue Expression (GTEx) project [21]. To identify potential essential genes of APOBEC3 family that are critical for cell survival, the Cancer Dependency Map (DepMap: https://depmap.org/portal/) was used to screen highly dependent members in cancer cell lines, which is measured by gene dependency score. A highly negative dependency score can be classified as gene addiction in a particular cancer cell line. The gene expression patterns of APOBEC3 family in human cancer cell lines and *TP53* gene mutations were retrieved from DepMap. We also carried out a comparison of gene expression patterns of APOBEC3 family between wild-type p53 cell lines with mutant p53 cell lines. The protein levels of selected genes in lung adenocarcinoma were obtained from Clinical Proteomic Tumor Analysis Consortium (CPTAC) [22]. The effects of target gene expression on overall survival (OS), progression-free survival (PFS) in lung adenocarcinoma patients were investigated by Kaplan-Meier Plotter [23]. Based on gene correlations by co-expression pattern, we employed Gene Set Enrichment Analysis (GSEA) to find significant pathways from curated gene sets from the Molecular Signatures Database (MSigDB) using the web tool correlationAnalyzeR [24]. To analyze the correlation between gemcitabine sensitivity and A3C expression, the GDSC2 drug sensitivity data from the Genomics of Drug Sensitivity in Cancer (GDSC) database were used as the training set. The oncoPredict package [25] and R Studio (version: R4.3.1) were employed to predict gemcitabine sensitivity towards cell lines with high and low levels of A3C.

### GFP reporter assay

The potential role of A3C in regulating the activity of double-strand DNA break repair by either homologous recombination (HR) or non-homologous end joining (NHEJ) was determined by a GFP-reporter assay. In brief, H1299 cells stably expressing A3C were transfected with siRNA targeting A3C or scrambled-negative control in a 6-well plate, followed by co-transfection with GFP-reporter plasmids (1 μg DNA/well for each plasmid). Cells without pCBASceI transfection served as negative controls. The pDRGFP (plasmid no. 26475), pimEJ5FP (plasmid no. 44026) and pCBSceI (plasmid no. 26477) were ordered from Addgene. HEK-293T cells were used to normalize for transfection efficiency. After 48 h, cells were harvested and GFP-positive cells were quantified by LSRFortessa cytofluorometer (Becton Dickinson).

### DNA fiber spreading assay

The potential role of A3C in regulating DNA replication was determined by a DNA fiber spreading assay. In brief, H1299 cells stably expressing A3C were transfected with siRNA targeting A3C or scrambled negative control in a 6-well plate. Cycling cells were sequentially labeled with 25 μM 5-Chloro-2’-deoxyuridine (CldU, Sigma-Aldrich, cat. no. C6891) for 30 min followed by 250 μM 5-Iodo-2’-deoxyuridine (IdU, Sigma-Aldrich, cat. no. I7125) for another 30 min. After each pulse labeling period, cells are washed with ice cold PBS. Cultures were routinely harvested and resuspended in 100 μL PBS. A total of 2 μL of cell suspension were spotted on a positively charged glass slide (Epredia™ SuperFrost Plus Adhesion slides) and lysed with 10 μL of fiber spreading buffer (200 mM Tris-HCl pH 7.4, 50 mM EDTA, 0.5% SDS). After incubation, the slides were tilted at a 15° angle, allowing the cells to spread along the slide at a constant speed. Stretched DNA fibers were fixed in methanol/acetic acid (3∶1) solution for 10 min, air-dried and denatured with 2.5 M HCl for 1 h. Slides were washed with PBS, blocked with 5% BSA in PBS for 2 h. Immunostaining assay was performed with rat anti-BrdU (1∶100, Abcam, cat. no. ab6326) for CldU and mouse anti-BrdU antibody (1∶40, Becton Dickson, cat. no. 347580) for IdU overnight at 4 °C in a humid chamber. Slides were then washed with 0.1% Tween- 20 in PBS for 5 min. The following secondary antibodies were used: anti-rat AlexaFluor 594 (1∶100, Invitrogen, cat. no. A11007) and anti-mouse AlexaFluor 488 (1∶100, Invitrogen, cat. no. A11001). After washing, the slides were air-dried and mounted on coverslips with antifade mounting medium. Fibers were visualized using Olympus Fluorescence Microscope (Upright BX61) under 100× oil immersion lens. A minimum of 150 tracts were recorded per group and fork speed in kb/min was calculated by dividing the average length of the red plus green tract by the pulse time. The DNA tract lengths were measured using ImageJ and micrometer values were converted into kilobase using the conversion factor 1 μm = 2.59 kb.

### In silico prediction of protein–protein interfaces

The artificial intelligence-based complex prediction of A3C/DDX5 was performed using AlphaFold-Multimer v3 with the online implementation of ColabFold. The PDB100 structural database was used as the template library to assist structure alignment. For the predictions, full-length A3C (residues 1-190), or the deaminase domain of A3C (residues 29-138) along with full-length DDX5 (residues 1-614), N-terminal DDX5 (residues 40-122), or C-terminal DDX5 (residues 477-614) were used. Model quality was assessed through key AlphaFold metrics, such as interface predicted TM-score (ipTM) and predicted TM-score (pTM) with predicted alignment error (PAE) matrices, which allowed detailed examination of the structural confidence across the predicted interface. For structural visualization, the crystal structure of A3C (3VOW) and DDX5 (3FE2) was retrieved from the RCSB protein data bank. The two proteins were further processed in PyMOL by removing water molecules, ligands, and ions, leaving only the pure protein chains. (3vow_clean.pdb for A3C and 3fe2_clean.pdb for DDX5). The protein-protein docking procedure was performed by ZDOCK (https://zdock.wenglab.org/), a widely used and highly reliable rigid-body docking program. The docking results were analyzed using the PDBePISA (https://www.ebi.ac.uk/pdbe/pisa/) to assess the interface area, interface energy, and potential hydrogen bonds and non-covalent interactions. PyMOL was then used to visualize the docking results, focusing on the spatial arrangement of protein-protein interactions and key binding sites. The hot-spot prediction was performed by mutation of a tyrosine residue to alanine using PyMOL program.

### Tumor xenograft assay

Animal research and corresponding animal utilization protocol (AUP) for this study were approved by the Animal Care and Use Committee of College of Medicine, Yangzhou University (approval no. YXYLL-2022-117). Studies with animals are described in compliance with the ARRIVE guidelines for reporting experiments involving animals. To establish tumor xenograft, indicated cells were mixed at a 2∶1 ratio with Matrigel and a 200 μL suspension containing 5 × 10^6^ cells was subcutaneously inoculated into the right flank of each mouse. Power analyses indicate that sample sizes of at least 6 mice per group are required for a β ≤ 0.2 and α ≤ 5%. Tumor growth was regularly monitored at every day by caliper, and the volume was calculated according to the formula: volume = width^2^ × length × 0.5. Tumor- bearing mice were randomly assigned to control and experimental groups when tumors reached a median size of 100 mm^3^. Once the maximum tumor volume reached 2000 mm^3^, mice were then sacrificed.

### R-loop analysis by dot-blot assay

The dot-blot assay was performed based on the protocol with minor modifications [26]. In brief, genomic DNA was extracted from cells using a standard phenol-chloroform protocol. All reagents and chemicals purchased commercially are guaranteed to be RNase-free. DNA concentration was measured using a NanoDrop spectrophotometer. Each DNA sample (200 ng) were spotted onto an Amersham™ Hybond™-N^+^ membrane (Cytiva, cat.no. RPN303B,), UV-crosslinked, and blocked in 5% BSA in DEPC-treated TBST for 1 h at room temperature. R-loops were detected by incubating the membrane overnight at 4 °C with the S9.6 monoclonal antibody (Kerafast, cat. no. ENH001, 1:1000). After washing, membranes were incubated with HRP-conjugated anti-Mouse IgGs (1:10,000) for 2 h at room temperature, followed by detection using enhanced chemiluminescence. RNase H (NEB, cat. no. M0297S) was used to confirm the specificity of the S9.6 signal. Loading control is obtained by staining the membrane with methylene blue (MB).

### Immunofluorescence assay

In brief, cells were seeded at 1 × 10^5^ per well in a 6-well plate containing a glass coverslip in each well. After treatment, cells were fixed in precooled acetone for 15 min and rinsed three times with PBS. Cells were then permeabilized in 0.1% Triton X-100 and incubated with 1% BSA/PBS to block nonspecific antibody binding for 30 min at room temperature. The anti-DNA/RNA Hybrid [S9.6] antibody antibodies (Kerafast, cat. no. ENH001, 1∶100) or 53BP1 (Merck, cat.no. MB3802, 1∶100) were used for immunostaining. Nuclei were counterstained with DAPI. The coverslips were mounted onto glass slides with ProLong® Gold Antifade Reagent (Molecular Probes, cat. no. P36930). For quantitative analysis, foci were counted per cell using a two-photon laser confocal microscope with 63× magnification objective lens (Zeiss LSM 880 NLO). Nuclei foci count was performed with at least 40 cells under blinded conditions.

For tumor xenografts, frozen tissue sections were fixed with cold acetone for 10 min and were blocked with 5% BSA containing 0.3% TritonX-100 for 2 h. Sections were incubated with a 1:100 dilution of S9.6 antibody (Kerafast, cat. no. ENH001) and DDX5 (CST, cat. no. 9877), followed by incubation with a 1∶200 dilution of Alexa Fluor 647 or 488-conjugated goat anti-mouse/rabbit secondary antibody and ProLong Gold Antifade Mountant with DAPI, and then observed under a confocal microscope with 40× magnification objective lens (Zeiss LSM 880 NLO).

### Statistical analysis

Data are shown as averages with standard deviations. Statistical comparisons between two groups were evaluated using either two-tailed Student’s *t* test or non-parametric Mann-Whitney *U* test. One-way ANOVA followed by Dunnett’s or Turkey’s post hoc test, or non-parametric Kruskal-Wallis test was applied for comparisons among multiple groups. Differences where *P* < 0.05 (*), *P* < 0.05 (**), *P* < 0.001 (***) were considered statistically significant. Statistical parameters, including sample size and exact statistical significance, are reported in the figures or legends. Data were analyzed and plotted using GraphPad Prism 9.

## Results

### Aberrant A3C expression was associated with p53 mutation and gemcitabine sensitivity

**Fig. 1:**
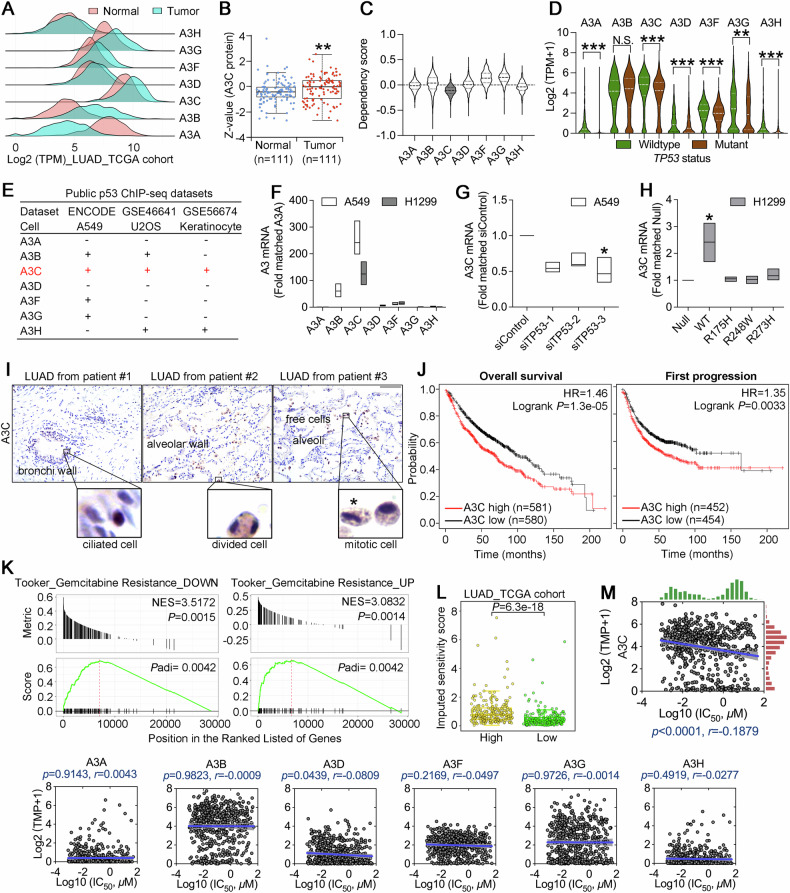
Clinical significance of A3C in lung adenocarcinoma. **A** Transcriptional expression pattern of seven APOBEC3 family members in LUAD (green) and normal lung tissues (red), which was visualized using ridgeline plot generated by TNMplot. **B** Protein expression levels of A3C in LUAD or normal lung tissue based on the CPTAC proteomics datasets. The Mann-Whitney *U* test was used to compare the mean of two groups (**P < 0.01). **C** Genetic dependency scores of seven APOBEC3 family members across hundreds to thousands of cancer cell lines using genome-wide CRISPR-Cas9 screening data in the Broad DepMap database. A dependency score < 0 indicates that loss of the gene impairs cell viability, with more negative scores reflecting higher survival essentiality. **D** Differential expression of seven APOBEC3 family members according to *TP53* mutation status in cancer cell lines (wildtype, n = 536; mutant, n = 910) in the Broad DepMap database. The Mann-Whitney *U* test was used to compare the mean of two groups. ***P < 0.001 for A3A, A3C, A3D, A3F and A3H, **P = 0.0054 for A3G, *P* = 0.1740 for A3B (N.S. not significant). **E** ChIP-seq analysis from multiple datasets (GSE46641, GSE56674, ENCODE) revealed p53 binding enrichment at seven APOBEC3 family genes across various cell types and stress conditions. **F** Relative mRNA expression levels of the seven APOBEC3 family members in A549 (TP53 wild-type) and H1299 (TP53-null) cells using quantitative RT-PCR (normalized to GAPDH, fold matched A3A, representative of n = 3). **G** Relative mRNA expression levels of A3C in A549 cells transfected p53-targeting siRNAs (normalized to GAPDH, fold matched siControl, representative of n = 3, *P = 0.0364). **H** Relative mRNA expression levels of A3C in H1299 cells transfected with empty vector (pcDNA3.1), wild-type or mutant p53 (R175H, R248W, R273H) expression vectors (normalized to GAPDH, fold matched null control, representative of n = 3, *P = 0.0240). The non-parametric Kruskal-Wallis test was used to compare differences among groups for quantitative RT-PCR analysis. **I** Immunohistochemical staining of A3C in three NSCLC patient samples. A3C expression was detected in bronchial epithelium (Patient #1), alveolar walls including a mitotic cell (Patient #2), and scattered atypical cells resembling tumor cells in the interstitium (Patient #3), with nuclear and cytoplasmic localization. **J** Patients were stratified into high and low A3C expression groups using the median expression value as the cutoff. Survival analysis, including overall survival (OS) and first progression (FP) was performed by Kaplan–Meier plotter website. **K** Genes were ranked by their correlation with A3C expression in LUAD and enriched pathways were identified using corGSEA tool offered by Correlation AnalyzeR website. **L** Predicted gemcitabine response in LUAD patients was assessed using the OncoPredict R package. Patients were stratified into A3C high and low expression groups based on the median expression level, and differences in predicted response were analyzed using the non-parametric Kruskal-Wallis test (*P* = 6.3e-18). **M** Pearson correlation analysis was conducted between A3C mRNA expression levels derived from the Broad DepMap database and gemcitabine sensitivity of matched cancer cell lines obtained from the GDSC database.

To investigate the clinical significance of A3C and compare all seven A3 paralogs, we initially examined their expression patterns in lung adenocarcinoma (LUAD, n = 524) and normal lung tissues (n = 486) based on TCGA and GTEx cohorts. Ridge plot indicated that A3C represented the most abundant cytidine deaminase among seven family members and was upregulated in tumor compared with normal tissues (Fig. 1A). In addition to mRNA levels, A3C protein expression was also significantly elevated in the LUAD cohort (Fig. 1B). The DepMap project can provide further information on essential genes that specifically affect the viability over thousands of cell lines by CRISPR knockout and RNAi screening. A highly negative dependency score indicates a gene candidate with lethal knockout phenotype. We noted that A3C exhibited a higher negative score compared with other six members, implying that A3C was the most significant member required for cell survival (Fig. 1C). The A3 family promotes genomic instability by inducing mutations, whereas p53 acts as the “guardian of the genome” by maintaining genomic integrity through DNA damage response, cell cycle arrest, and apoptosis. Thus, we embarked on the interplay between A3C and p53 status. Previous studies have reported that A3C expression is transcriptionally regulated by p53, suggesting a potential link between A3C dysregulation and TP53 mutation in cancer [27, 28]. To further confirm whether A3C was a predominant p53-responsive member among the A3 family, we applied curated *TP53* mutation status and expression data for each cell line from the DepMap datasets, and compared the expression levels of seven A3 genes between cell lines harboring mutant and wild-type p53. Interestingly, the expression levels of A3A, A3C, A3D, A3F, A3G, A3H but not A3B were significantly decreased in p53-mutant cancer cell lines when compared with cell lines possessing wild-type p53 (Fig. 1D). In an attempt to understand how A3 expression is regulated by p53, we analyzed three independent p53 ChIP-seq datasets from the ENCODE and GEO databases. In all three datasets, p53 binding was consistently observed within the A3C promoter/intronic region across different cell types and conditions. For instance, upon treatment with genotoxic agents such as cisplatin, or with the small molecule p53 activator Nutlin-3a, the width of p53-binding peaks at the A3C locus increased markedly, indicating an expansion of the binding region, suggesting that A3C is a primary transcriptional target of p53 in comparison with other A3 members. Moreover, we identified one p53-responsive element within the first intron as well as one putative element within the promoter of A3C (Fig. 1E, Supplementary Fig. 1). To validate our findings and exclude the potential dominant-negative effects of mutant p53 on wild-type p53, we employed A549 cells (harboring wild-type p53) and p53-null H1299 cells as a model system. Specifically, we generated p53-silenced A549 cells and H1299 cells expressing either wild-type p53 or representative hot-spot p53 mutants (R175H, R248W, R273H). We also confirmed that loss-of-function mutations of p53 markedly reduced the target gene of p21 (Supplementary Fig. 2). In accordance with database analysis, A3C was the most prominently expressed gene in two cell lines. Moreover, A549 cells showed higher mRNA levels of A3C than H1299 cells (Fig. 1F). In A549 cells, p53 knockdown caused reduced expression of A3C (Fig. 1G). In H1299 cells, ectopic expression of wild-type p53 but not hot-spot mutants concomitantly enhanced expression of A3C (Fig. 1H). Our results were in agreement with prior studies in Saos-2 (p53-defective) and HCT-116 (p53 wild-type) cells [28–30], thus A3C was a likely direct transcriptional target of p53.

To further explore the clinical relevance of A3C expression, we performed immunohistochemical analysis on FFPE tumor tissues obtained from three LUAD patients. Using a validated A3C antibody that specifically recognizes endogenous A3C protein [31], our results showed that A3C was expressed in both the nucleus and cytoplasm of epithelial cells located in bronchial wall and the alveoli of the lungs. Notably, A3C staining was observed in proliferating cells, including mitotic figures with characteristic double or spindle-shaped nuclei (Fig. 1I). These results further confirm the widespread and heterogeneous subcellular localization of A3C in active tumor cells. Further prognostic analysis using the Kaplan-Meier Plotter revealed that LUAD patients with high A3C expression had significantly shorter overall survival (OS) and first progression (FP) times, indicating that elevated A3C levels may be associated with poor clinical outcomes of LUAD (Fig. 1J). In particular, A3C-related gene set enrichment analysis (GSEA) in MSigDB gene collections showed that A3C strongly represented in signaling pathways enriched in gemcitabine resistance (Fig. 1K). The sensitivity of LUAD samples gemcitabine was calculated by oncoPredict, an R package for predicting drug response which was fitted to the gene expression data from the TCGA-LUAD cohort. A higher imputed sensitivity score represented lower drug sensitivity. Indeed, elevated expression of A3C could lead to a resistant phenotype to gemcitabine in patients (Fig. 1L). Moreover, we performed Pearson correlation analysis between the expression levels of APOBEC3 family members and the IC_50_ values of gemcitabine across cancer cell lines in the GDSC database. Among the seven APOBEC3 genes, only A3C (*P* < 0.0001) and A3D (*P* < 0.05) showed statistically significant correlations with gemcitabine sensitivity (Fig. 1M). Altogether, these results demonstrate that A3C is a prominent member of the APOBEC3 family of cytidine deaminases. Elevated A3C expression may contribute to altered gemcitabine sensitivity in lung adenocarcinoma.

### A3C intensifies replication stress and confers resistance to gemcitabine

**Fig. 2:**
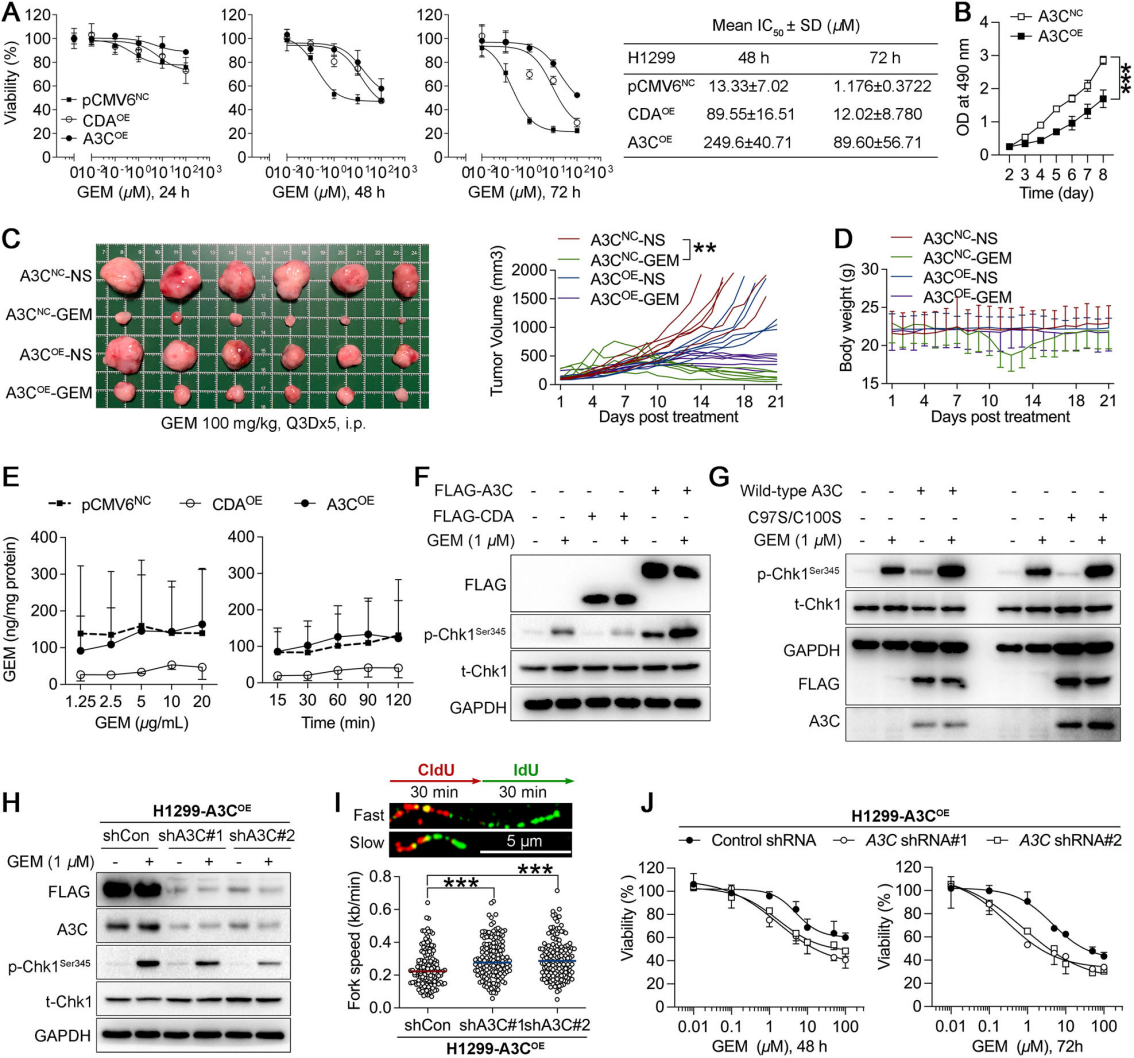
A3C induces gemcitabine resistance. **A** H1299 cells were stably transfected with empty vector pCMV6, CDA or A3C expression vectors. NC stands for negative control; OE stands for overexpression. Cells were challenged with 0.01, 0.1, 1, 10, 100 μM gemcitabine (GEM) for 24, 48 and 72 h. The cell viability was determined by MTS assay and IC_50_ values were calculated. Data are presented as the mean ± SD of three biologically independent experiments, one-way ANOVA does not reach statistical significance. **B** H1299 cells stably expressing A3C (A3C^OE^) or negative control cells (A3C^NC^) were initially cultured at a density as low as 500 cells/well in a 96-well plate. Cell growth was monitored using the method of MTS. The absorbance over time at OD490 values was measured every day. Cell expansion over time periods between two groups were compared by two-way repeated-measures ANOVA with the Greenhouse-Geisser correction and *post-hoc* analyses were performed by Šidák- adjusted multiple comparison tests. ***P < 0.001, compared with NC group. **C** For xenograft mouse model, H1299 cells stably transfected with empty vector pCMV6 (A3C^NC^) or A3C expression vector (A3C^OE^) were inoculated subcutaneously into the right flank of nude mice. After developing tumors, the mice were divided into four cohorts (n = 6) and treated with either (1) vehicle (normal saline, NS); (2) gemcitabine (100 mg/kg, i.p, Q3Dx5 or every third day for five doses). Tumor size was recorded using caliper measurements. Tumor growth over time periods among four groups were compared by two-way repeated-measures ANOVA with the Greenhouse-Geisser correction and *post-hoc* analyses were performed by Šidák- adjusted multiple comparison tests. **P = 0.0046 compared between gemcitabine-treated A3C-overexpressing xenografts and control xenografts, *P* = 0.2032 compared between A3C-overexpressing xenografts and control xenografts. **D** Daily mean body weights were determined and recorded. **E** H1299 cells were stably transfected with CDA, A3C or empty vector, the intracellular pharmacokinetics of gemcitabine was determined using UHPLC-MS/MS. Intracellular gemcitabine concentrations were normalized by protein concentration of whole-cell lysates. **F** H1299 cells expressing A3C, CDA or control vector were challenged with 1 μM gemcitabine for 6 h. Whole cell lysates were harvested for immunoblot of phospho-Chk1 (Ser 345), Chk1 and FLAG. GAPDH was used as an internal control. **G** H1299 cells expressing wild-type A3C or enzymatic mutant A3C were challenged with 1 μM gemcitabine for 6 h. Whole cell lysates were harvested for immunoblot of phospho-Chk1 (Ser 345), Chk1, A3C and FLAG. GAPDH was used as an internal control. **H** A3C expression was suppressed by shRNA-mediated knockdown in A3C-overexpressing H1299 cells. Cells were challenged with 1 μM gemcitabine for 6 h. Whole cell lysates were harvested for immunoblot of phospho-Chk1 (Ser 345), Chk1, A3C and FLAG. GAPDH was used as an internal control. shCon, non-targeting control shRNA. **I** A3C-overexpressing H1299 cells with A3C knockdown were processed for DNA fiber analysis. A total of 150 forks was scored for replication fork speed (kb/min) per group, representing the aggregate of two biologically replicates. Each biological replicate included three technical replicates (three microscope slides). Top: Schematic of the single-molecule DNA fiber tract labeling used to evaluate fork progression. Representative DNA fibers featured by the replication fork speed were shown. Scale bar: 5 µm. Bottom: Distribution of fork speed in H1299-A3C cells after transduction with control shRNA or shRNA targeting A3C. The horizontal line indicates the median fork speed. Statistical significance was determined by one-way ANOVA using Dunnett’s multiple comparisons test. ***P < 0.001 compared with shCon. **J** A3C-overexpressing H1299 cells with A3C knockdown were processed for gemcitabine treatment and MST assay. Downward sigmoidal dose-response curves were plotted by GraphPad Prism 9.3.1 software.

Gemcitabine is a nucleoside analogue that induces replication fork stalling and replication stress. A3 enzymes are well known for exacerbating replication stress through cytidine deamination on ssDNA, leading to replication fork stalling and genomic instability [32]. Previous studies have shown that tumors with low replication stress responded poorly to gemcitabine alone than tumors with high replication stress [33]. However, a recent study revealed that A3C facilitates resistance to gemcitabine by protecting cells against DNA replication stress. Thus, we sought to clarify how A3C influences gemcitabine sensitivity by modulating replication stress. To this end, we established H1299 cells stably overexpressing A3C and investigated their cellular response to gemcitabine treatment. Meanwhile, cytidine deaminase (CDA), a well-recognized drug metabolic enzyme by promoting the clearance and inactivation of gemcitabine, was used as a positive control for drug resistance test. Consistent with our expectations, enforced expression of A3C suppressed the cellular response to gemcitabine in both dose- and time-dependent manners. Based on the values of half-maximal inhibitory concentrations (IC_50_), CDA attenuated gemcitabine responsiveness but was less effective than A3C (Fig. 2A). We also found that H1299 cells harboring A3C exhibited decreased proliferation rate compared with empty vector-transfected counterparts (Fig. 2B). To further confirm our observations in vivo, we subcutaneously implanted H1299 cells stably expressing A3C or empty vector-transfected counterpart into the flank of mice, followed by gemcitabine treatment (100 mg/kg, i.p., five cycles at 3-day intervals). As a result, mice bearing A3C-proficient cells developed smaller xenografts and showed reduced response to gemcitabine compared to control xenografts (Fig. 2C). Meanwhile, gemcitabine treatment caused transient body weight loss in mice bearing control xenografts but not in mice implanted with A3C-overexpressing xenografts, possibly due to reduced tumor burden in control mice (Fig. 2D).

CDA and A3 are two different types of cytidine deaminases with different substrate preference. CDA confers gemcitabine resistance *via* deamination and inactivation of cytidines and analogues, while A3 enzymes deaminate the cytidines incorporated within 4-position of the cytosine base in single-stranded DNA polymers [34]. We next investigated the effect of A3C on the intracellular retention of gemcitabine using UHPLC-MS/MS. Cells stably expressing empty vector, CDA or A3C were incubated with gemcitabine ranging from 1.25 to 20 μg/mL for 2 h. In parallel, a fixed dose of gemcitabine (20 μg/mL) was added to cells with different exposure time ranging from 15 min to 2 h. As expected, the intracellular concentrations of gemcitabine in CDA-expressing cells were much lower than that in control cells, suggesting accelerated clearance of gemcitabine. However, A3C-expressing cells retained gemcitabine at levels similar to control cells (Fig. 2E). Our findings were consistent with previous study [7], excluding the possibility of metabolic elimination of gemcitabine by A3C. A3 genes have been shown to invoke DNA damage response through activating ATR/Chk1 signaling pathway [35–37]. Inhibition of ATR/Chk1 can sensitize tumor cells to A3 enzyme-dependent death [38–40]. Given that gemcitabine also induces replication stress and activates ATR/Chk1 to trigger DNA damage response, the combined activation of ATR/Chk1 signaling by A3 protein expression and gemcitabine treatment may synergistically enhance cellular tolerance to replication stress, thereby promoting tumor cell survival and resistance to gemcitabine. Thus, we further asked whether A3C could predispose to replication stress by evaluating the capacity of gemcitabine-induced Chk1 phosphorylation. Interestingly, we noted that gemcitabine-induced phosphorylation of Chk1 was attenuated in CDA-overexpressing cells, as the consequence of elevated metabolic inactivation of gemcitabine by CDA. In contrast, overexpression of A3C remarkably activated Chk1 in the presence of gemcitabine. Moreover, overexpression of A3C alone also had an augmented effect on Chk1 activation (Fig. 2F). C97 and C100 are two important zinc-coordinating residues of A3C, the simultaneous mutation of the two residues might result in a faithful inhibition of the catalytic activity of A3C compared with a single-residue mutation [41]. To determine whether the cytidine deaminase activity was essential for A3C-mediated Chk1 activation, we generated a catalytically inactive A3C by introducing C97S and C100S substitutions. As a result, cytidine deaminase-inactive A3C did not further increase constitutive Chk1 phosphorylation (Fig. 2G).

To further assess the functional requirement of A3C, we set out to silence A3C expression in A3C-overexpressing H1299 cells, while depletion of A3C expression partially reversed gemcitabine-induced Chk1 activation (Fig. 2H). Replication stress is defined as the slowing DNA synthesis or stalling of replication fork progression. To assess the replication stress underlying A3C-mediated Chk1 activation, we performed DNA fiber spreading analysis. Cells were sequentially labeled with CldU (25 μM, red) for 30 min and IdU (250 μM, green) for an additional 30 min. Loss of A3C resulted in a significantly higher DNA elongation rate compared to control cells, suggesting that A3C had a promoting effect on replication stress (Fig. 2I). In contrast, a recent study has demonstrated that A3C depletion slows replication fork progression in HPAF-II pancreatic cancer cell line but not in RPE1-hTERT p53⁻/⁻ cells, further emphasizing the necessity to consider p53 status and cellular context when interpreting the function of A3C in replication fork regulation [7]. Last but not least, A3C knockdown further restored the sensitivity of gemcitabine in A3C-overexpressing H1299 cells (Fig. 2J). Altogether, these results indicate that A3C confers resistance to gemcitabine through deaminase-dependent, replication stress-induced activation of Chk1 signaling.

### A3C confers gemcitabine resistance through a Chk1-dependent mechanism

**Fig. 3:**
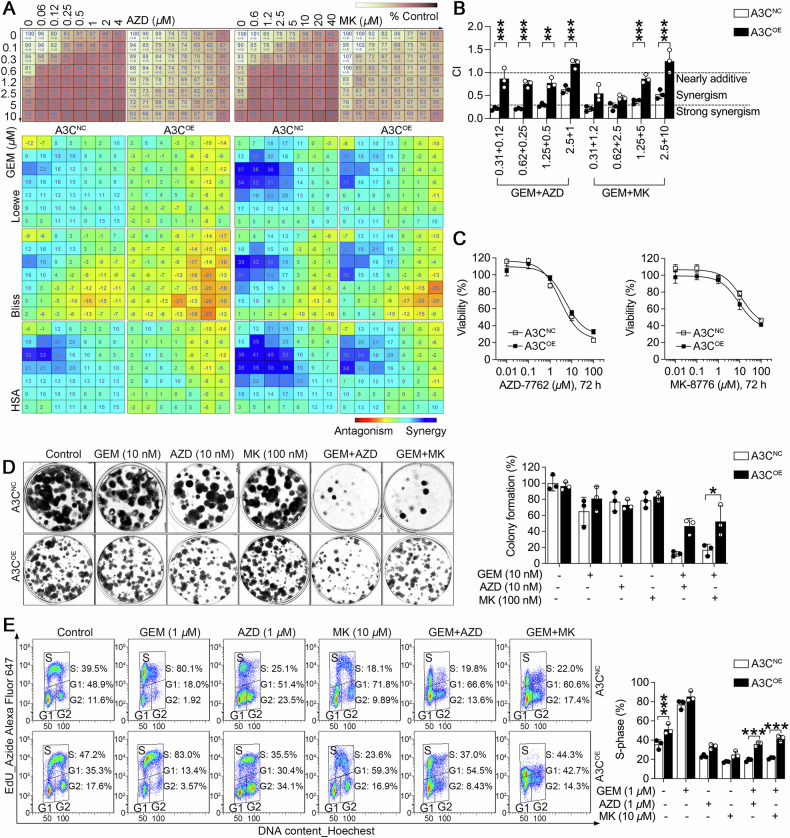
A3C sustains Chk1 signaling under Chk1 inhibitor via S-phase checkpoint activation. **A** A3C-overexpressing (A3C^OE^) or empty vector control (A3C^NC^) H1299 cells were incubated with Chk1 inhibitors (AZD-7762 or AZD, MK8776 or MK, x-axis) and gemcitabine (y-axis) in an 8 × 8 concentration checkerboard format for 72 h. Cell viability was determined by MTS assay. The experiment data on top panel (values were relative cell viability compared with control) were analyzed and calculated with Combenefit software. The predicted data were subtracted from the experimental data, yielding a final difference value for each combination. Data are expressed as mean values of three biologically independent experiments. The greater the difference value, the more synergistic that particular combination was. Three mathematical models (Loewe additivity, Bliss independence and Highest single agent) were used to robustly identify synergistic combinations. **B** Synergistic effect was also evaluated by CompuSyn based on the median-effect principle of Chou and Talalay. Combination index (CI) is widely accepted as an indicative of the degree of synergistic interaction. CI values less than 1.0 (horizontal line) correspond to a synergistic interaction. CI values below 0.3 indicate strong synergy. The data are presented as the mean ± SD by three biologically independent experiments. One-way ANOVA followed by *post-hoc* Tukey’s analysis was performed. **P = 0.0011, ***P < 0.001. **C** The cytotoxic effects of two Chk1 inhibitors on A3C-overexpressing (A3C^OE^) or empty vector control (A3C^NC^) H1299 cells were measured by MTS assay. **D** The effect of A3C on the colony-forming ability of H1299 cells in the presence of gemcitabine or/and two Chk1 inhibitors. The data are presented as the mean ± SD by three biologically independent experiments. One-way ANOVA followed by *post-hoc* Tukey’s analysis was performed. *P = 0.0217 between A3C^NC^ and A3C^OE^ H1299 cells co-treated with gemcitabine and MK-8776. **E** The effect of A3C on the DNA replication and cell cycle distribution of H1299 cells in the presence of gemcitabine or/and two Chk1 inhibitors for 24 h, which was assessed using a click reaction with Alexa Fluor 647 azide and Flow Cytometry. The percentages of a cell population in the different phases were marked on flow cytometry diagram. The percentage of cells at S-phase are presented as the mean ± SD by three biologically independent experiments. One-way ANOVA followed by *post-hoc* Tukey’s analysis was performed (***P < 0.001).

Preclinical and clinical studies have demonstrated synergistic antitumor activity for the combination of gemcitabine and Chk1 inhibitors, especially in p53-deficient tumors [42]. Thus, we further questioned whether A3C-induced gemcitabine resistance was Chk1 reliant. Using two Chk1 inhibitors that have been used in clinical testing, including AZD-7762 and MK-8776, we treated A3C-overexpressing H1299 cells and their control counterparts with two-drug regimen in 8 × 8 drug concentration matrices (x-axis for Chk1 inhibitors and y-axis for gemcitabine), and drug interaction was quantified and visualized by Combenefit® software. The color scale from blue to red represents the level of drug interaction from synergy to antagonism, based on the synergy score calculated by Loewe additivity, Bliss independence and highest single agent models. As shown in Fig. 3A, a dark blue region on the checkerboard attained across the upper left-hand panel, where sub-IC_50_ doses of gemcitabine and Chk1 inhibitors were used. We also utilized the term combined index (CI), which represents one of the most popular method for quantification of synergism or antagonism. The CI values using a fixed-dose combination of gemcitabine and Chk1 inhibitors were overwhelmingly below 0.3, which displayed a strong synergism between two drugs. Interestingly, the synergistic effects by concurrent exposure of gemcitabine and Chk1 inhibitors were impaired by A3C but not abolished at sub-IC_50_ concentrations (Fig. 3B). Noticeably, the effect of AZD-7762 on cell viability was more than 10-fold more potent than MK-8776, and the single-agent activity of two Chk1 inhibitors was not influenced by A3C levels (Fig. 3C). In addition, A3C expression also reduced the clonogenic potential of H1299 cells. Although the combination of gemcitabine with AZD-7762 or MK-8776 inhibited clonogenic survival in A3C-expressing cells, the effect was less pronounced than in control cells (Fig. 3D).

Since Chk1 activation occurred in A3C-overexpressing H1299 cells, we analyzed the role of A3C in checkpoint activation throughout the cell cycle. Incorporation of EdU, a thymidine analogue for labeling dividing cells during S-phase, was used in combination with Hoechst staining to measure DNA content by flow cytometry. Indeed, cells expressing high levels of A3C displayed increased fractions of EdU incorporation, suggesting delayed progression through S-phase of the cell cycle. In contrast, Chk1 inhibition by AZD-7762 or MK-8776 reduced EdU incorporation by overriding the replication stress checkpoint, resulting in impaired DNA synthesis. Gemcitabine treatment profoundly delayed cells at the G1/S border, which was substantially alleviated when combining gemcitabine with Chk1 inhibitors. However, the effects of Chk1 inhibitors alone or in combination with gemcitabine on the S-phase checkpoint could be partially reversed by A3C expression (Fig. 3E). Collectively, these results suggest that A3C triggers Chk1-dependent checkpoint control and compromises the gemcitabine-sensitization potential by Chk1 inhibition.

### A3C activates Chk1-dependent DNA damage response

**Fig. 4:**
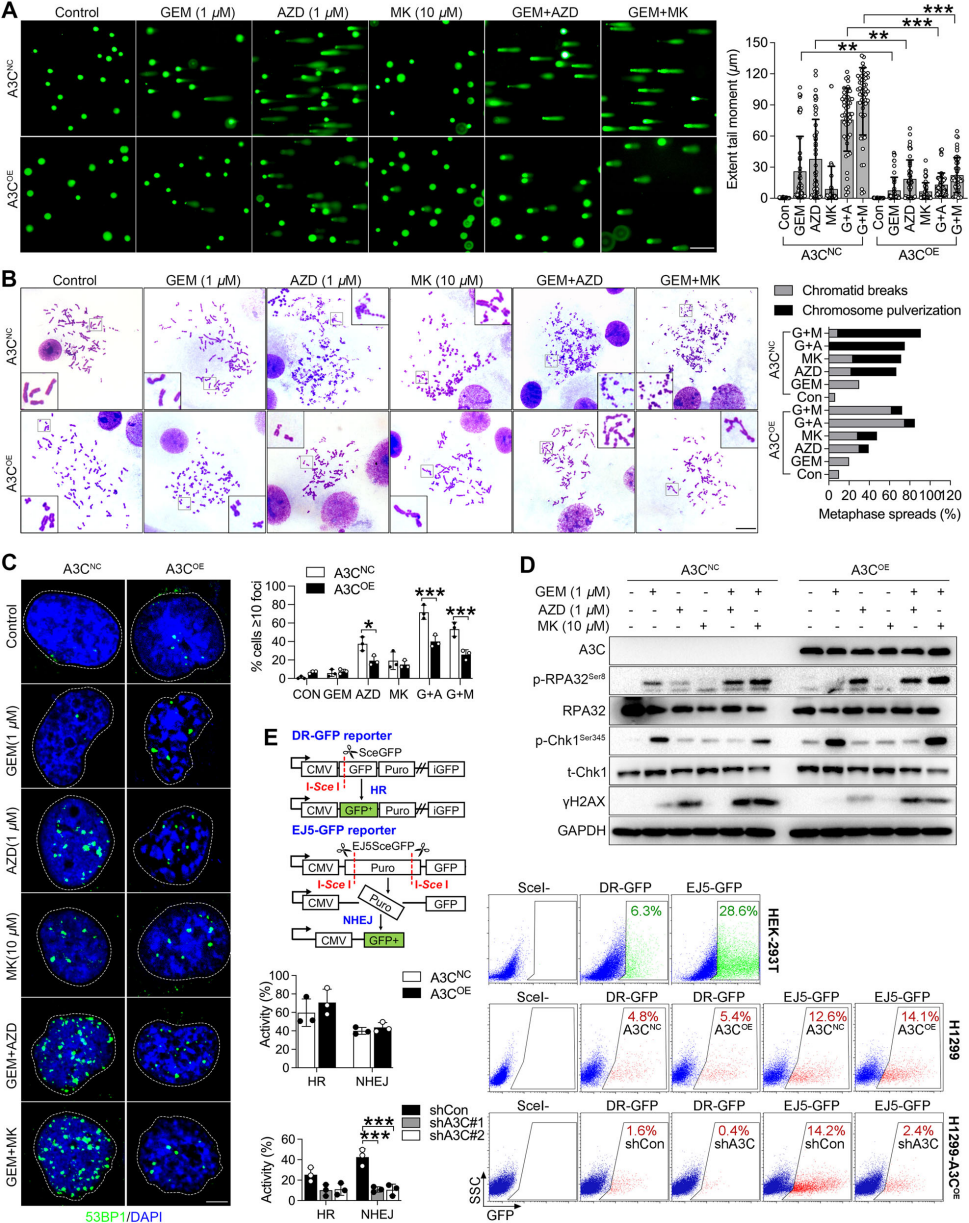
A3C triggers DNA damage response. **A** Representative images of comet assay measuring the amount of DNA damage in A3C-over expressing (A3C^OE^) or control (A3C^NC^) H1299 cells treated with gemcitabine or/and two Chk1 inhibitors for 24 h. A total of 50 comets was analyzed per group, representing the aggregate of two biologically independent replicates. Each biological replicate included three technical replicates (three replicate wells for one comet slide). Data are mean ± SD of tail moments (tail DNA% × length of tail). Scale bar, 200 μm. Scatter plots show all points, medians, and lower and upper quantiles. One-way ANOVA followed by *post-hoc* Tukey’s analysis was performed. **P = 0.0027 between A3C^OE^ and A3C^NC^ H1299 cells treated with GEM, **P = 0.0014 between A3C^OE^ and A3C^NC^ H1299 cells treated with AZD, ***P < 0.001). **B** Representative images of chromosome spreading assay to measure the chromosomal aberrations in A3C (A3C^OE^) or control (A3C^NC^) H1299 cells treated with gemcitabine or/and two Chk1 inhibitors for 24 h. A total of 20 metaphase spreads was analyzed per group, representing the aggregate of two biologically replicates. Each biological replicate included three technical replicates (three microscope slides). Data represent the percentage (%) of abnormal metaphase spreads (unrepaired chromatid breaks or chromosome pulverization) in a total of 20 metaphase spreads. Scale bar, 50 μm. **C** Representative images of pan-nuclear 53BP1 foci formation in A3C (A3C^OE^) or control (A3C^NC^) H1299 cells treated with gemcitabine or/and two Chk1 inhibitors for 6 h. Confocal images of indicated treatments with antibodies against 53BP1 (green). Nuclei were stained with DAPI (blue). 630× magnification images, scale bar represents 5 μm. Quantification of the number of 53BP1 foci per nucleus and the percentage of nucleus displaying more than 10 foci is plotted. Each bar represents the mean percentage (%) ± SD by three biologically triplicate experiments and at least 40 cells per group were analyzed for each experiment. Comparisons test was performed by one-way ANOVA followed by *post-hoc* Tukey’s test. *P = 0.0280, ***P < 0.001. (**D**) H1299 cells were transfected with A3C (A3C^OE^) or empty vector (A3C^NC^) for 48 h, and further challenged with gemcitabine (1 μM) in the presence or absence of Chk1 inhibitors (AZD, 1 μM; MK, 10 μM) for another 6 h. Whole cell lysates were harvested for detecting the levels of phosphorylated RPA32 (Ser8), phosphorylated Chk1 (Ser345), phosphorylated histone H2AX on serine 139 (γH2AX), total RPA32 and Chk1. GAPDH was used as loading control. **E** The effect of A3C on DNA repair activity using GFP-reporter assay and quantified by Flow Cytometry. Schematic representation of the HR reporter substrate DR-GFP and NHEJ reporter substrate EJ5-GFP. HEK-293T cells grown on 6-well plates were used as transfection efficiency control by transiently transfected with 1 μg/well pCBASceI and 1 μg/well DR-GFP/EJ5-GFP for 48 h and harvested for Flow Cytometry. H1299 control cells and A3C-overexpressing cells grown on 6-well plates were transiently transfected with 1 μg/well pCBASceI, 1 μg/well DR-GFP/EJ5-GFP for 48 h and harvested for Flow Cytometry. A3C-overexpressing cells and A3C knockdown cells established from A3C-overexpressing cells grown on 6-well plates were transiently transfected with 1 μg/well pCBASceI, 1 μg/well DR-GFP/EJ5-GFP for 48 h and harvested for Flow Cytometry. Data are expressed as the mean ± SD by three biologically triplicate values. Comparisons test was performed by unpaired *t*-test or one-way ANOVA (***P < 0.001).

Chk1 inhibition increased gemcitabine sensitivity by abolishing the intra-S-phase checkpoint, thereby exacerbating gemcitabine-induced replication stress [43]. In contrast, A3C promoted gemcitabine resistance by inducing replication stress and activating Chk1-dependent checkpoint signaling, which might enable cells to tolerate and repair replication-associated DNA damage. Thus, we next employed Comet assay and metaphase spreads to assess genomic integrity in gemcitabine-treated cells in the presence and absence of Chk1 inhibitors. As shown in Fig. 4A, single-agent gemcitabine hardly caused DNA fragmentation, but Chk1 inhibitors could be combined effectively with gemcitabine to achieve a higher degree of tail moment. However, the extensive tail moment induced by a combination of gemcitabine and Chk1 inhibition was substantially attenuated but not completely reversed in A3C-overexpressing cells, indicating that A3C is required to maintain Chk1 activity in order to counteract DNA damage under gemcitabine-induced replication stress. The accumulation of DNA damage subsequently causes chromosomal instability and aberrations. In agreement with previous report [43], metaphase chromosome spreads from control cells that had received combination therapy showed catastrophic chromosomal segments, which is termed chromosome pulverization or shattering, a phenomenon described as chromothripsis [44]. In comparison, the detectable levels of extensive chromosomal fragmentation were rare in cells overexpressing A3C, and breaks largely emerged on one of the chromatids, which displayed a discontinuity of a single chromatid or gap type (Fig. 4B). It was found that chromosome aberrations were formed in a temporal order from gap type to pulverization type [45]. Thus, A3C appeared to mitigate replication stress-induced structural chromosomal aberrations in a Chk1-dependent manner.

53BP1 is a key marker of DNA double-strand break (DSB) repair by accumulating at DSB sites to form visible nuclear foci that signal DNA damage and coordinate repair processes. Consistent with comet assay, a significant increase in nuclear 53BP1 foci was observed following combined treatment with gemcitabine and Chk1 inhibitors in control cells, but not in A3C-overexpressing cells, further supporting the notion that A3C might mitigate the accumulation of DNA damage (Fig. 4C). In addition to Chk1 phosphorylation, replication stress can also be indicated by accumulation of single-stranded DNA regions coated with RPA32. Phosphorylation of RPA32 serves as a sensitive marker of replication stress and DNA damage response. We observed that both RPA32 and Chk1 phosphorylation were further elevated when two Chk1 inhibitors were combined with gemcitabine, as well as potentiated γH2AX production, a molecular marker of cellular response to DNA breaks. Interestingly, phosphorylation of RPA32 and Chk1 was more pronounced in A3C-overexpressing cells, whereas γH2AX levels were attenuated, suggesting that A3C might facilitate γH2AX resolution through activation of Chk1-dependent DNA damage response (Fig. 4D). Homologous recombination (HR) and nonhomologous end joining (NHEJ) are two major pathways for repair of DNA double-strand breaks. Subsequently, we applied direct repeat (DR)-GFP and end joining (EJ)-GFP reporter systems to measure HR and NHEJ activity produced by the endonuclease I-*SceI*. DNA repair activity was measured by the fraction of GFP-positive cells, in which transfection efficiency was normalized to HEK-293T cells. Interestingly, we found A3C overexpression hardly affected repair activity, probably due to the stoichiometric requirements of the repair complex that involve cooperation with additional factors. However, knockdown of A3C in A3C-overexpressing cells decreased HR and NHEJ activities when compared with control cells, suggesting that A3C is essential but not a limiting factor for repair competence (Fig. 4E). Together, our studies reveal that A3C contributes to the maintenance of an effective DNA damage response through a Chk1-dependent pathway.

### A3C interacts with RNA helicase DDX5

**Fig. 5:**
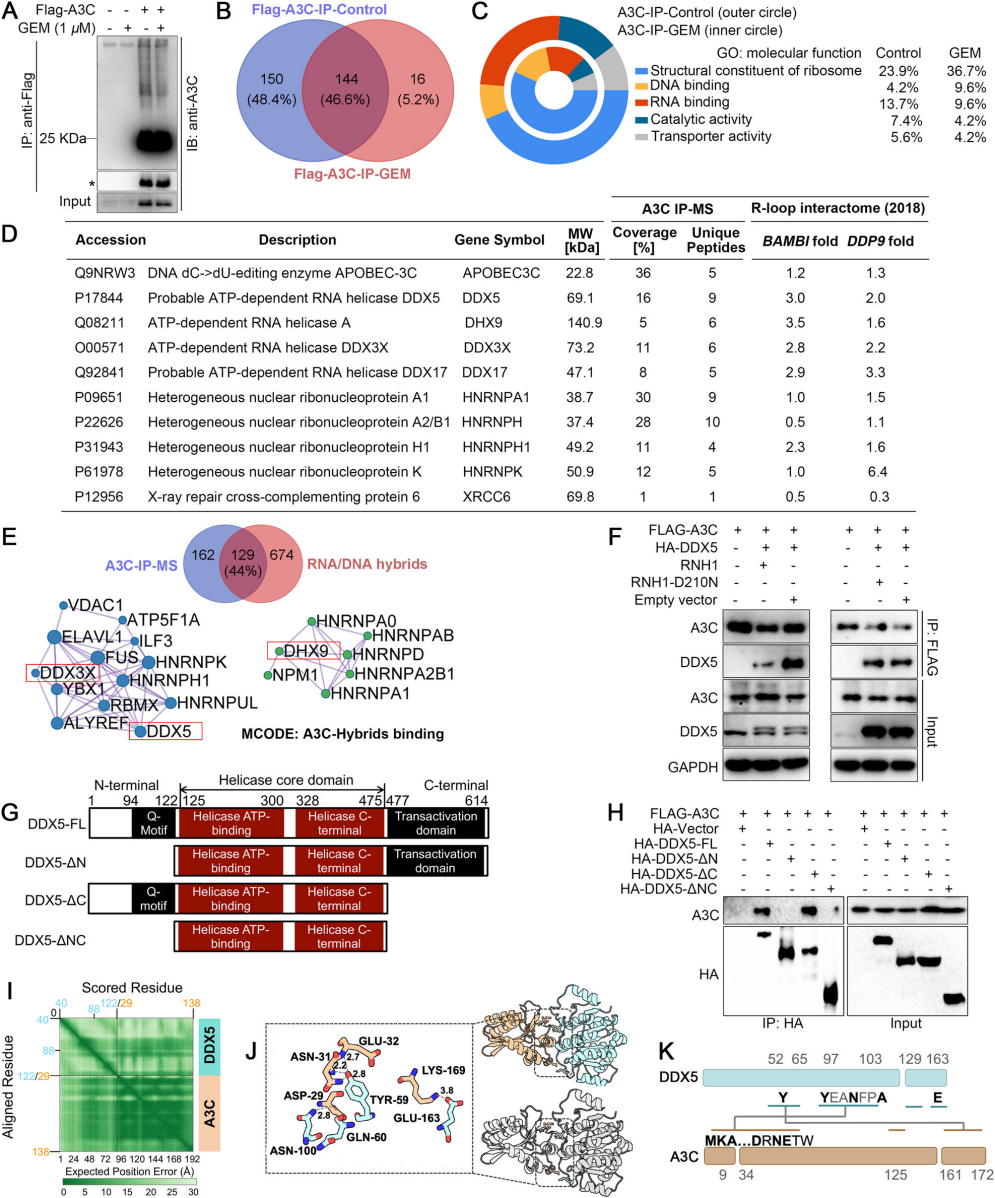
A3C proteins predominantly bind the N-terminal region of DDX5. **A** A3C pulldown and immunoblot analysis of H1299 cell lysates with or without gemcitabine treatment. * Shorter exposure time. **B** Venn diagram illustrated the intersection of A3C interactome with or without gemcitabine treatment. **C** Enrichment analysis of pathways based on Gene Ontology (GO). **D** A3C interactome was screened by mass spectrometry-based proteomics. The table showed the representative highest scored peptides that physically bind to A3C. Our AP-MS dataset was verified by published proteomic data (Cheung group, 2018) from RNA/DNA hybrids immunoprecipitation (DRIP) located at the *BAMBI* promoter and DPP9 3’ UTR. **E** A Venn diagram illustrated the intersection of A3C interactome (291, after removed keratins and other contaminants) with 803 proteins that interact with RNA/DNA hybrids published by Cheung group. Molecular Complex Detection (MCODE) was executed to identify key modules in the protein-protein interaction (PPI) network of A3C-hybrids binding. DDX5, DDX3X and DHX9 were highlighted in the highly interconnected clusters. **F** R-loop-dependent A3C-DDX5 interaction. H1299 cells grown on 10 cm dishes were co-transfected with 5 μg FLAG-tagged A3C and 5 μg HA-tagged DDX5, along with either 5 μg wild-type RNase H1 (ppyCAG_RNaseH1), the catalytically inactive D210N mutant, or an empty vector (pcDNA3.1). Cell lysates were analyzed by immunoprecipitation using anti-FLAG antibodies followed by immunoblots with the indicated antibodies. Input indicates 20% of pre-immunoprecipitated samples. **G** The full-length (FL), N-terminal (ΔΝ), C-terminal (ΔC) or both (ΔΝC) truncated forms of DDX5. **H** HEK-H293T cells were transfected with full-length (FL) HA-DDX5, or the DDX5 truncation mutants for 24 h. Lysates were incubated with immobilized HA. Affinity precipitated exogenous DDX5 was detected with anti-HA antibody, with the relative levels in the lysates also shown. **I** Display of the Predicted Aligned Error (PAE) for the AlphaFold-predicted A3C/DDX5 complex. DDX5 and A3C chains are labeled respectively in cyan and orange. A dark green tile designates a good prediction, and the region of DDX5 that is expected to be in proximity to A3C includes residues 50-100, while for A3C, the residue range is 30-180. Structure-based protein interaction interface analysis between A3C and DDX5. Image represents the predicted A3C-DDX5 complex, where interaction interface and hotspot residues are labeled. **J** Structure-based protein interaction interface between A3C (PDB ID: 3vow) and DDX5 (PDB ID: 4a4d) where interaction region and two hotspot residues (Tyr59/Tyr97) are labeled.

Next, we investigated what factors were involved in the tolerance of A3C-expressing cells in response to gemcitabine. We applied pull-down assay coupled to tandem mass spectrometry to identify interaction partners of A3C from H1299 lysates immunoprecipitated by FLAG-tagged A3C complexes in the presence and absence of gemcitabine treatment (Fig. 5A). We observed that approximately half of the pulled-down proteins were altered by gemcitabine (Fig. 5B). GO term molecular function enrichment analysis indicated that A3C-interacting proteins were mainly involved in the structural constituents of the ribosome, DNA binding, RNA binding, catalytic activity and transporter activity (Fig. 5C). We further identified the top-ranking proteins that potentially interacted with A3C (Fig. 5D). The hit interacting proteins were verified by published proteomic data from the Vivian Cheung lab, who identified R-loop interactome at 5’ end of the *BAMBI* gene and 3’ end of the *DDP9* gene [46]. These regions have been previously established by DNA: RNA hybrid immunoprecipitation (DRIP) and sequencing. Thus, the list also depicted shared proteins between A3C and R-loop-binding datasets. Typically, a set of DDX helicase family has attracted our great interest, which can regulate R-loop metabolism by unwinding RNA/DNA hybrids. Moreover, around 44% of A3C interactors had been found in proteins that bind to R-loops reported by Cheung group (Fig. 5E). Moreover, the recently published comprehensive interactome of the A3 family also identified a robust A3C-DDX5 interaction [47]. Functional enrichment analysis and protein-protein interaction mapping for densely connected clusters by the MCODE algorithm revealed that DDX5 had a higher degree of node connections compared with other DDXs. Besides, DDX5 was also identified in R-loop interactome by R-loop proximity proteomics [48]. Thus, DDX5 was screened as a functional partner of A3C for further investigation.

To verify the interaction between A3C and DDX5 and assess whether it is dependent on R-loop structures, we co-expressed RNaseH1 or a catalytically inactive RNaseH1 mutant (D210N) with A3C and DDX5 for reducing cellular R-loop levels. We observed that the interaction between A3C and DDX5 was diminished upon expression of active but not the inactive RNaseH1 expression, suggesting that interactions were mediated by R-loops (Fig. 5F). Next, we began to address the binding region behind the A3C/DDX5 interaction. As DDX5 is a relatively large, multidomain protein, we initially determined whether a particular region of DDX5 was required for the interaction with A3C. Full-length, N- and/or C-terminal truncated versions of DDX5 were expressed in HEK-293T cells and their ability to interact with immobilized A3C was investigated (Fig. 5G). We found that the removal of the N-terminal 122 aa of DDX5 was sufficient to dramatically reduce the proportion of A3C that was precipitated by DDX5 (Fig. 5H). To gain structural insight into the interaction interface between A3C and DDX5, we employed AlphaFold-Multimer, a deep learning-based tool designed to predict protein complex structures with high accuracy. Notably, the deaminase domain of A3C (residues 29-138) and the N-terminal region of DDX5 (residues 40-122) yielded the highest confidence prediction scores, suggesting a preferential interaction between these regions (Fig. 5I). To identify potential interacting hotspots between A3C and DDX5, we performed computational modelling of protein-protein interaction by rigid-body docking approach. The orange ribbon-like structure represents the A3C deaminase, while the cyan ribbon-like structure represents the DDX5 helicase (Fig. 5J). Consistent with our observations, in silico simulations showed that the A3C protein primarily interacts with the loop region of the N-terminal of DDX5 (residues 52-65 and 97-103), with additional contributions from salt bridges formed at the C-terminal (Fig. 5K). Together, these data indicate that A3C proteins preferentially interact with the N-terminal region of DDX5, which displays a pivotal role in their interaction.

### A3C facilitates Chk1-dependent R-loop resolution

**Fig. 6:**
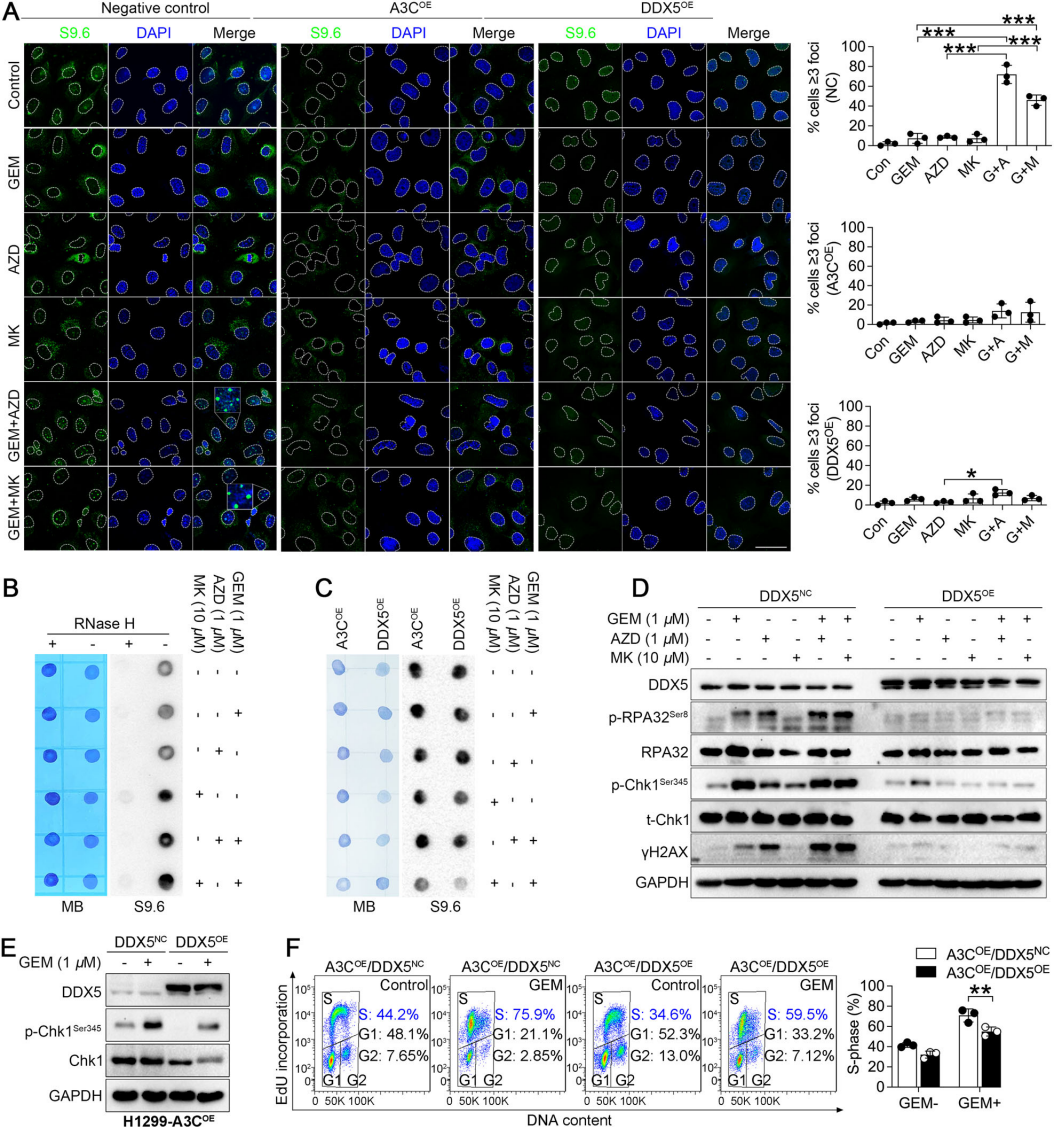
A3C and DDX5 attenuated Chk1 inhibition-induced R-loop accumulation in cells treated with gemcitabine. **A** Immunofluorescent staining with the S9.6 antibody showed the R-loop formation (green foci) in response to gemcitabine or/and Chk1 inhibitors in H1299 cells expressing A3C, DDX5 or their control counterparts (pCMV6). Nuclei were counterstained with DAPI (blue). Scale bar represents 50 μm. Quantification of the number of S9.6 foci per nucleus and the percentage of nucleus displaying more than 3 foci is plotted. Each bar represents the mean percentage (%) ± SD by three biologically triplicate experiments and at least 40 cells per group were analyzed for each experiment. Comparisons test was performed by one-way ANOVA followed by *post-hoc* Tukey’s test. *P = 0.0112, ***P < 0.001. **B** H1299 cells were treated with gemcitabine or/and Chk1 inhibitors for 6 and genomic DNA in a dilution of 400 ng/μL was extracted for assessing R-loop levels. Nuclear samples digested with or without RNase H (0.5 U) were loaded onto nylon membranes (2.5 μL per dot) and probed with S9.6 antibody. **C** Dot-blot analysis for A3C or DDX5-overexpressing cells treated with gemcitabine or/and Chk1 inhibitors. **D** H1299 cells were transfected with DDX5 (DDX5^OE^) or empty vector (DDX5^NC^) for 48 h, and further challenged with gemcitabine (1 μM) in the presence or absence of Chk1 inhibitors (AZD, 1 μM; MK, 10 μM) for another 6 h. Whole cell lysates were harvested for detecting the levels of phosphorylated RPA32 (Ser8), phosphorylated Chk1 (Ser345), phosphorylated histone H2AX on serine 139 (γH2AX), total RPA32 and Chk1. GAPDH was used as loading control. **E** A3C-overexpressing H1299 cells were transfected with DDX5 (DDX5^OE^) or empty vector (DDX5^NC^) for 48 h and challenged with gemcitabine (1 μM) for 6 h. Whole cell lysates were harvested for detecting the levels of phosphorylated Chk1 (Ser345) and Chk1. **F** EdU incorporation assay was performed in A3C^OE^/DDX5^NC^ and A3C^OE^/DDX5^OE^ H1299 cells. All the cells were stimulated with or without gemcitabine for 24 h. Data are expressed as the mean ± SD by triplicate assays. One-way ANOVA followed by *post-hoc* Tukey’s analysis was performed. **P = 0.0098.

R-loop induction may pose structural obstacles for genome integrity and lead to DNA breaks, which can be resolved by DEAD box helicases [9]. The association between DNA deaminase and RNA helicase raised the possibility that A3C might be likely involved in R-loop homeostasis. To test this hypothesis, we conducted immunofluorescent analysis that specifically detected R-loops using the S9.6 antibody. Interestingly, we observed S9.6 foci formation in nuclei of H1299 cells challenged by co-treatment of gemcitabine and Chk1 inhibitors. In comparison, ectopic expression of A3C or DDX5 was sufficient to resolve R-loops under gemcitabine exposure and Chk1 inhibition, suggesting that A3C could suppress R-loop accumulation in a Chk1 activity-dependent manner (Fig. 6A). In addition to immunofluorescence staining, dot blot was performed to measure R-loops. Initially, RNase H (0.5 U, 37 °C, 1 h) could efficiently digest the RNA strand of RNA:DNA hybrids and diminished R-loop signal intensities, suggesting the specificity of S9.6 antibody (Fig. 6B). In line with immunofluorescence assay, the enhanced R-loop levels by gemcitabine and Chk1 inhibitors were attenuated upon overexpression of A3C or DDX5 (Fig. 6C). Thus, we next examined whether the activation of Chk1-dependent DNA damage response signaling could be attenuated by DDX5. Surprisingly, the activation of RPA32, Chk1 and accumulation of γH2AX were completely abrogated by DDX5, indicating that DDX5 might further resolve R-loop-associated replication stress and DNA damage for inducing gemcitabine resistance (Fig. 6D). Moreover, DDX5 overexpression compromised gemcitabine-mediated Chk1 phosphorylation (Fig. 6E) and S-phase checkpoint activation (Fig. 6F) in A3C-overexpressing H1299 cells. Collectively, these data indicate that A3C potentially interacts with DDX5 to coordinate the dynamic regulation of Chk1 activation and R-loop homeostasis.

### DDX5 resolves R-loops to enable A3C-mediated gemcitabine resistance

**Fig. 7:**
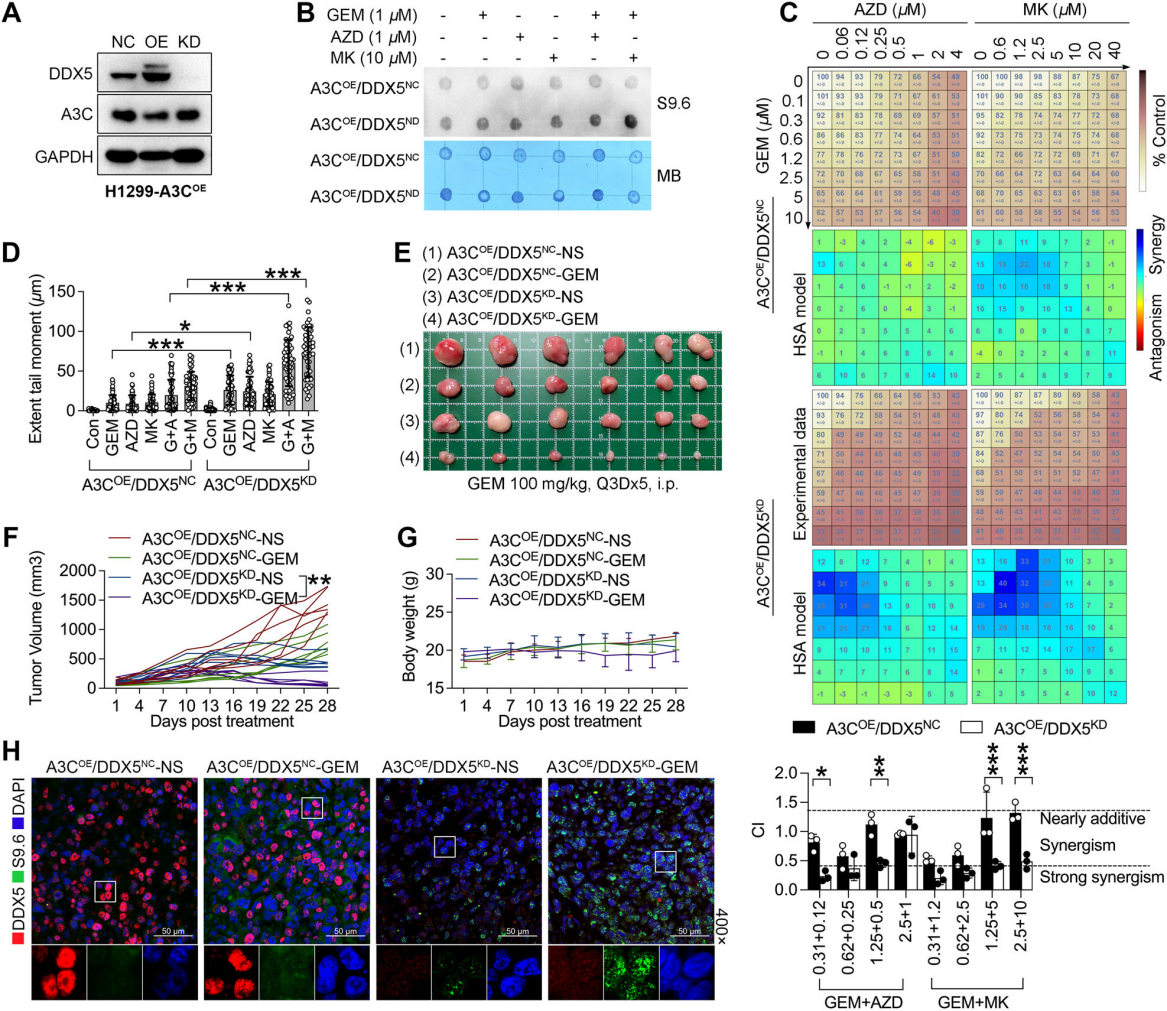
DDX5 is essential for A3C-mediated gemcitabine resistance by promoting R-loop clearance. **A** Further modulation of DDX5 expression in A3C-overexpressing H1299 cells for evaluating the functional interplay between A3C and DDX5 (overexpression, OE; knockdown, KD; NC, negative control). **B** A3C^OE^/DDX5^NC^ and A3C^OE^/DDX5^KD^ cells were co-treated with gemcitabine or/and Chk1 inhibitors for 6 h, and dot-blot analysis was performed for detecting R-loop levels using S9.6 antibody. Methylene blue was a loading control. **C** Drug sensitivity interaction between gemcitabine and two Chk1 inhibitors were evaluated in A3C^OE^/DDX5^NC^ and A3C^OE^/DDX5^KD^ H1299 cells using Combenefit and CompuSyn programs. *P = 0.0359, **P = 0.0085, ***P < 0.001. **D** Comet assay in A3C^OE^/DDX5^NC^ and A3C^OE^/DDX5^KD^ H1299 cells. Data are expressed as the mean ± SD. One-way ANOVA followed by *post-hoc* Tukey’s analysis was performed (*P = 0.0279, ***P < 0.001). Scatter plots show all points, medians, and lower and upper quantiles. **E** For xenograft mouse model, A3C^OE^/DDX5^NC^ and A3C^OE^/DDX5^KD^ cells were inoculated subcutaneously into the right flank of nude mice. After developing tumors, the mice were divided into four cohorts (n = 6) and treated with either (1) vehicle (normal saline); (2) gemcitabine (100 mg/kg, i.p., Q3Dx5 or every third day for five doses). **F** Tumor size was recorded using caliper measurements. Tumor growth over time periods among four groups were compared by two-way repeated-measures ANOVA with the Greenhouse-Geisser correction and *post-hoc* analyses were performed by Šidák- adjusted multiple comparison tests. **P = 0.0016 between gemcitabine-treated A3C^OE^/DDX5^KD^ xenografts and control xenografts. **G** Daily mean body weights were determined for all groups. **H** Representative fluorescence images of DDX5 (red) and S9.6 (green foci) staining positivity from xenograft tissues at 400× magnification. Nuclei were stained with DAPI. Scale bar represents 50 μm.

To further investigate DDX5 as a potential downstream effector of A3C, we performed DDX5 knockdown in A3C-overexpressing H1299 cells (Fig. 7A). Indeed, DDX5 depletion restored R-loop accumulation in A3C-proficient cells treated with gemcitabine and/or Chk1 inhibitors (Fig. 7B). Moreover, the degree of Chk1 inhibition-mediated gemcitabine sensitization was also increased by downregulation of DDX5, further supporting the essential role of DDX5 in A3C-mediated gemcitabine resistance in a Chk1-dependent context (Fig. 7C). As expected, the levels of DNA lesions induced by gemcitabine with Chk1 inhibition were remarkably reinforced. These results indicate that DDX5 might cooperate with A3C to suppress replication stress-associated DNA damage through R-loop resolution (Fig. 7D).

To confirm the dependency of DDX5 for A3C-mediated drug resistance and R-loop regulation in vivo, we further established a xenograft mouse model using A3C-overexpressing cells that were either DDX5-proficient or deficient (Fig. 7E). Our results showed that DDX5 knockdown could restore gemcitabine sensitivity in A3C-overexpressing xenografts (Fig. 7F). Besides, the body weight of tumor-bearing mice from gemcitabine-treated A3C^OE^/DDX5^KD^ xenografts was slightly declined (Fig. 7G). We next to validate whether DDX5 knockdown could promote R-loop accumulation for recapturing gemcitabine sensitivity. Consistent with the in vitro studies, depletion of DDX5 exhibited an increased nuclear S9.6 immunofluorescence signal in gemcitabine-administered A3C-proficient xenografts (Fig. 7H). Together, these observations suggest that the tumor cells with high A3C expression also require high expression levels of DDX5 in R-loop components to mitigate the cytotoxic effect of gemcitabine against R-loop accumulation, substantiating the relevance of DNA deaminase-RNA helicase cascade to gemcitabine resistance.

## Discussion

Overexpression of A3 enzymes in tumor cells has been associated with increased chemoresistance by driving a high mutational burden that enables clonal evolution and adaptive survival under therapeutic exposure. This phenotype contributes to the emergence of drug-tolerant persister cells [49]. Using CRISPR/Cas9-based genetic screening, a new study has reported the role of A3C and A3D in gemcitabine resistance [7]. In comparison, our genetic and biochemical studies further demonstrated that A3C was essential for the fitness of p53-deficient cells in response to gemcitabine and molecular details of how A3C protected against gemcitabine-induced DNA replication stress. Overall, we identified a previously unrecognized A3C-DDX5-R-loop resolution signaling axis that limited the cytotoxic efficacy of gemcitabine. Our findings establish a mechanistic model wherein A3C supports chemoresistance via an R‑loop resolution complex that safeguards Chk1-mediated DNA damage response in correlation to p53 status.

PCR methods can reliably detect APOBEC3C transcripts in a wide range of human tissues and cancer cell lines, and A3C is often the most abundantly expressed at the mRNA level. However, attempts to validate endogenous A3C protein expression by immunoblot have frequently required either epitope-tagged constructs or ectopic overexpression systems, due to the limited sensitivity and specificity of available antibodies. Anderson et al. systematically compared five commercially available anti-A3C antibodies and found that only one reagent, a polyclonal antibody from Proteintech was capable of reproducibly detecting both transfected and endogenous A3C protein in immunoblot experiments [31, 50]. Moreover, this limitation extends beyond A3C to other family members, such as A3A, A3B, etc [2, 51]. In our study, N-terminal FLAG-fusion but not endogenous A3C could be readily detected by this antibody. To this end, we established A3C-overexpressing H1299 cells, and subsequently manipulated A3C expression via A3C knockdown. Immunoblots incubated with anti-A3C antibody against A3C and FLAG both showed reduced band intensity after A3C knockdown, further supporting the specificity and reliability of the antibody signals. Thus, we utilized an A3C overexpression system for gain-of-function of A3C to investigate the relationship between A3C expression and gemcitabine resistance.

Replicative stress refers to the slowing of DNA replication featured by a stalled replication fork, and phosphorylated Chk1 is used as an activation marker of replication stress [52]. Gemcitabine leads to replication stress by errors in DNA synthesis as a fraudulent base and exhaustion of dNTP pools as a ribonucleotide reductase inhibitor [53]. Our study revealed that A3C overexpression could induce endogenous replication stress as evidenced by constitutively elevated Chk1 phosphorylation, stalled replication forks and S-phase arrest, which stands in opposition to prevailing notion that tumors with high replicative stress could be further enhanced by chemotherapeutics, including gemcitabine [33, 54]. Indeed, scientists have deciphered how DNA strand can be attacked by APOBEC3s during DNA replication [55]. It was possible that replication stress by enforced A3C expression rendered tumor more reliant on Chk1 kinase to stabilize and repair replication forks [56]. In this context, a positive feedback loop ensured an accumulation of under-replicated DNA that presumably limited the availability of nucleosides as well as the nucleoside analog gemcitabine [57]. Therefore, the extent of replication stress could not substantiate therapeutic sensitivity. The discrepancies were not initially determined by replication stress response induced by A3C, but rather resulted from differences in DNA damage response triggered by R-loop accumulation.

Broadly, p53-defective tumors are highly reliant on Chk1-mediated checkpoint surveillance for overcoming the replication defects and triggering DNA damage response. Earlier evidence has shown that checkpoint abrogation-mediated forced mitotic entry is not a common determinant of effective synergism [43, 58]. Our studies also shed light on the role of R-loop biology in DNA damage response and therapeutic index of chemotherapy combined with inhibitors targeting DNA damage response pathways. Notably, replication stress can increase the chance of a collision between replication and transcription and promote R-loop formation [59, 60]. In support of this possibility, we unexpectedly identified the role of A3C in R-loop resolution. As a primary tumor mutator, A3B has just been demonstrated to be associated with R-loop metabolism. However, the effect of A3B on R-loop formation and resolution has been shown to be inconsistent between studies. McCann et al. reported that knockout of A3B increased R-loop levels while overexpression of A3B decreased JQ1 (BET bromodomain inhibitor, a well-known R-loop inducer)-triggered R-loop levels, suggesting that A3B could clear R-loops [14]. On the contrary, Zong et al. found that knockout of A3B reduced R-loop levels while overexpression of A3B elevated endogenous R-loop levels using the same detection method by S9.6 signal intensity [40]. In consensus with McCann’s study, mass spectrometry suggested that A3C bound to key enzymes responsible for clearing R-loop. DDX5 was identified as an interacting partner of A3C by our and other studies, which has been recognized as a R-loop resolving enzyme [9, 46]. Using DDX5 as positive control, our results indicated that overexpression of A3C could attenuate R-loop accumulation upon combination therapy. It is noteworthy that the combination of gemcitabine with Chk1 inhibitors induced higher R-loop levels, which can serve an alternative molecular basis for chemosensitization. It is plausible that gemcitabine activates Chk1 to stabilize the replication fork and prevent fork progression, while abrogation of Chk1 activity can increase CDC7-dependent replication initiation by unrestrained origin firing [58, 61, 62]. Mechanistically, origin firing usually occurs close to the transcription start region of highly transcribed genes [63], whereas dysregulated origin firing further increases the number of head-on transcription-replication collisions and stabilizes R-loops [16]. Consequently, these disruptive DNA structures provide maximal extent of DNA damage and the levels of chemopotentiation.

We also found that A3C participated in DNA repair to facilitate R-loop resolution. Interactome analysis revealed that A3C also interacted with DSBs repair proteins such as XRCC6. Interestingly, a recent study has emphasized that A3C can be excluded from areas of DNA damage for active DNA repair through subnuclear location in nucleolus, which serves a protective machinery for the host cell genome integrity [27]. However, we noticed that A3C still had a pan-nuclear retention and localization patterns apart from nucleolus in response to genotoxic stimuli. On the one hand, the authors successfully enriched the protein fraction from nucleolus, and compared A3C content between whole cell lysates and nucleolar lysates. Unfortunately, the information between whole nuclear lysates and nucleolar lysates is missing. On the other hand, if A3C had already distributed in damaged chromatin, its exclusion from DNA breaks could be transiently ordered for recruitment of other repair factors [64]. Studies have shown that DDX5 is excluded from DNA break sites before R-loop being resolved, which occurs from existing chromatin-bound DDX5 but not newly recruited DDX5 at DSBs [9], then further understanding of A3C in R-loop-triggered DNA repair and underlying pathways of A3C-mediated DSBs repair for R-loop processing would be warranted.

In conclusion, the present study delineates further information regarding the effect of A3C on the therapeutic effect gemcitabine in p53-deficient H1299 cell model beyond protecting cells against DNA replication stress [7]. Since both A3C and A3D have been reported to be involved in gemcitabine resistance, our future studies aim to systematically profile the expression of all seven APOBEC3 paralogs and assess their individual and combined contributions. This will help to precisely attribute the observed phenotypes to A3C and further clarify the functional specificity within the A3 family. Together, these results suggest that A3C binding to DDX5 helps to invoke R-loop metabolism and Chk1-dependent DDR cascade, which can be an alternative mechanism for anticancer response of gemcitabine. Given the breadth of A3C overexpression in cancer, A3C may be used to assess pharmacodynamics and therapy response for single agent of gemcitabine and combination regimen with Chk1 inhibitors.

## Supplementary information

Supplementary Information

Raw blots

## Data Availability

Data will be made available from the supplementary materials and the corresponding author upon reasonable request.
