# Supplementary material for: APOBEC3C coordinates DDX5 in R-loop resolution and dynamic control of Chk1-mediated stress-responsive circuitry as a prerequisite for gemcitabine resistance in p53-deficient cells
Source: Cell Death Dis. 2026 Jan 7;17(1):6. doi: 10.1038/s41419-025-08215-6 (PMC12780011; doi:10.1038/s41419-025-08215-6)
Supplement: Supplementary file 1 — Supplementary Information [file 41419_2025_8215_MOESM1_ESM.pdf]

Supplementary Information

APOBEC3C coordinates DDX5 in R-loop resolution and dynamic control of Chk1-mediated stress-responsive circuitry as a prerequisite for gemcitabine resistance in p53-deficient H1299 cells

Li Tao [1, 2 *], Yang Zhao [1, 3], Zhuangzhaung Jiang [1, 4], Shujing Kong [1, 2], Yanlin Ding [1, 2], Tengyang Ni [1, 5], Weimin Wang [2, 6], Yanqing Liu [2*]

1. Department of Pharmacy, College of Medicine, Yangzhou University, Yangzhou 225009, Jiangsu, China

2. The Key Laboratory of Syndrome Differentiation and Treatment of Gastric Cancer of the State Administration of Traditional Chinese Medicine, Yangzhou University, Yangzhou 225009, Jiangsu, China

3. Department of Medicine, Linfen Vocational and Technical College, Linfen 041000, Shanxi, China

4. School of Traditional Chinese Pharmacy, China Pharmaceutical University, Nanjing 211198, Jiangsu, China

5. Department of Biochemistry and Structural Biology, University of Texas Health Science Center at San Antonio, San Antonio TX 78229, USA

6. Department of Oncology, Yixing Hospital Affiliated to Medical College of Yangzhou University, Yixing 214200, Jiangsu, China

* Correspondence to: Li Tao, Department of Pharmacy, College of Medicine, Yangzhou University, 136 Jiangyang Avenue, Yangzhou, Jiangsu, 225009, China. E-mail: imlitao@yzu.edu.cn; Yanqing Liu, The Key Laboratory of Syndrome Differentiation and Treatment of Gastric Cancer of the State Administration of Traditional Chinese Medicine, Yangzhou University, 136 Jiangyang Avenue, Yangzhou 225009, Jiangsu, China. E-mail: liuyq@yzu.edu.cn. Tel: +86-0514-8793-7083, Fax: +86-0514-8793-7133.

Table of Content

Figure S1..**Page S3**

Figure S2..**Page S5**

Table S1..**Page S6**

Table S2..**Page S7**

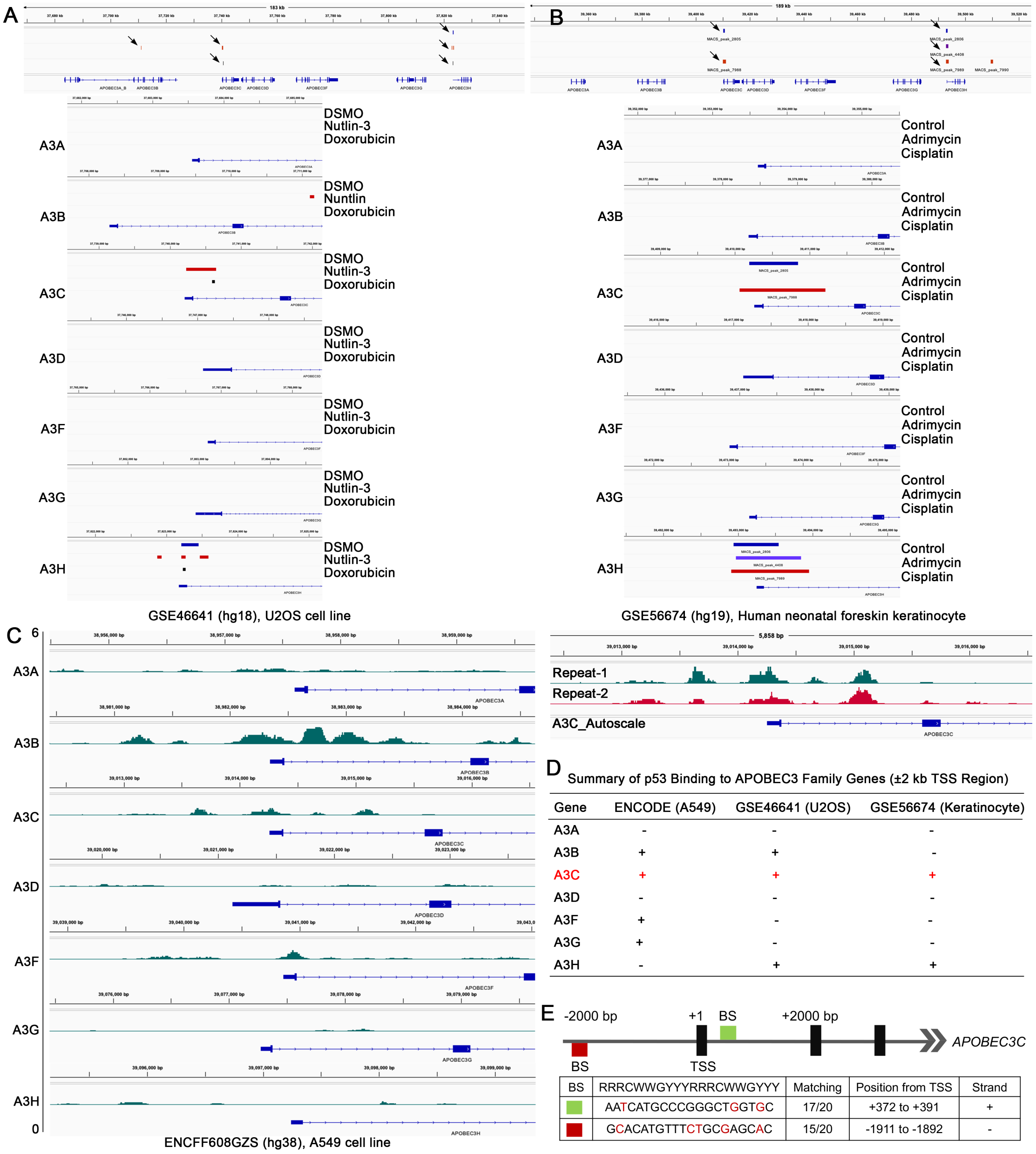

Gene	ENCODE (A549)	GSE46641 (U2OS)	GSE56674 (Keratinocyte)
A3A	-	-	-
A3B	+	+	-
A3C	+	+	+
A3D	-	-	-
A3F	+	-	-
A3G	+	-	-
A3H	-	+	+

BS	RRRCWWGYYYRRRCWWGYYY	Matching	Position from TSS	Strand
(green)	AATCATGCCCGGGCTGGTGC	17/20	+372 to +391	+
(red)	GCACATGTTTCTGCGAGCAC	15/20	-1911 to -1892	-

Supplementary Figure 1. Public p53 ChIPseq datasets. APOBEC3 genes are arrayed in tandem on chromosome 22 and visualized by IGV. Binding profiles of p53 at the transcription start site (TSS) of APOBEC3 family between 2,000 bp upstream and 2,000 bp downstream by public ChIP-seq datasets and were visualized using the Integrative Genomics Viewer (IGV). **(A)** GEO dataset GSE46641 (U2OS cells, hg18) under three conditions (DMSO, Nutlin, Doxorubicin) and **(B)** GEO dataset GSE56674 (Human neonatal foreskin keratinocytes, hg19), showing MACS2-called peaks under control, Adriamycin, and Cisplatin treatment. **(C)** ChIP-seq tracks from ENCODE

(ENCFF608GZS, A549 cells, hg38). **(D)** Among all APOBEC3 family members, A3C consistently showed p53 binding signals or peaks within TSS in all three datasets. **(E)** Sequence of the predicted p53 response element in this region (AATCATGCCCGGGCTGGTGC, +372 to +391 from TSS) matched 17 out of 20 bases with the p53 consensus motif, as well as one putative element within the promoter by JASPAR.

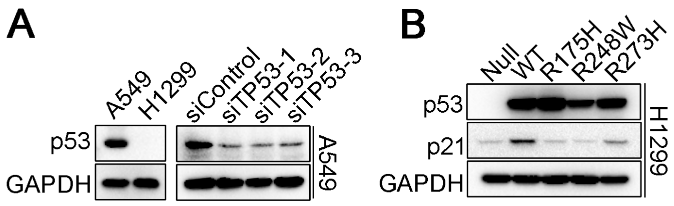

Supplementary Figure 2. Cell lines with different p53 status. A p53 regulatory cell model was established by silencing endogenous p53 in A549 cells (TP53 wild-type) using siRNA **(A)**, and by reintroducing wild-type or mutant p53 (R175H, R248W, R273H) into p53-null H1299 cells **(B)**. Protein expression of p53 and its downstream effector p21 was assessed by Western blot assay.

Supplemental Table 1. Primer sequences for APOBEC3 gene family. Primer sequence used for quantitative real-time PCR analysis of mRNA expression of seven APOBEC3 family members.

Primer name	Sequence	nt
A3A_F	GAGAAGGGACAAGCACATGG	20
A3A_R	TGGATCCATCAAGTGTCTGG	20
A3B_F	GACCCTTTGGTCCTTCGAC	19
A3B_R	GCACAGCCCCAGGAGAAG	18
A3C_F	AGCGCTTCAGAAAAGAGTGG	20
A3C_R	AAGTTTCGTTCCGATCGTTG	20
A3D_F	ACCCAAACGTCAGTCGAATC	20
A3D_R	CACATTTCTGCGTGGTTCTC	20
A3F_F	CCGTTTGGACGCAAAGAT	18
A3F_R	CCAGGTGATCTGGAAACACTT	21
A3G_F	CCGAGGACCCGAAGGTTAC	19
A3G_R	TCCAACAGTGCTGAAATTCG	20
A3H_F	AGCTGTGGCCAGAAGCAC	18
A3H_R	CGGAATGTTTCGGCTGTT	18
GAPDH_F	CGAGATCCCTCCAAAATCAA	20
GAPDH_R	TTCACACCCATGACGAACAT	20

Supplementary Table 2. IP-MS proteins list. List of checked protein groups for mass spectrometry-based proteomics of A3C-interacting proteins. Peptide identification was searched with Proteome Discoverer 1.4 software (Thermo Scientific) against Uniprot human complete proteome database (Proteome ID: UP000005640).

**Column description**	
**Protein**	**Explanation**
Accession	Corresponds to the Entry number of the protein in the Uniprot or NCBI database
Description	Protein name
Gene Symbol	Protein coding genes
Sum PEP Score	Scores for all peptides matched to this protein (calculated as the negative log of the posterior error probability).
Coverage	Amino acid coverage of the identified peptides as a percentage of the whole protein.
# PSMs	Number of peptide-spectrum matches (PSMs) identified for this protein.
# Peptides	Number of distinct peptide sequences identified for this protein.
# unique Peptides	Number of peptides identified that are specific to this protein.
# AAs	Length of the amino acid sequence of this protein.
MW[kD]	Protein molecular weight in kilodaltons.
calc. pI	Calculated protein isoelectric point.
**Protein Mass Spectrometry Sequencing Result Filtering Criteria**	
**Peptide:**	Peptide Confidence: High<br>Protein Grouping Options:<br>Enable protein grouping: True<br>Consider leucine and isoleucine as equal: True<br>Consider only PSMs with confidence at least: Medium<br>Consider only PSMs with delta Cn better than: 0.15<br>Apply strict maximum parsimony principle: True
**FDR:**	Target FDR (Strict): 0.01<br>Target FDR (Relaxed): 0.05<br>Validation based on: q-Value

Protein list for control

No.1	Accession	Description	Gene Symbol	Sum PEP Score	Coverage [%]	# Peptides	# PSMs	# Unique Peptides	# AAs	MW [kDa]	calc. pI
#1	P04264	Keratin, type II cytoskeletal 1 OS=Homo sapiens OX=9606 GN=KRT1 PE=1 SV=6	KRT1	98.281	49	30	68	25	644	66	8.12
#2	P13645	Keratin, type I cytoskeletal 10 OS=Homo sapiens OX=9606 GN=KRT10 PE=1 SV=6	KRT10	89.339	42	27	75	21	584	58.8	5.21
#3	P35908	Keratin, type II cytoskeletal 2 epidermal OS=Homo sapiens OX=9606 GN=KRT2 PE=1 SV=2	KRT2	67.583	39	28	50	20	639	65.4	8
#4	P25705	ATP synthase subunit alpha, mitochondrial OS=Homo sapiens OX=9606 GN=ATP5F1A PE=1 SV=1	ATP5A 1	64.559	29	14	49	14	553	59.7	9.13
#5	P19338	Nucleolin OS=Homo sapiens OX=9606 GN=NCL PE=1 SV=3	NCL	64.27	29	24	60	24	710	76.6	4.7
#6	P21796	Voltage-dependent anion-selective channel protein 1 OS=Homo sapiens OX=9606 GN=VDAC1 PE=1 SV=2	VDAC1	62.36	55	12	25	12	283	30.8	8.54
#7	P06576	ATP synthase subunit beta, mitochondrial OS=Homo sapiens OX=9606 GN=ATP5F1B PE=1 SV=3	ATP5B	60.444	31	11	28	11	529	56.5	5.4
#8	P67809	Y-box-binding protein 1 OS=Homo sapiens OX=9606 GN=YBX1 PE=1 SV=3	YBX1	52.363	55	12	24	7	324	35.9	9.88

#9	P05141	ADP/ATP translocase 2 OS=Homo sapiens OX=9606 GN=SLC25A5 PE=1 SV=7	SLC25A5	49.142	29	11	34	11	298	32.8	9.69
#10	P35527	Keratin, type I cytoskeletal 9 OS=Homo sapiens OX=9606 GN=KRT9 PE=1 SV=3	KRT9	49.005	35	18	40	17	623	62	5.24
#11	P36578	60S ribosomal protein L4 OS=Homo sapiens OX=9606 GN=RPL4 PE=1 SV=5	RPL4	43.647	28	12	22	12	427	47.7	11.06
#12	P05387	60S acidic ribosomal protein P2 OS=Homo sapiens OX=9606 GN=RPLP2 PE=1 SV=1	RPLP2	41.696	81	8	18	8	115	11.7	4.54
#13	P52732	Kinesin-like protein KIF11 OS=Homo sapiens OX=9606 GN=KIF11 PE=1 SV=2	KIF11	41.296	20	19	24	19	1056	119.1	5.64
#14	P09651	Heterogeneous nuclear ribonucleoprotein A1 OS=Homo sapiens OX=9606 GN=HNRNPA1 PE=1 SV=5	HNRNPA1	40.693	30	12	26	9	372	38.7	9.13
#15	Q00839	Heterogeneous nuclear ribonucleoprotein U OS=Homo sapiens OX=9606 GN=HNRNPU PE=1 SV=6	HNRNPU	36.941	18	16	35	16	825	90.5	6
#16	P22626	Heterogeneous nuclear ribonucleoproteins A2/B1 OS=Homo sapiens OX=9606 GN=HNRNPA2B1 PE=1 SV=2	HNRNPA2B1	31.543	28	10	17	7	353	37.4	8.95
#17	P62906	60S ribosomal protein L10a OS=Homo sapiens OX=9606 GN=RPL10A PE=1 SV=2	RPL10A	31.112	36	9	15	9	217	24.8	9.94
#18	P13647	Keratin, type II cytoskeletal 5 OS=Homo sapiens OX=9606 GN=KRT5 PE=1 SV=3	KRT5	29.778	21	14	21	7	590	62.3	7.74
#19	P39023	60S ribosomal protein L3 OS=Homo sapiens OX=9606 GN=RPL3 PE=1 SV=2	RPL3	29.29	26	12	19	12	403	46.1	10.18

#20	P05023	Sodium/potassium-transporting ATPase subunit alpha-1 OS=Homo sapiens OX=9606 GN=ATP1A1 PE=1 SV=1	ATP1A 1	29.238	9	7	10	7	1023	112.8	5.49
#21	Q9NRW3	DNA dC->dU-editing enzyme APOBEC-3C OS=Homo sapiens OX=9606 GN=APOBEC3C PE=1 SV=2	APOBE C3C	28.886	36	5	9	4	190	22.8	7.59
#22	P62701	40S ribosomal protein S4, X isoform OS=Homo sapiens OX=9606 GN=RPS4X PE=1 SV=2	RPS4X	27.823	39	11	29	11	263	29.6	10.15
#23	P62424	60S ribosomal protein L7a OS=Homo sapiens OX=9606 GN=RPL7A PE=1 SV=2	RPL7A	27.759	33	9	14	9	266	30	10.61
#24	P16989	Y-box-binding protein 3 OS=Homo sapiens OX=9606 GN=YBX3 PE=1 SV=4	CSDA; YBX3	26.897	17	7	13	2	372	40.1	9.77
#25	P16403	Histone H1.2 OS=Homo sapiens OX=9606 GN=H1-2 PE=1 SV=2	HIST1H 1C	25.087	28	7	14	2	213	21.4	10.93
#26	P11940	Polyadenylate-binding protein 1 OS=Homo sapiens OX=9606 GN=PABPC1 PE=1 SV=2	PABPC 1	25.041	18	11	19	9	636	70.6	9.5
#27	O95831	Apoptosis-inducing factor 1, mitochondrial OS=Homo sapiens OX=9606 GN=AIFM1 PE=1 SV=1	AIFM1	24.482	10	6	6	6	613	66.9	8.95
#28	Q02878	60S ribosomal protein L6 OS=Homo sapiens OX=9606 GN=RPL6 PE=1 SV=3	RPL6	24.078	32	9	21	9	288	32.7	10.58
#29	P05388	60S acidic ribosomal protein P0 OS=Homo sapiens OX=9606 GN=RPLP0 PE=1 SV=1	RPLP0	24.002	25	7	14	7	317	34.3	5.97
#30	P02533	Keratin, type I cytoskeletal 14 OS=Homo sapiens OX=9606 GN=KRT14 PE=1 SV=4	KRT14	23.254	20	11	19	3	472	51.5	5.16

#31	P04259	Keratin, type II cytoskeletal 6B OS=Homo sapiens OX=9606 GN=KRT6B PE=1 SV=5	KRT6B	21.849	15	11	17	2	564	60	8
#32	P30050	60S ribosomal protein L12 OS=Homo sapiens OX=9606 GN=RPL12 PE=1 SV=1	RPL12	21.418	46	6	7	6	165	17.8	9.42
#33	A2NJV5	Immunoglobulin kappa variable 2-29 OS=Homo sapiens OX=9606 GN=IGKV2-29 PE=3 SV=2	IGKV2-29	21.115	19	4	35	3	120	13.1	7.28
#34	P62750	60S ribosomal protein L23a OS=Homo sapiens OX=9606 GN=RPL23A PE=1 SV=1	RPL23A	21.102	38	7	9	7	156	17.7	10.45
#35	P06748	Nucleophosmin OS=Homo sapiens OX=9606 GN=NPM1 PE=1 SV=2	NPM1	20.99	23	7	15	7	294	32.6	4.78
#36	P10412	Histone H1.4 OS=Homo sapiens OX=9606 GN=H1-4 PE=1 SV=2	HIST1H1E	20.888	23	6	13	1	219	21.9	11.03
#37	P38159	RNA-binding motif protein, X chromosome OS=Homo sapiens OX=9606 GN=RBMX PE=1 SV=3	RBMX	19.936	20	8	18	8	391	42.3	10.05
#38	P18124	60S ribosomal protein L7 OS=Homo sapiens OX=9606 GN=RPL7 PE=1 SV=1	RPL7	18.933	34	8	14	8	248	29.2	10.65
#39	Q07020	60S ribosomal protein L18 OS=Homo sapiens OX=9606 GN=RPL18 PE=1 SV=2	RPL18	18.845	42	7	11	7	188	21.6	11.72
#40	P17844	Probable ATP-dependent RNA helicase DDX5 OS=Homo sapiens OX=9606 GN=DDX5 PE=1 SV=1	DDX5	18.407	16	9	13	5	614	69.1	8.92

#41	O60506	Heterogeneous nuclear ribonucleoprotein Q OS=Homo sapiens OX=9606 GN=SYNCRIP PE=1 SV=2	SYNCRIP	18.382	17	9	9	4	623	69.6	8.59
#42	P18621	60S ribosomal protein L17 OS=Homo sapiens OX=9606 GN=RPL17 PE=1 SV=3	RPL17	17.983	27	5	18	5	184	21.4	10.17
#43	P08779	Keratin, type I cytoskeletal 16 OS=Homo sapiens OX=9606 GN=KRT16 PE=1 SV=4	KRT16	17.982	14	9	16	1	473	51.2	5.05
#44	P62241	40S ribosomal protein S8 OS=Homo sapiens OX=9606 GN=RPS8 PE=1 SV=2	RPS8	17.717	38	7	10	7	208	24.2	10.32
#45	P26373	60S ribosomal protein L13 OS=Homo sapiens OX=9606 GN=RPL13 PE=1 SV=4	RPL13	16.987	33	8	12	8	211	24.2	11.65
#46	Q12906	Interleukin enhancer-binding factor 3 OS=Homo sapiens OX=9606 GN=ILF3 PE=1 SV=3	ILF3	16.407	10	7	10	6	894	95.3	8.76
#47	Q9NZI8	Insulin-like growth factor 2 mRNA-binding protein 1 OS=Homo sapiens OX=9606 GN=IGF2BP1 PE=1 SV=2	IGF2BP1	16.323	18	8	12	8	577	63.4	9.2
#48	P13646	Keratin, type I cytoskeletal 13 OS=Homo sapiens OX=9606 GN=KRT13 PE=1 SV=4	KRT13	16.315	11	7	10	1	458	49.6	4.96
#49	P61313	60S ribosomal protein L15 OS=Homo sapiens OX=9606 GN=RPL15 PE=1 SV=2	RPL15	15.978	29	7	12	7	204	24.1	11.62
#50	P83731	60S ribosomal protein L24 OS=Homo sapiens OX=9606 GN=RPL24 PE=1 SV=1	RPL24	15.857	36	7	15	7	157	17.8	11.25
#51	P68104	Elongation factor 1-alpha 1 OS=Homo sapiens OX=9606 GN=EEF1A1 PE=1 SV=1	EEF1A1	15.825	18	7	12	7	462	50.1	9.01

#52	P63173	60S ribosomal protein L38 OS=Homo sapiens OX=9606 GN=RPL38 PE=1 SV=2	RPL38	15.748	36	4	6	4	70	8.2	10.1
#53	P62979	Ubiquitin-40S ribosomal protein S27a OS=Homo sapiens OX=9606 GN=RPS27A PE=1 SV=2	RPS27 A	15.74	42	5	8	2	156	18	9.64
#54	P62269	40S ribosomal protein S18 OS=Homo sapiens OX=9606 GN=RPS18 PE=1 SV=3	RPS18	15.688	43	8	14	8	152	17.7	10.99
#55	Q86V81	THO complex subunit 4 OS=Homo sapiens OX=9606 GN=ALYREF PE=1 SV=3	ALYRE F	15.654	14	3	4	3	257	26.9	11.15
#56	P62917	60S ribosomal protein L8 OS=Homo sapiens OX=9606 GN=RPL8 PE=1 SV=2	RPL8	15.614	27	6	14	6	257	28	11.03
#57	P60709	Actin, cytoplasmic 1 OS=Homo sapiens OX=9606 GN=ACTB PE=1 SV=1	ACTB	15.535	14	4	6	4	375	41.7	5.48
#58	P16401	Histone H1.5 OS=Homo sapiens OX=9606 GN=H1-5 PE=1 SV=3	HIST1H 1B	15.481	15	5	8	5	226	22.6	10.92
#59	P10809	60 kDa heat shock protein, mitochondrial OS=Homo sapiens OX=9606 GN=HSPD1 PE=1 SV=2	HSPD1	15.421	11	5	9	5	573	61	5.87
#60	P61353	60S ribosomal protein L27 OS=Homo sapiens OX=9606 GN=RPL27 PE=1 SV=2	RPL27	15.27	40	6	12	6	136	15.8	10.56
#61	P11142	Heat shock cognate 71 kDa protein OS=Homo sapiens OX=9606 GN=HSPA8 PE=1 SV=1	HSPA8	15.087	13	6	7	6	646	70.9	5.52
#62	P51991	Heterogeneous nuclear ribonucleoprotein A3 OS=Homo sapiens OX=9606 GN=HNRNPA3 PE=1 SV=2	HNRNP A3	14.936	12	5	10	4	378	39.6	9.01

#63	P23396	40S ribosomal protein S3 OS=Homo sapiens OX=9606 GN=RPS3 PE=1 SV=2	RPS3	14.556	28	6	13	6	243	26.7	9.66
#64	P62280	40S ribosomal protein S11 OS=Homo sapiens OX=9606 GN=RPS11 PE=1 SV=3	RPS11	14.35	38	6	19	6	158	18.4	10.3
#65	P40429	60S ribosomal protein L13a OS=Homo sapiens OX=9606 GN=RPL13A PE=1 SV=2	RPL13A	13.795	26	5	9	5	203	23.6	10.93
#66	Q99729	Heterogeneous nuclear ribonucleoprotein A/B OS=Homo sapiens OX=9606 GN=HNRNPAB PE=1 SV=2	HNRNPAB	13.686	14	5	10	4	332	36.2	8.21
#67	P39019	40S ribosomal protein S19 OS=Homo sapiens OX=9606 GN=RPS19 PE=1 SV=2	RPS19	13.465	44	7	9	7	145	16.1	10.32
#68	P62913	60S ribosomal protein L11 OS=Homo sapiens OX=9606 GN=RPL11 PE=1 SV=2	RPL11	13.346	30	4	6	4	178	20.2	9.6
#69	P62249	40S ribosomal protein S16 OS=Homo sapiens OX=9606 GN=RPS16 PE=1 SV=2	RPS16	13.294	36	6	6	6	146	16.4	10.21
#70	Q08211	ATP-dependent RNA helicase A OS=Homo sapiens OX=9606 GN=DHX9 PE=1 SV=4	DHX9	13.275	5	6	8	6	1270	140.9	6.84
#71	P45880	Voltage-dependent anion-selective channel protein 2 OS=Homo sapiens OX=9606 GN=VDAC2 PE=1 SV=2	VDAC2	13.123	13	2	4	2	294	31.5	7.56
#72	P38606	V-type proton ATPase catalytic subunit A OS=Homo sapiens OX=9606 GN=ATP6V1A PE=1 SV=2	ATP6V1A	12.946	5	2	3	2	617	68.3	5.52
#73	P61513	60S ribosomal protein L37a OS=Homo sapiens OX=9606 GN=RPL37A PE=1 SV=2	RPL37A	12.81	38	4	7	4	92	10.3	10.43

#74	P62851	40S ribosomal protein S25 OS=Homo sapiens OX=9606 GN=RPS25 PE=1 SV=1	RPS25	12.646	30	5	9	5	125	13.7	10.11
#75	P15880	40S ribosomal protein S2 OS=Homo sapiens OX=9606 GN=RPS2 PE=1 SV=2	RPS2	12.504	23	7	13	7	293	31.3	10.24
#76	P62244	40S ribosomal protein S15a OS=Homo sapiens OX=9606 GN=RPS15A PE=1 SV=2	RPS15 A	12.165	30	4	19	4	130	14.8	10.13
#77	P31943	Heterogeneous nuclear ribonucleoprotein H OS=Homo sapiens OX=9606 GN=HNRNPH1 PE=1 SV=4	HNRNP H1	12.135	11	4	9	4	449	49.2	6.3
#78	Q13151	Heterogeneous nuclear ribonucleoprotein A0 OS=Homo sapiens OX=9606 GN=HNRNPA0 PE=1 SV=1	HNRNP A0	11.996	21	4	6	4	305	30.8	9.29
#79	P62888	60S ribosomal protein L30 OS=Homo sapiens OX=9606 GN=RPL30 PE=1 SV=2	RPL30	11.939	38	4	7	4	115	12.8	9.63
#80	Q71U36	Tubulin alpha-1A chain OS=Homo sapiens OX=9606 GN=TUBA1A PE=1 SV=1	TUBA1 A	11.358	17	5	5	5	451	50.1	5.06
#81	P62910	60S ribosomal protein L32 OS=Homo sapiens OX=9606 GN=RPL32 PE=1 SV=2	RPL32	11.297	27	4	7	4	135	15.9	11.33
#82	P62899	60S ribosomal protein L31 OS=Homo sapiens OX=9606 GN=RPL31 PE=1 SV=1	RPL31	11.273	30	4	16	4	125	14.5	10.54
#83	Q13813	Spectrin alpha chain, non-erythrocytic 1 OS=Homo sapiens OX=9606 GN=SPTAN1 PE=1 SV=3	SPTAN 1	11.269	2	3	3	3	2472	284.4	5.35

#84	Q9UJS0	Calcium-binding mitochondrial carrier protein Aralar2 OS=Homo sapiens OX=9606 GN=SLC25A13 PE=1 SV=2	SLC25 A13	11.205	4	3	4	3	675	74.1	8.62
#85	P62753	40S ribosomal protein S6 OS=Homo sapiens OX=9606 GN=RPS6 PE=1 SV=1	RPS6	11.187	17	4	7	4	249	28.7	10.84
#86	P28331	NADH-ubiquinone oxidoreductase 75 kDa subunit, mitochondrial OS=Homo sapiens OX=9606 GN=NDUFS1 PE=1 SV=3	NDUFS 1	11.142	5	2	6	2	727	79.4	6.23
#87	P02768	Albumin OS=Homo sapiens OX=9606 GN=ALB PE=1 SV=2	ALB	11.124	9	5	11	5	609	69.3	6.28
#88	P61254	60S ribosomal protein L26 OS=Homo sapiens OX=9606 GN=RPL26 PE=1 SV=1	RPL26	11.079	30	7	13	7	145	17.2	10.55
#89	Q99623	Prohibitin-2 OS=Homo sapiens OX=9606 GN=PHB2 PE=1 SV=2	PHB2	11.032	10	2	3	2	299	33.3	9.83
#90	P62266	40S ribosomal protein S23 OS=Homo sapiens OX=9606 GN=RPS23 PE=1 SV=3	RPS23	10.994	29	5	8	5	143	15.8	10.49
#91	O00571	ATP-dependent RNA helicase DDX3X OS=Homo sapiens OX=9606 GN=DDX3X PE=1 SV=3	DDX3X	10.985	11	6	7	6	662	73.2	7.18
#92	P61247	40S ribosomal protein S3a OS=Homo sapiens OX=9606 GN=RPS3A PE=1 SV=2	RPS3A	10.948	20	7	12	7	264	29.9	9.73
#93	P62277	40S ribosomal protein S13 OS=Homo sapiens OX=9606 GN=RPS13 PE=1 SV=2	RPS13	10.753	26	5	8	5	151	17.2	10.54
#94	O00622	CCN family member 1 OS=Homo sapiens OX=9606 GN=CCN1 PE=1 SV=1	CYR61	10.499	14	6	8	6	381	42	8.21

#95	Q9NX63	MICOS complex subunit MIC19 OS=Homo sapiens OX=9606 GN=CHCHD3 PE=1 SV=1	CHCHD3	10.483	7	2	3	2	227	26.1	8.28
#96	O43390	Heterogeneous nuclear ribonucleoprotein R OS=Homo sapiens OX=9606 GN=HNRNPR PE=1 SV=1	HNRNPR	10.466	11	7	7	2	633	70.9	8.13
#97	Q9Y277	Voltage-dependent anion-selective channel protein 3 OS=Homo sapiens OX=9606 GN=VDAC3 PE=1 SV=1	VDAC3	10.298	14	2	4	2	283	30.6	8.66
#98	P06733	Alpha-enolase OS=Homo sapiens OX=9606 GN=ENO1 PE=1 SV=2	ENO1	10.047	5	2	3	2	434	47.1	7.39
#99	Q92841	Probable ATP-dependent RNA helicase DDX17 OS=Homo sapiens OX=9606 GN=DDX17 PE=1 SV=2	DDX17	9.993	8	5	8	1	729	80.2	8.27
#100	Q5QNW6	Histone H2B type 2-F OS=Homo sapiens OX=9606 GN=H2BC18 PE=1 SV=3	HIST2H2BF	9.913	21	4	4	4	126	13.9	10.32
#101	P61978	Heterogeneous nuclear ribonucleoprotein K OS=Homo sapiens OX=9606 GN=HNRNPK PE=1 SV=1	HNRNPK	9.885	12	5	7	5	463	50.9	5.54
#102	Q13310	Polyadenylate-binding protein 4 OS=Homo sapiens OX=9606 GN=PABPC4 PE=1 SV=1	PABPC4	9.693	10	5	6	3	644	70.7	9.26
#103	P69905	Hemoglobin subunit alpha OS=Homo sapiens OX=9606 GN=HBA1 PE=1 SV=2	HBA2; HBA1	9.657	25	4	7	4	142	15.2	8.68
#104	P62987	Ubiquitin-60S ribosomal protein L40 OS=Homo sapiens OX=9606 GN=UBA52 PE=1 SV=2	UBA52	9.474	34	4	6	1	128	14.7	9.83

#105	Q06830	Peroxiredoxin-1 OS=Homo sapiens OX=9606 GN=PRDX1 PE=1 SV=1	PRDX1	9.472	20	5	5	5	199	22.1	8.13
#106	Q02543	60S ribosomal protein L18a OS=Homo sapiens OX=9606 GN=RPL18A PE=1 SV=2	RPL18 A	9.413	21	4	6	4	176	20.7	10.71
#107	P50914	60S ribosomal protein L14 OS=Homo sapiens OX=9606 GN=RPL14 PE=1 SV=4	RPL14	9.175	14	3	7	3	215	23.4	10.93
#108	O95881	Thioredoxin domain-containing protein 12 OS=Homo sapiens OX=9606 GN=TXNDC12 PE=1 SV=1	TXNDC 12	8.934	19	3	7	3	172	19.2	5.4
#109	P62841	40S ribosomal protein S15 OS=Homo sapiens OX=9606 GN=RPS15 PE=1 SV=2	RPS15	8.761	14	2	3	2	145	17	10.39
#110	P81605	Dermcidin OS=Homo sapiens OX=9606 GN=DCD PE=1 SV=2	DCD	8.425	23	3	4	3	110	11.3	6.54
#111	P08708	40S ribosomal protein S17 OS=Homo sapiens OX=9606 GN=RPS17 PE=1 SV=2	RPS17; RPS17L	8.264	16	4	4	4	135	15.5	9.85
#112	P62829	60S ribosomal protein L23 OS=Homo sapiens OX=9606 GN=RPL23 PE=1 SV=1	RPL23	8.14	33	4	8	4	140	14.9	10.51
#113	P83881	60S ribosomal protein L36a OS=Homo sapiens OX=9606 GN=RPL36A PE=1 SV=2	RPL36 A	8.059	27	5	12	2	106	12.4	10.58
#114	P46779	60S ribosomal protein L28 OS=Homo sapiens OX=9606 GN=RPL28 PE=1 SV=3	RPL28	8.028	20	4	6	4	137	15.7	12.02
#115	Q92522	Histone H1.10 OS=Homo sapiens OX=9606 GN=H1-10 PE=1 SV=1	H1FX	7.993	17	3	4	3	213	22.5	10.76

#116	P04350	Tubulin beta-4A chain OS=Homo sapiens OX=9606 GN=TUBB4A PE=1 SV=2	TUBB4A	7.897	5	2	2	2	444	49.6	4.88
#117	P42766	60S ribosomal protein L35 OS=Homo sapiens OX=9606 GN=RPL35 PE=1 SV=2	RPL35	7.749	33	4	6	4	123	14.5	11.05
#118	P46781	40S ribosomal protein S9 OS=Homo sapiens OX=9606 GN=RPS9 PE=1 SV=3	RPS9	7.701	15	5	14	5	194	22.6	10.65
#119	P60866	40S ribosomal protein S20 OS=Homo sapiens OX=9606 GN=RPS20 PE=1 SV=1	RPS20	7.684	25	3	8	3	119	13.4	9.94
#120	P47914	60S ribosomal protein L29 OS=Homo sapiens OX=9606 GN=RPL29 PE=1 SV=2	RPL29	7.665	14	2	11	2	159	17.7	11.66
#121	P25398	40S ribosomal protein S12 OS=Homo sapiens OX=9606 GN=RPS12 PE=1 SV=3	RPS12	7.317	25	3	4	3	132	14.5	7.21
#122	P62263	40S ribosomal protein S14 OS=Homo sapiens OX=9606 GN=RPS14 PE=1 SV=3	RPS14	7.245	16	3	5	3	151	16.3	10.05
#123	P19474	E3 ubiquitin-protein ligase TRIM21 OS=Homo sapiens OX=9606 GN=TRIM21 PE=1 SV=1	TRIM21	7.209	7	3	4	3	475	54.1	6.38
#124	Q9BUJ2	Heterogeneous nuclear ribonucleoprotein U-like protein 1 OS=Homo sapiens OX=9606 GN=HNRNPUL1 PE=1 SV=2	HNRNPUL1	7.166	5	4	6	4	856	95.7	6.92
#125	P21281	V-type proton ATPase subunit B, brain isoform OS=Homo sapiens OX=9606 GN=ATP6V1B2 PE=1 SV=3	ATP6V1B2	7.056	6	2	5	2	511	56.5	5.81
#126	O43707	Alpha-actinin-4 OS=Homo sapiens OX=9606 GN=ACTN4 PE=1 SV=2	ACTN4	6.962	3	2	4	2	911	104.8	5.44

#127	Q6PKH6	Dehydrogenase/reductase SDR family member 4-like 2 OS=Homo sapiens OX=9606 GN=DHRS4L2 PE=2 SV=1	DHRS4 L2	6.956	7	1	1	1	230	24.6	10.11
#128	Q16891	MICOS complex subunit MIC60 OS=Homo sapiens OX=9606 GN=IMMT PE=1 SV=1	IMMT	6.939	2	1	7	1	758	83.6	6.48
#129	P52272	Heterogeneous nuclear ribonucleoprotein M OS=Homo sapiens OX=9606 GN=HNRNPM PE=1 SV=3	HNRNP M	6.856	4	2	2	2	730	77.5	8.7
#130	P52815	39S ribosomal protein L12, mitochondrial OS=Homo sapiens OX=9606 GN=MRPL12 PE=1 SV=2	MRPL1 2	6.537	12	2	3	2	198	21.3	8.87
#131	P07237	Protein disulfide-isomerase OS=Homo sapiens OX=9606 GN=P4HB PE=1 SV=3	P4HB	6.379	3	1	1	1	508	57.1	4.87
#132	P51114	Fragile X mental retardation syndrome-related protein 1 OS=Homo sapiens OX=9606 GN=FXR1 PE=1 SV=3	FXR1	6.145	6	3	3	3	621	69.7	6.15
#133	P04843	Dolichyl-diphosphooligosaccharide--protein glycosyltransferase subunit 1 OS=Homo sapiens OX=9606 GN=RPN1 PE=1 SV=1	RPN1	6.062	7	2	2	2	607	68.5	6.38
#134	Q7RTS7	Keratin, type II cytoskeletal 74 OS=Homo sapiens OX=9606 GN=KRT74 PE=1 SV=2	KRT74	6.043	5	4	4	1	529	57.8	7.71
#135	Q969Q0	60S ribosomal protein L36a-like OS=Homo sapiens OX=9606 GN=RPL36AL PE=1 SV=3	RPL36 AL	5.991	27	4	7	1	106	12.5	10.65
#136	P27635	60S ribosomal protein L10 OS=Homo sapiens OX=9606 GN=RPL10 PE=1 SV=4	RPL10	5.92	18	5	5	5	214	24.6	10.08

#137	P46776	60S ribosomal protein L27a OS=Homo sapiens OX=9606 GN=RPL27A PE=1 SV=2	RPL27 A	5.864	21	3	3	3	148	16.6	11
#138	P10599	Thioredoxin OS=Homo sapiens OX=9606 GN=TXN PE=1 SV=3	TXN	5.828	21	2	2	2	105	11.7	4.92
#139	Q9NXV2	BTB/POZ domain-containing protein KCTD5 OS=Homo sapiens OX=9606 GN=KCTD5 PE=1 SV=1	KCTD5	5.822	9	2	3	2	234	26.1	6.24
#140	P49821	NADH dehydrogenase [ubiquinone] flavoprotein 1, mitochondrial OS=Homo sapiens OX=9606 GN=NDUFV1 PE=1 SV=4	NDUFV 1	5.801	3	1	1	1	464	50.8	8.21
#141	P16615	Sarcoplasmic/endoplasmic reticulum calcium ATPase 2 OS=Homo sapiens OX=9606 GN=ATP2A2 PE=1 SV=1	ATP2A 2	5.683	5	3	3	3	1042	114.7	5.34
#142	P46777	60S ribosomal protein L5 OS=Homo sapiens OX=9606 GN=RPL5 PE=1 SV=3	RPL5	5.668	12	4	6	4	297	34.3	9.72
#143	Q00610	Clathrin heavy chain 1 OS=Homo sapiens OX=9606 GN=CLTC PE=1 SV=5	CLTC	5.595	3	3	3	3	1675	191.5	5.69
#144	Q8IUX4	DNA dC->dU-editing enzyme APOBEC-3F OS=Homo sapiens OX=9606 GN=APOBEC3F PE=1 SV=3	APOBE C3F	5.591	8	2	3	1	373	45	7.23
#145	O15400	Syntaxin-7 OS=Homo sapiens OX=9606 GN=STX7 PE=1 SV=4	STX7	5.535	5	1	2	1	261	29.8	5.55
#146	P18077	60S ribosomal protein L35a OS=Homo sapiens OX=9606 GN=RPL35A PE=1 SV=2	RPL35 A	5.48	21	3	7	3	110	12.5	11.06

#147	P31930	Cytochrome b-c1 complex subunit 1, mitochondrial OS=Homo sapiens OX=9606 GN=UQCRC1 PE=1 SV=3	UQCRC1	5.471	4	1	3	1	480	52.6	6.37
#148	P11498	Pyruvate carboxylase, mitochondrial OS=Homo sapiens OX=9606 GN=PC PE=1 SV=2	PC	5.458	2	2	3	2	1178	129.6	6.84
#149	Q14103	Heterogeneous nuclear ribonucleoprotein D0 OS=Homo sapiens OX=9606 GN=HNRNPD PE=1 SV=1	HNRNPD	5.442	8	2	3	2	355	38.4	7.81
#150	P05062	Fructose-bisphosphate aldolase B OS=Homo sapiens OX=9606 GN=ALDOB PE=1 SV=2	ALDOB	5.397	8	2	4	2	364	39.4	7.87
#151	P08670	Vimentin OS=Homo sapiens OX=9606 GN=VIM PE=1 SV=4	VIM	5.396	6	3	4	3	466	53.6	5.12
#152	E9PRG8	Uncharacterized protein C11orf98 OS=Homo sapiens OX=9606 GN=C11orf98 PE=4 SV=2		5.259	17	2	4	2	123	14.2	11.53
#153	P35579	Myosin-9 OS=Homo sapiens OX=9606 GN=MYH9 PE=1 SV=4	MYH9	5.257	1	2	2	2	1960	226.4	5.6
#154	Q8N8Y2	V-type proton ATPase subunit d 2 OS=Homo sapiens OX=9606 GN=ATP6V0D2 PE=1 SV=1	ATP6V0D2	5.122	5	1	1	1	350	40.4	5.3
#155	O95793	Double-stranded RNA-binding protein Staufen homolog 1 OS=Homo sapiens OX=9606 GN=STAU1 PE=1 SV=2	STAU1	5.068	4	2	2	2	577	63.1	9.44
#156	P46778	60S ribosomal protein L21 OS=Homo sapiens OX=9606 GN=RPL21 PE=1 SV=2	RPL21	5.038	9	1	1	1	160	18.6	10.49
#157	Q9UM54	Unconventional myosin-VI OS=Homo sapiens OX=9606 GN=MYO6 PE=1 SV=4	MYO6	5.028	3	2	2	2	1294	149.6	8.53

#158	P48047	ATP synthase subunit O, mitochondrial OS=Homo sapiens OX=9606 GN=ATP5PO PE=1 SV=1	ATP5O	5.013	7	1	1	1	213	23.3	9.96
#159	P21912	Succinate dehydrogenase [ubiquinone] iron-sulfur subunit, mitochondrial OS=Homo sapiens OX=9606 GN=SDHB PE=1 SV=3	SDHB	4.9	4	1	2	1	280	31.6	8.76
#160	P38646	Stress-70 protein, mitochondrial OS=Homo sapiens OX=9606 GN=HSPA9 PE=1 SV=2	HSPA9	4.863	3	1	1	1	679	73.6	6.16
#161	P55072	Transitional endoplasmic reticulum ATPase OS=Homo sapiens OX=9606 GN=VCP PE=1 SV=4	VCP	4.792	2	1	1	1	806	89.3	5.26
#162	P60953	Cell division control protein 42 homolog OS=Homo sapiens OX=9606 GN=CDC42 PE=1 SV=2	CDC42	4.763	11	1	2	1	191	21.2	6.55
#163	Q9UN81	LINE-1 retrotransposable element ORF1 protein OS=Homo sapiens OX=9606 GN=L1RE1 PE=1 SV=1	LOC440264; L1RE1	4.755	7	2	2	2	338	40	9.51
#164	P36957	Dihydrolipoyllysine-residue succinyltransferase component of 2-oxoglutarate dehydrogenase complex, mitochondrial OS=Homo sapiens OX=9606 GN=DLST PE=1 SV=4	DLST	4.696	3	1	1	1	453	48.7	8.95
#165	P49458	Signal recognition particle 9 kDa protein OS=Homo sapiens OX=9606 GN=SRP9 PE=1 SV=2	SRP9; SRP9P1	4.57	23	2	3	2	86	10.1	7.97

#166	P49411	Elongation factor Tu, mitochondrial OS=Homo sapiens OX=9606 GN=TUFM PE=1 SV=2	TUFM	4.507	3	1	1	1	452	49.5	7.61
#167	P62854	40S ribosomal protein S26 OS=Homo sapiens OX=9606 GN=RPS26 PE=1 SV=3	RPS26; LOC101929876; RPS26P25	4.506	18	2	5	2	115	13	11
#168	P62820	Ras-related protein Rab-1A OS=Homo sapiens OX=9606 GN=RAB1A PE=1 SV=3	RAB1A	4.436	10	1	2	1	205	22.7	6.21
#169	P02042	Hemoglobin subunit delta OS=Homo sapiens OX=9606 GN=HBD PE=1 SV=2	HBD	4.394	13	2	4	2	147	16	8.05
#170	P49207	60S ribosomal protein L34 OS=Homo sapiens OX=9606 GN=RPL34 PE=1 SV=3	RPL34	4.342	15	2	6	2	117	13.3	11.47
#171	P53680	AP-2 complex subunit sigma OS=Homo sapiens OX=9606 GN=AP2S1 PE=1 SV=2	AP2S1	4.316	8	1	1	1	142	17	6.18
#172	Q15717	ELAV-like protein 1 OS=Homo sapiens OX=9606 GN=ELAVL1 PE=1 SV=2	ELAVL1	4.296	10	3	3	3	326	36.1	9.17
#173	P10636	Microtubule-associated protein tau OS=Homo sapiens OX=9606 GN=MAPT PE=1 SV=5	MAPT	4.276	1	2	3	2	758	78.9	6.71
#174	P22033	Methylmalonyl-CoA mutase, mitochondrial OS=Homo sapiens OX=9606 GN=MMUT PE=1 SV=4	MUT	4.272	2	1	1	1	750	83.1	6.93

#175	O14975	Very long-chain acyl-CoA synthetase OS=Homo sapiens OX=9606 GN=SLC27A2 PE=1 SV=2	SLC27 A2	4.259	2	1	3	1	620	70.3	8.51
#176	P08865	40S ribosomal protein SA OS=Homo sapiens OX=9606 GN=RPSA PE=1 SV=4	RPSA	4.096	7	2	3	2	295	32.8	4.87
#177	P07900	Heat shock protein HSP 90-alpha OS=Homo sapiens OX=9606 GN=HSP90AA1 PE=1 SV=5	HSP90 AA1	4.077	2	1	1	1	732	84.6	5.02
#178	Q9Y2W1	Thyroid hormone receptor-associated protein 3 OS=Homo sapiens OX=9606 GN=THRAP3 PE=1 SV=2	THRAP 3	4	3	3	4	3	955	108.6	10.15
#179	Q14444	Caprin-1 OS=Homo sapiens OX=9606 GN=CAPRIN1 PE=1 SV=2	CAPRI N1	3.967	2	1	1	1	709	78.3	5.25
#180	P62273	40S ribosomal protein S29 OS=Homo sapiens OX=9606 GN=RPS29 PE=1 SV=2	RPS29	3.897	21	1	5	1	56	6.7	10.13
#181	P48735	Isocitrate dehydrogenase [NADP], mitochondrial OS=Homo sapiens OX=9606 GN=IDH2 PE=1 SV=2	IDH2	3.888	9	2	3	2	452	50.9	8.69
#182	P00167	Cytochrome b5 OS=Homo sapiens OX=9606 GN=CYB5A PE=1 SV=2	CYB5A	3.848	25	1	1	1	134	15.3	4.96
#183	Q8WUD1	Ras-related protein Rab-2B OS=Homo sapiens OX=9606 GN=RAB2B PE=1 SV=1	RAB2B	3.828	7	1	1	1	216	24.2	7.83
#184	O14979	Heterogeneous nuclear ribonucleoprotein D-like OS=Homo sapiens OX=9606 GN=HNRNPDL PE=1 SV=3	HNRNP DL; HNRPD L	3.826	4	2	4	1	420	46.4	9.57

#185	Q13247	Serine/arginine-rich splicing factor 6 OS=Homo sapiens OX=9606 GN=SRSF6 PE=1 SV=2	SRSF6	3.726	7	3	4	3	344	39.6	11.43
#186	Q01740	Dimethylaniline monooxygenase [N-oxide-forming] 1 OS=Homo sapiens OX=9606 GN=FMO1 PE=1 SV=3	FMO1	3.692	3	1	2	1	532	60.3	7.2
#187	P84098	60S ribosomal protein L19 OS=Homo sapiens OX=9606 GN=RPL19 PE=1 SV=1	RPL19	3.69	13	2	3	2	196	23.5	11.47
#188	P49189	4-trimethylaminobutyraldehyde dehydrogenase OS=Homo sapiens OX=9606 GN=ALDH9A1 PE=1 SV=3	ALDH9 A1	3.661	3	1	1	1	494	53.8	5.87
#189	P00367	Glutamate dehydrogenase 1, mitochondrial OS=Homo sapiens OX=9606 GN=GLUD1 PE=1 SV=2	GLUD1	3.643	3	1	1	1	558	61.4	7.8
#190	P62861	40S ribosomal protein S30 OS=Homo sapiens OX=9606 GN=FAU PE=1 SV=1	FAU	3.634	17	1	1	1	59	6.6	12.15
#191	Q9Y490	Talin-1 OS=Homo sapiens OX=9606 GN=TLN1 PE=1 SV=3	TLN1	3.621	0	1	2	1	2541	269.6	6.07
#192	P07305	Histone H1.0 OS=Homo sapiens OX=9606 GN=H1-0 PE=1 SV=3	H1F0	3.611	12	2	2	2	194	20.9	10.84
#193	P07954	Fumarate hydratase, mitochondrial OS=Homo sapiens OX=9606 GN=FH PE=1 SV=3	FH	3.587	4	1	1	1	510	54.6	8.76
#194	P30154	Serine/threonine-protein phosphatase 2A 65 kDa regulatory subunit A beta isoform OS=Homo sapiens OX=9606 GN=PPP2R1B PE=1 SV=3	PPP2R1 B	3.541	2	1	3	1	601	66.2	4.94

#195	A0A075B6R9	Probable non-functional immunoglobulin kappa variable 2D-24 OS=Homo sapiens OX=9606 GN=IGKV2D-24 PE=1 SV=1		3.539	17	2	9	1	120	13.1	8.87
#196	P20290	Transcription factor BTF3 OS=Homo sapiens OX=9606 GN=BTF3 PE=1 SV=1	BTF3	3.533	9	1	1	1	206	22.2	9.38
#197	P40227	T-complex protein 1 subunit zeta OS=Homo sapiens OX=9606 GN=CCT6A PE=1 SV=3	CCT6A	3.507	2	1	1	1	531	58	6.68
#198	Q86U42	Polyadenylate-binding protein 2 OS=Homo sapiens OX=9606 GN=PABPN1 PE=1 SV=3	PABPN1	3.413	6	1	1	1	306	32.7	5.06
#199	P05186	Alkaline phosphatase, tissue-nonspecific isozyme OS=Homo sapiens OX=9606 GN=ALPL PE=1 SV=4	ALPL	3.408	3	1	1	1	524	57.3	6.67
#200	P39656	Dolichyl-diphosphooligosaccharide--protein glycosyltransferase 48 kDa subunit OS=Homo sapiens OX=9606 GN=DDOST PE=1 SV=4	DDOST	3.406	3	1	1	1	456	50.8	6.55
#201	Q13501	Sequestosome-1 OS=Homo sapiens OX=9606 GN=SQSTM1 PE=1 SV=1	SQSTM1	3.401	6	1	1	1	440	47.7	5.22
#202	P50213	Isocitrate dehydrogenase [NAD] subunit alpha, mitochondrial OS=Homo sapiens OX=9606 GN=IDH3A PE=1 SV=1	IDH3A	3.297	3	1	3	1	366	39.6	6.92
#203	P21741	Midkine OS=Homo sapiens OX=9606 GN=MDK PE=1 SV=1	MDK	3.249	7	1	1	1	143	15.6	9.79
#204	Q15363	Transmembrane emp24 domain-containing protein 2 OS=Homo sapiens OX=9606 GN=TMED2 PE=1 SV=1	TMED2	3.243	6	1	1	1	201	22.7	5.17

#205	P40926	Malate dehydrogenase, mitochondrial OS=Homo sapiens OX=9606 GN=MDH2 PE=1 SV=3	MDH2	3.214	5	1	1	1	338	35.5	8.68
#206	P99999	Cytochrome c OS=Homo sapiens OX=9606 GN=CYCS PE=1 SV=2	CYCS	3.214	12	2	2	2	105	11.7	9.57
#207	Q3KQZ1	Solute carrier family 25 member 35 OS=Homo sapiens OX=9606 GN=SLC25A35 PE=1 SV=1	SLC25 A35	3.197	6	1	1	1	300	32.4	9.11
#208	Q00325	Phosphate carrier protein, mitochondrial OS=Homo sapiens OX=9606 GN=SLC25A3 PE=1 SV=2	SLC25 A3	3.159	4	1	1	1	362	40.1	9.38
#209	P06753	Tropomyosin alpha-3 chain OS=Homo sapiens OX=9606 GN=TPM3 PE=1 SV=2	TPM3	3.068	5	1	2	1	285	32.9	4.72
#210	Q96SI9	Spermatid perinuclear RNA-binding protein OS=Homo sapiens OX=9606 GN=STRBP PE=1 SV=1	STRBP	3.021	3	2	2	1	672	73.6	8.72
#211	P35268	60S ribosomal protein L22 OS=Homo sapiens OX=9606 GN=RPL22 PE=1 SV=2	RPL22	2.987	10	1	1	1	128	14.8	9.19
#212	O00483	Cytochrome c oxidase subunit NDUFA4 OS=Homo sapiens OX=9606 GN=NDUFA4 PE=1 SV=1	NDUFA 4	2.965	25	2	2	2	81	9.4	9.38
#213	Q9BRJ6	Uncharacterized protein C7orf50 OS=Homo sapiens OX=9606 GN=C7orf50 PE=1 SV=1	C7orf50	2.894	11	1	2	1	194	22.1	9.64
#214	P01859	Immunoglobulin heavy constant gamma 2 OS=Homo sapiens OX=9606 GN=IGHG2 PE=1 SV=2	IGHG2	2.858	3	1	3	1	326	35.9	7.59

#215	Q9Y512	Sorting and assembly machinery component 50 homolog OS=Homo sapiens OX=9606 GN=SAMM50 PE=1 SV=3	SAMM 50	2.849	3	1	1	1	469	51.9	6.9
#216	Q92804	TATA-binding protein-associated factor 2N OS=Homo sapiens OX=9606 GN=TAF15 PE=1 SV=1	TAF15	2.837	8	2	2	2	592	61.8	8.02
#217	Q6PKG0	La-related protein 1 OS=Homo sapiens OX=9606 GN=LARP1 PE=1 SV=2	LARP1	2.799	2	2	2	2	1096	123.4	8.82
#218	Q14011	Cold-inducible RNA-binding protein OS=Homo sapiens OX=9606 GN=CIRBP PE=1 SV=1	CIRBP	2.795	6	1	1	1	172	18.6	9.51
#219	A8MXV4	Nucleoside diphosphate-linked moiety X motif 19 OS=Homo sapiens OX=9606 GN=NUDT19 PE=1 SV=1	NUDT1 9	2.773	4	1	1	1	375	42.2	7.64
#220	P62318	Small nuclear ribonucleoprotein Sm D3 OS=Homo sapiens OX=9606 GN=SNRPD3 PE=1 SV=1	SNRPD 3	2.771	8	1	1	1	126	13.9	10.32
#221	P41091	Eukaryotic translation initiation factor 2 subunit 3 OS=Homo sapiens OX=9606 GN=EIF2S3 PE=1 SV=3	EIF2S3	2.685	4	1	1	1	472	51.1	8.4
#222	P00505	Aspartate aminotransferase, mitochondrial OS=Homo sapiens OX=9606 GN=GOT2 PE=1 SV=3	GOT2	2.664	3	1	1	1	430	47.5	9.01
#223	P63244	Receptor of activated protein C kinase 1 OS=Homo sapiens OX=9606 GN=RACK1 PE=1 SV=3	GNB2L 1; RACK1	2.663	5	1	1	1	317	35.1	7.69

#224	P35232	Prohibitin OS=Homo sapiens OX=9606 GN=PHB PE=1 SV=1	PHB	2.617	4	1	2	1	272	29.8	5.76
#225	P32189	Glycerol kinase OS=Homo sapiens OX=9606 GN=GK PE=1 SV=3	GK	2.553	2	1	1	1	559	61.2	6.54
#226	Q14498	RNA-binding protein 39 OS=Homo sapiens OX=9606 GN=RBM39 PE=1 SV=2	RBM39	2.528	4	2	2	2	530	59.3	10.1
#227	Q7Z406	Myosin-14 OS=Homo sapiens OX=9606 GN=MYH14 PE=1 SV=2	MYH14	2.494	1	1	1	1	1995	227.7	5.6
#228	Q00577	Transcriptional activator protein Pur-alpha OS=Homo sapiens OX=9606 GN=PURA PE=1 SV=2	PURA	2.424	6	2	3	2	322	34.9	6.44
#229	Q9BRL6	Serine/arginine-rich splicing factor 8 OS=Homo sapiens OX=9606 GN=SRSF8 PE=1 SV=1	SRSF8	2.409	3	1	2	1	282	32.3	11.72
#230	P53999	Activated RNA polymerase II transcriptional coactivator p15 OS=Homo sapiens OX=9606 GN=SUB1 PE=1 SV=3	SUB1	2.295	9	1	1	1	127	14.4	9.6
#231	Q13242	Serine/arginine-rich splicing factor 9 OS=Homo sapiens OX=9606 GN=SRSF9 PE=1 SV=1	SRSF9	2.292	5	1	1	1	221	25.5	8.65
#232	P62995	Transformer-2 protein homolog beta OS=Homo sapiens OX=9606 GN=TRA2B PE=1 SV=1	TRA2B	2.19	6	1	1	1	288	33.6	11.25
#233	P83111	Serine beta-lactamase-like protein LACTB, mitochondrial OS=Homo sapiens OX=9606 GN=LACTB PE=1 SV=2	LACTB	2.186	3	1	1	1	547	60.7	8.53

#234	Q9HC36	rRNA methyltransferase 3, mitochondrial OS=Homo sapiens OX=9606 GN=MRM3 PE=1 SV=2	RNMTL1; MRM3	2.137	3	1	1	1	420	47	8.73
#235	P63220	40S ribosomal protein S21 OS=Homo sapiens OX=9606 GN=RPS21 PE=1 SV=1	RPS21	2.107	11	1	2	1	83	9.1	8.5
#236	P62316	Small nuclear ribonucleoprotein Sm D2 OS=Homo sapiens OX=9606 GN=SNRPD2 PE=1 SV=1	SNRPD2	2.106	8	1	1	1	118	13.5	9.91
#237	O60313	Dynamin-like 120 kDa protein, mitochondrial OS=Homo sapiens OX=9606 GN=OPA1 PE=1 SV=3	OPA1	2.097	3	1	1	1	960	111.6	7.87
#238	P61927	60S ribosomal protein L37 OS=Homo sapiens OX=9606 GN=RPL37 PE=1 SV=2	RPL37	2.078	10	1	2	1	97	11.1	11.74
#239	Q16777	Histone H2A type 2-C OS=Homo sapiens OX=9606 GN=H2AC20 PE=1 SV=4	HIST2H2AC	1.991	12	2	2	2	129	14	10.9
#240	Q71UM5	40S ribosomal protein S27-like OS=Homo sapiens OX=9606 GN=RPS27L PE=1 SV=3	RPS27L	1.968	10	1	1	1	84	9.5	9.45
#241	P37108	Signal recognition particle 14 kDa protein OS=Homo sapiens OX=9606 GN=SRP14 PE=1 SV=2	SRP14	1.956	7	2	2	2	136	14.6	10.04
#242	Q9NX58	Cell growth-regulating nucleolar protein OS=Homo sapiens OX=9606 GN=LYAR PE=1 SV=2	LYAR	1.919	4	1	1	1	379	43.6	9.54
#243	P20962	Parathymosin OS=Homo sapiens OX=9606 GN=PTMS PE=1 SV=2	PTMS	1.89	11	1	1	1	102	11.5	4.16

#244	P55084	Trifunctional enzyme subunit beta, mitochondrial OS=Homo sapiens OX=9606 GN=HADHB PE=1 SV=3	HADHB	1.887	2	1	1	1	474	51.3	9.41
#245	P84103	Serine/arginine-rich splicing factor 3 OS=Homo sapiens OX=9606 GN=SRSF3 PE=1 SV=1	SRSF3	1.833	5	1	1	1	164	19.3	11.65
#246	P17987	T-complex protein 1 subunit alpha OS=Homo sapiens OX=9606 GN=TCP1 PE=1 SV=1	TCP1	1.815	2	1	1	1	556	60.3	6.11
#247	P28288	ATP-binding cassette sub-family D member 3 OS=Homo sapiens OX=9606 GN=ABCD3 PE=1 SV=1	ABCD3	1.785	2	1	1	1	659	75.4	9.36
#248	Q562R1	Beta-actin-like protein 2 OS=Homo sapiens OX=9606 GN=ACTBL2 PE=1 SV=2	ACTBL2	1.771	5	1	1	1	376	42	5.59
#249	P13639	Elongation factor 2 OS=Homo sapiens OX=9606 GN=EEF2 PE=1 SV=4	EEF2	1.734	1	1	1	1	858	95.3	6.83
#250	P01857	Immunoglobulin heavy constant gamma 1 OS=Homo sapiens OX=9606 GN=IGHG1 PE=1 SV=1	IGHG1	1.721	5	1	2	1	330	36.1	8.19
#251	O75251	NADH dehydrogenase [ubiquinone] iron-sulfur protein 7, mitochondrial OS=Homo sapiens OX=9606 GN=NDUFS7 PE=1 SV=3	NDUFS7	1.614	7	1	1	1	213	23.5	9.99
#252	P09012	U1 small nuclear ribonucleoprotein A OS=Homo sapiens OX=9606 GN=SNRPA PE=1 SV=3	SNRPA	1.592	4	1	1	1	282	31.3	9.83

#253	Q9Y383	Putative RNA-binding protein Luc7-like 2 OS=Homo sapiens OX=9606 GN=LUC7L2 PE=1 SV=2	LUC7L2	1.53	4	1	1	1	392	46.5	10.01
#254	P14174	Macrophage migration inhibitory factor OS=Homo sapiens OX=9606 GN=MIF PE=1 SV=4	MIF	1.494	10	1	2	1	115	12.5	7.88
#255	Q9GZT6	Coiled-coil domain-containing protein 90B, mitochondrial OS=Homo sapiens OX=9606 GN=CCDC90B PE=1 SV=2	CCDC90B	1.478	5	1	1	1	254	29.5	7.55
#256	P22087	rRNA 2'-O-methyltransferase fibrillarin OS=Homo sapiens OX=9606 GN=FBL PE=1 SV=2	FBL	1.443	4	1	1	1	321	33.8	10.18
#257	P07477	Trypsin-1 OS=Homo sapiens OX=9606 GN=PRSS1 PE=1 SV=1	PRSS1	1.419	3	1	1	1	247	26.5	6.51
#258	O14744	Protein arginine N-methyltransferase 5 OS=Homo sapiens OX=9606 GN=PRMT5 PE=1 SV=4	PRMT5	1.339	2	1	1	1	637	72.6	6.29
#259	O00148	ATP-dependent RNA helicase DDX39A OS=Homo sapiens OX=9606 GN=DDX39A PE=1 SV=2	DDX39A	1.278	3	1	1	1	427	49.1	5.68
#260	Q9Y3U8	60S ribosomal protein L36 OS=Homo sapiens OX=9606 GN=RPL36 PE=1 SV=3	RPL36	1.259	10	1	1	1	105	12.2	11.59
#261	P04406	Glyceraldehyde-3-phosphate dehydrogenase OS=Homo sapiens OX=9606 GN=GAPDH PE=1 SV=3	GAPDH	1.244	3	1	1	1	335	36	8.46

#262	P05198	Eukaryotic translation initiation factor 2 subunit 1 OS=Homo sapiens OX=9606 GN=EIF2S1 PE=1 SV=3	EIF2S1	1.228	4	1	1	1	315	36.1	5.08
#263	Q15695	Putative U2 small nuclear ribonucleoprotein auxiliary factor 35 kDa subunit-related protein 1 OS=Homo sapiens OX=9606 GN=ZRSR2P1 PE=5 SV=2	ZRSR1	1.201	1	1	1	1	479	57.6	9.36
#264	P26599	Polypyrimidine tract-binding protein 1 OS=Homo sapiens OX=9606 GN=PTBP1 PE=1 SV=1	PTBP1	1.197	2	1	1	1	531	57.2	9.17
#265	Q5SY80	Cation channel sperm-associated protein subunit epsilon OS=Homo sapiens OX=9606 GN=CATSPERE PE=2 SV=1	C1orf101; CATSPERE	1.195	2	1	1	1	951	109.6	7.27
#266	P46087	Probable 28S rRNA (cytosine(4447)-C(5))-methyltransferase OS=Homo sapiens OX=9606 GN=NOP2 PE=1 SV=2	NOP2	1.168	1	1	1	1	812	89.2	9.23
#267	Q16629	Serine/arginine-rich splicing factor 7 OS=Homo sapiens OX=9606 GN=SRSF7 PE=1 SV=1	SRSF7	1.153	5	1	1	1	238	27.4	11.82
#268	P14314	Glucosidase 2 subunit beta OS=Homo sapiens OX=9606 GN=PRKCSH PE=1 SV=2	PRKCSH	1.119	5	1	1	1	528	59.4	4.41
#269	Q9NY12	H/ACA ribonucleoprotein complex subunit 1 OS=Homo sapiens OX=9606 GN=GAR1 PE=1 SV=1	GAR1	1.117	4	1	1	1	217	22.3	10.92

#270	Q5BKY9	Protein FAM133B OS=Homo sapiens OX=9606 GN=FAM133B PE=1 SV=1	FAM133B	1.116	4	1	1	1	247	28.4	10.02
#271	P35637	RNA-binding protein FUS OS=Homo sapiens OX=9606 GN=FUS PE=1 SV=1	FUS	1.115	3	1	1	1	526	53.4	9.36
#272	Q86VY4	Testis-specific Y-encoded-like protein 5 OS=Homo sapiens OX=9606 GN=TSPYL5 PE=1 SV=2	TSPYL5	1.115	2	1	1	1	417	45.1	9.54
#273	P24539	ATP synthase F(0) complex subunit B1, mitochondrial OS=Homo sapiens OX=9606 GN=ATP5PB PE=1 SV=2	ATP5F1	1.111	3	1	1	1	256	28.9	9.36
#274	O75494	Serine/arginine-rich splicing factor 10 OS=Homo sapiens OX=9606 GN=SRSF10 PE=1 SV=1	SRSF10; LOC100996657	1.104	4	1	1	1	262	31.3	11.27
#275	P19367	Hexokinase-1 OS=Homo sapiens OX=9606 GN=HK1 PE=1 SV=3	HK1	1.094	1	1	1	1	917	102.4	6.8
#276	Q9NQ90	Anoctamin-2 OS=Homo sapiens OX=9606 GN=ANO2 PE=1 SV=2	ANO2	1.091	1	1	1	1	1003	113.9	6.55
#277	P23083	Immunoglobulin heavy variable 1-2 OS=Homo sapiens OX=9606 GN=IGHV1-2 PE=1 SV=2	IGHV1OR15-1	1.081	9	1	1	1	117	13.1	9.13
#278	P35241	Radixin OS=Homo sapiens OX=9606 GN=RDX PE=1 SV=1	RDX	1.054	1	1	1	1	583	68.5	6.37

#279	Q16698	2,4-dienoyl-CoA reductase, mitochondrial OS=Homo sapiens OX=9606 GN=DECR1 PE=1 SV=1	DECR1	1.047	4	1	1	1	335	36	9.28
#280	Q12905	Interleukin enhancer-binding factor 2 OS=Homo sapiens OX=9606 GN=ILF2 PE=1 SV=2	ILF2	1.047	4	1	1	1	390	43	5.26
#281	P63208	S-phase kinase-associated protein 1 OS=Homo sapiens OX=9606 GN=SKP1 PE=1 SV=2	SKP1	1.044	10	1	1	1	163	18.6	4.54
#282	P82933	28S ribosomal protein S9, mitochondrial OS=Homo sapiens OX=9606 GN=MRPS9 PE=1 SV=2	MRPS9	1.037	3	1	1	1	396	45.8	9.51
#283	P23528	Cofilin-1 OS=Homo sapiens OX=9606 GN=CFL1 PE=1 SV=3	CFL1	1.035	8	1	1	1	166	18.5	8.09
#284	P12956	X-ray repair cross-complementing protein 6 OS=Homo sapiens OX=9606 GN=XRCC6 PE=1 SV=2	XRCC6	1.018	1	1	1	1	609	69.8	6.64
#285	Q86YZ3	Hornerin OS=Homo sapiens OX=9606 GN=HRNR PE=1 SV=2	HRNR	1.009	2	1	1	1	2850	282.2	10.04
#286	P84243	Histone H3.3 OS=Homo sapiens OX=9606 GN=H3-3A PE=1 SV=2	H3F3A; H3F3A P4; H3F3B	1.007	5	1	2	1	136	15.3	11.27
#287	P31040	Succinate dehydrogenase [ubiquinone] flavoprotein subunit, mitochondrial OS=Homo sapiens OX=9606 GN=SDHA PE=1 SV=2	SDHA	0.975	2	1	1	1	664	72.6	7.39

#288	Q9NYF8	Bcl-2-associated transcription factor 1 OS=Homo sapiens OX=9606 GN=BCLAF1 PE=1 SV=2	BCLAF 1	0.958	1	1	1	1	920	106.1	9.98
#289	Q13435	Splicing factor 3B subunit 2 OS=Homo sapiens OX=9606 GN=SF3B2 PE=1 SV=2	SF3B2	0.948	1	1	1	1	895	100.2	5.67
#290	Q9NWB6	Arginine and glutamate-rich protein 1 OS=Homo sapiens OX=9606 GN=ARGLU1 PE=1 SV=1	ARGLU 1	0.946	2	1	2	1	273	33.2	10.35
#291	Q02978	Mitochondrial 2-oxoglutarate/malate carrier protein OS=Homo sapiens OX=9606 GN=SLC25A11 PE=1 SV=3	SLC25 A11	0.945	5	1	1	1	314	34	9.91
#292	Q9BZE4	Nucleolar GTP-binding protein 1 OS=Homo sapiens OX=9606 GN=GTPBP4 PE=1 SV=3	GTPBP 4	0.939	1	1	1	1	634	73.9	9.5
#293	Q9BQA5	Histone H4 transcription factor OS=Homo sapiens OX=9606 GN=HINFP PE=1 SV=2	HINFP	0.926	1	1	1	1	517	59.6	6.19
#294	P48739	Phosphatidylinositol transfer protein beta isoform OS=Homo sapiens OX=9606 GN=PITPNB PE=1 SV=2	PITPNB	0.909	4	1	1	1	271	31.5	6.87

Protein list for gemcitabine

No.	Accession	Description	Gene Symbol	Sum PEP Score	Coverage [%]	# Peptides	# PSMs	# Unique Peptides	# AAs	MW [kDa]	calc. pI
#1	P04264	Keratin, type II cytoskeletal 1 OS=Homo sapiens OX=9606 GN=KRT1 PE=1 SV=6	KRT1	38.21	26	17	33	14	644	66	8.12

#2	P06576	ATP synthase subunit beta, mitochondrial OS=Homo sapiens OX=9606 GN=ATP5F1B PE=1 SV=3	ATP5B	32.248	25	7	15	7	529	56.5	5.4
#3	P35908	Keratin, type II cytoskeletal 2 epidermal OS=Homo sapiens OX=9606 GN=KRT2 PE=1 SV=2	KRT2	31.829	24	14	20	10	639	65.4	8
#4	P19338	Nucleolin OS=Homo sapiens OX=9606 GN=NCL PE=1 SV=3	NCL	30.736	21	16	24	16	710	76.6	4.7
#5	P35527	Keratin, type I cytoskeletal 9 OS=Homo sapiens OX=9606 GN=KRT9 PE=1 SV=3	KRT9	29.45	23	12	24	11	623	62	5.24
#6	P13645	Keratin, type I cytoskeletal 10 OS=Homo sapiens OX=9606 GN=KRT10 PE=1 SV=6	KRT10	28.357	24	14	24	12	584	58.8	5.21
#7	P25705	ATP synthase subunit alpha, mitochondrial OS=Homo sapiens OX=9606 GN=ATP5F1A PE=1 SV=1	ATP5A 1	26.282	20	9	26	9	553	59.7	9.13
#8	P05387	60S acidic ribosomal protein P2 OS=Homo sapiens OX=9606 GN=RPLP2 PE=1 SV=1	RPLP2	21.962	70	6	13	6	115	11.7	4.54
#9	P67809	Y-box-binding protein 1 OS=Homo sapiens OX=9606 GN=YBX1 PE=1 SV=3	YBX1	21.414	32	6	10	6	324	35.9	9.88
#10	Q9NRW3	DNA dC->dU-editing enzyme APOBEC-3C OS=Homo sapiens OX=9606 GN=APOBEC3C PE=1 SV=2	APOBE C3C	20.68	36	5	11	5	190	22.8	7.59
#11	P62241	40S ribosomal protein S8 OS=Homo sapiens OX=9606 GN=RPS8 PE=1 SV=2	RPS8	19.162	39	7	13	7	208	24.2	10.32

#12	P36578	60S ribosomal protein L4 OS=Homo sapiens OX=9606 GN=RPL4 PE=1 SV=5	RPL4	18.12	19	7	15	7	427	47.7	11.06
#13	Q00839	Heterogeneous nuclear ribonucleoprotein U OS=Homo sapiens OX=9606 GN=HNRNPU PE=1 SV=6	HNRNP U	16.417	13	11	18	11	825	90.5	6
#14	P05023	Sodium/potassium-transporting ATPase subunit alpha-1 OS=Homo sapiens OX=9606 GN=ATP1A1 PE=1 SV=1	ATP1A 1	14.578	5	3	4	3	1023	112.8	5.49
#15	P16403	Histone H1.2 OS=Homo sapiens OX=9606 GN=H1-2 PE=1 SV=2	HIST1H 1C	14.502	31	7	10	5	213	21.4	10.93
#16	P05388	60S acidic ribosomal protein P0 OS=Homo sapiens OX=9606 GN=RPLP0 PE=1 SV=1	RPLP0	14.303	20	5	6	5	317	34.3	5.97
#17	Q07020	60S ribosomal protein L18 OS=Homo sapiens OX=9606 GN=RPL18 PE=1 SV=2	RPL18	13.286	26	4	11	4	188	21.6	11.72
#18	P21796	Voltage-dependent anion-selective channel protein 1 OS=Homo sapiens OX=9606 GN=VDAC1 PE=1 SV=2	VDAC1	12.48	22	3	4	3	283	30.8	8.54
#19	P16401	Histone H1.5 OS=Homo sapiens OX=9606 GN=H1-5 PE=1 SV=3	HIST1H 1B	11.126	22	6	6	4	226	22.6	10.92
#20	P09651	Heterogeneous nuclear ribonucleoprotein A1 OS=Homo sapiens OX=9606 GN=HNRNPA1 PE=1 SV=5	HNRNP A1	10.73	17	6	7	5	372	38.7	9.13
#21	P13647	Keratin, type II cytoskeletal 5 OS=Homo sapiens OX=9606 GN=KRT5 PE=1 SV=3	KRT5	10.602	10	6	11	3	590	62.3	7.74

#22	P62701	40S ribosomal protein S4, X isoform OS=Homo sapiens OX=9606 GN=RPS4X PE=1 SV=2	RPS4X	10.377	28	8	10	8	263	29.6	10.15
#23	O95831	Apoptosis-inducing factor 1, mitochondrial OS=Homo sapiens OX=9606 GN=AIFM1 PE=1 SV=1	AIFM1	10.217	8	3	3	3	613	66.9	8.95
#24	P62424	60S ribosomal protein L7a OS=Homo sapiens OX=9606 GN=RPL7A PE=1 SV=2	RPL7A	10.112	24	5	7	5	266	30	10.61
#25	A2NJV5	Immunoglobulin kappa variable 2-29 OS=Homo sapiens OX=9606 GN=IGKV2-29 PE=3 SV=2	IGKV2-29	9.857	19	3	17	2	120	13.1	7.28
#26	P18621	60S ribosomal protein L17 OS=Homo sapiens OX=9606 GN=RPL17 PE=1 SV=3	RPL17	9.433	21	4	7	4	184	21.4	10.17
#27	Q02878	60S ribosomal protein L6 OS=Homo sapiens OX=9606 GN=RPL6 PE=1 SV=3	RPL6	9.34	24	7	11	7	288	32.7	10.58
#28	P05141	ADP/ATP translocase 2 OS=Homo sapiens OX=9606 GN=SLC25A5 PE=1 SV=7	SLC25A5	9.328	11	3	9	3	298	32.8	9.69
#29	P11940	Polyadenylate-binding protein 1 OS=Homo sapiens OX=9606 GN=PABPC1 PE=1 SV=2	PABPC1	9.269	12	6	8	6	636	70.6	9.5
#30	Q05639	Elongation factor 1-alpha 2 OS=Homo sapiens OX=9606 GN=EEF1A2 PE=1 SV=1	EEF1A2	9.131	8	3	4	3	463	50.4	9.03
#31	P62851	40S ribosomal protein S25 OS=Homo sapiens OX=9606 GN=RPS25 PE=1 SV=1	RPS25	8.69	24	4	7	4	125	13.7	10.11
#32	P30050	60S ribosomal protein L12 OS=Homo sapiens OX=9606 GN=RPL12 PE=1 SV=1	RPL12	8.681	39	5	6	5	165	17.8	9.42

#33	P26373	60S ribosomal protein L13 OS=Homo sapiens OX=9606 GN=RPL13 PE=1 SV=4	RPL13	8.348	22	5	6	5	211	24.2	11.65
#34	P62888	60S ribosomal protein L30 OS=Homo sapiens OX=9606 GN=RPL30 PE=1 SV=2	RPL30	7.926	24	2	4	2	115	12.8	9.63
#35	P18124	60S ribosomal protein L7 OS=Homo sapiens OX=9606 GN=RPL7 PE=1 SV=1	RPL7	7.925	20	5	6	5	248	29.2	10.65
#36	P06748	Nucleophosmin OS=Homo sapiens OX=9606 GN=NPM1 PE=1 SV=2	NPM1	7.727	15	5	9	5	294	32.6	4.78
#37	P83731	60S ribosomal protein L24 OS=Homo sapiens OX=9606 GN=RPL24 PE=1 SV=1	RPL24	7.533	27	6	7	6	157	17.8	11.25
#38	Q02543	60S ribosomal protein L18a OS=Homo sapiens OX=9606 GN=RPL18A PE=1 SV=2	RPL18 A	7.417	17	3	6	3	176	20.7	10.71
#39	P62269	40S ribosomal protein S18 OS=Homo sapiens OX=9606 GN=RPS18 PE=1 SV=3	RPS18	7.325	28	5	6	5	152	17.7	10.99
#40	P40429	60S ribosomal protein L13a OS=Homo sapiens OX=9606 GN=RPL13A PE=1 SV=2	RPL13 A	7.318	17	3	3	3	203	23.6	10.93
#41	P46779	60S ribosomal protein L28 OS=Homo sapiens OX=9606 GN=RPL28 PE=1 SV=3	RPL28	7.242	15	3	3	3	137	15.7	12.02
#42	P50914	60S ribosomal protein L14 OS=Homo sapiens OX=9606 GN=RPL14 PE=1 SV=4	RPL14	7.048	13	3	8	3	215	23.4	10.93
#43	P62829	60S ribosomal protein L23 OS=Homo sapiens OX=9606 GN=RPL23 PE=1 SV=1	RPL23	7.038	33	4	5	4	140	14.9	10.51

#44	O60506	Heterogeneous nuclear ribonucleoprotein Q OS=Homo sapiens OX=9606 GN=SYNCRIP PE=1 SV=2	SYNCRIP	6.832	6	3	5	3	623	69.6	8.59
#45	P62249	40S ribosomal protein S16 OS=Homo sapiens OX=9606 GN=RPS16 PE=1 SV=2	RPS16	6.8	24	5	6	5	146	16.4	10.21
#46	P46776	60S ribosomal protein L27a OS=Homo sapiens OX=9606 GN=RPL27A PE=1 SV=2	RPL27A	6.658	22	3	4	3	148	16.6	11
#47	P31943	Heterogeneous nuclear ribonucleoprotein H OS=Homo sapiens OX=9606 GN=HNRNPH1 PE=1 SV=4	HNRNPH1	6.57	9	3	3	3	449	49.2	6.3
#48	P46781	40S ribosomal protein S9 OS=Homo sapiens OX=9606 GN=RPS9 PE=1 SV=3	RPS9	6.548	22	5	8	5	194	22.6	10.65
#49	P61313	60S ribosomal protein L15 OS=Homo sapiens OX=9606 GN=RPL15 PE=1 SV=2	RPL15	6.482	16	3	4	3	204	24.1	11.62
#50	P10809	60 kDa heat shock protein, mitochondrial OS=Homo sapiens OX=9606 GN=HSPD1 PE=1 SV=2	HSPD1	6.447	5	2	4	2	573	61	5.87
#51	P62899	60S ribosomal protein L31 OS=Homo sapiens OX=9606 GN=RPL31 PE=1 SV=1	RPL31	6.31	30	4	5	4	125	14.5	10.54
#52	P62280	40S ribosomal protein S11 OS=Homo sapiens OX=9606 GN=RPS11 PE=1 SV=3	RPS11	6.206	23	3	7	3	158	18.4	10.3
#53	P15880	40S ribosomal protein S2 OS=Homo sapiens OX=9606 GN=RPS2 PE=1 SV=2	RPS2	6.173	12	3	6	3	293	31.3	10.24

#54	P62906	60S ribosomal protein L10a OS=Homo sapiens OX=9606 GN=RPL10A PE=1 SV=2	RPL10 A	6.126	17	3	4	3	217	24.8	9.94
#55	P46778	60S ribosomal protein L21 OS=Homo sapiens OX=9606 GN=RPL21 PE=1 SV=2	RPL21	5.58	9	1	2	1	160	18.6	10.49
#56	P62750	60S ribosomal protein L23a OS=Homo sapiens OX=9606 GN=RPL23A PE=1 SV=1	RPL23 A	5.543	21	3	3	3	156	17.7	10.45
#57	Q86V81	THO complex subunit 4 OS=Homo sapiens OX=9606 GN=ALYREF PE=1 SV=3	ALYRE F	5.089	14	3	3	3	257	26.9	11.15
#58	Q12906	Interleukin enhancer-binding factor 3 OS=Homo sapiens OX=9606 GN=ILF3 PE=1 SV=3	ILF3	5.084	4	3	3	3	894	95.3	8.76
#59	P22626	Heterogeneous nuclear ribonucleoproteins A2/B1 OS=Homo sapiens OX=9606 GN=HNRNPA2B1 PE=1 SV=2	HNRNP A2B1	4.994	10	3	4	2	353	37.4	8.95
#60	Q6PKH6	Dehydrogenase/reductase SDR family member 4-like 2 OS=Homo sapiens OX=9606 GN=DHRS4L2 PE=2 SV=1	DHRS4 L2	4.892	7	1	1	1	230	24.6	10.11
#61	P61247	40S ribosomal protein S3a OS=Homo sapiens OX=9606 GN=RPS3A PE=1 SV=2	RPS3A	4.818	15	4	6	4	264	29.9	9.73
#62	P62913	60S ribosomal protein L11 OS=Homo sapiens OX=9606 GN=RPL11 PE=1 SV=2	RPL11	4.64	17	3	7	3	178	20.2	9.6
#63	P62266	40S ribosomal protein S23 OS=Homo sapiens OX=9606 GN=RPS23 PE=1 SV=3	RPS23	4.602	16	2	4	2	143	15.8	10.49

#64	P52732	Kinesin-like protein KIF11 OS=Homo sapiens OX=9606 GN=KIF11 PE=1 SV=2	KIF11	4.596	4	4	7	4	1056	119.1	5.64
#65	P07237	Protein disulfide-isomerase OS=Homo sapiens OX=9606 GN=P4HB PE=1 SV=3	P4HB	4.513	3	1	2	1	508	57.1	4.87
#66	Q8N8Y2	V-type proton ATPase subunit d 2 OS=Homo sapiens OX=9606 GN=ATP6V0D2 PE=1 SV=1	ATP6V 0D2	4.505	5	1	1	1	350	40.4	5.3
#67	P38159	RNA-binding motif protein, X chromosome OS=Homo sapiens OX=9606 GN=RBMX PE=1 SV=3	RBMX	4.417	8	3	3	3	391	42.3	10.05
#68	P62244	40S ribosomal protein S15a OS=Homo sapiens OX=9606 GN=RPS15A PE=1 SV=2	RPS15 A	4.406	22	3	9	3	130	14.8	10.13
#69	P35268	60S ribosomal protein L22 OS=Homo sapiens OX=9606 GN=RPL22 PE=1 SV=2	RPL22	4.386	19	2	3	2	128	14.8	9.19
#70	P62753	40S ribosomal protein S6 OS=Homo sapiens OX=9606 GN=RPS6 PE=1 SV=1	RPS6	4.379	11	3	7	3	249	28.7	10.84
#71	P17844	Probable ATP-dependent RNA helicase DDX5 OS=Homo sapiens OX=9606 GN=DDX5 PE=1 SV=1	DDX5	4.376	6	3	3	3	614	69.1	8.92
#72	P62917	60S ribosomal protein L8 OS=Homo sapiens OX=9606 GN=RPL8 PE=1 SV=2	RPL8	4.329	15	3	6	3	257	28	11.03
#73	P28331	NADH-ubiquinone oxidoreductase 75 kDa subunit, mitochondrial OS=Homo sapiens OX=9606 GN=NDUFS1 PE=1 SV=3	NDUFS 1	4.323	5	2	3	2	727	79.4	6.23

#74	P84090	Enhancer of rudimentary homolog OS=Homo sapiens OX=9606 GN=ERH PE=1 SV=1	ERH	4.245	12	1	1	1	104	12.3	5.92
#75	Q9UJS0	Calcium-binding mitochondrial carrier protein Aralar2 OS=Homo sapiens OX=9606 GN=SLC25A13 PE=1 SV=2	SLC25A13	4.188	4	2	2	2	675	74.1	8.62
#76	P62277	40S ribosomal protein S13 OS=Homo sapiens OX=9606 GN=RPS13 PE=1 SV=2	RPS13	4.123	21	3	5	3	151	17.2	10.54
#77	Q9NX63	MICOS complex subunit MIC19 OS=Homo sapiens OX=9606 GN=CHCHD3 PE=1 SV=1	CHCHD3	4.084	7	1	5	1	227	26.1	8.28
#78	P25398	40S ribosomal protein S12 OS=Homo sapiens OX=9606 GN=RPS12 PE=1 SV=3	RPS12	4.065	26	3	4	3	132	14.5	7.21
#79	P31930	Cytochrome b-c1 complex subunit 1, mitochondrial OS=Homo sapiens OX=9606 GN=UQCRC1 PE=1 SV=3	UQCRC1	4.048	4	1	2	1	480	52.6	6.37
#80	P39023	60S ribosomal protein L3 OS=Homo sapiens OX=9606 GN=RPL3 PE=1 SV=2	RPL3	3.915	6	2	3	2	403	46.1	10.18
#81	P02533	Keratin, type I cytoskeletal 14 OS=Homo sapiens OX=9606 GN=KRT14 PE=1 SV=4	KRT14	3.907	8	4	6	2	472	51.5	5.16
#82	P84098	60S ribosomal protein L19 OS=Homo sapiens OX=9606 GN=RPL19 PE=1 SV=1	RPL19	3.89	8	2	6	2	196	23.5	11.47
#83	P61254	60S ribosomal protein L26 OS=Homo sapiens OX=9606 GN=RPL26 PE=1 SV=1	RPL26	3.768	12	2	5	2	145	17.2	10.55

#84	Q14103	Heterogeneous nuclear ribonucleoprotein D0 OS=Homo sapiens OX=9606 GN=HNRNPD PE=1 SV=1	HNRNPD	3.666	7	2	2	1	355	38.4	7.81
#85	Q9BUJ2	Heterogeneous nuclear ribonucleoprotein U-like protein 1 OS=Homo sapiens OX=9606 GN=HNRNPUL1 PE=1 SV=2	HNRNPUL1	3.595	3	3	3	3	856	95.7	6.92
#86	P60709	Actin, cytoplasmic 1 OS=Homo sapiens OX=9606 GN=ACTB PE=1 SV=1	ACTB	3.544	7	2	2	2	375	41.7	5.48
#87	P02768	Albumin OS=Homo sapiens OX=9606 GN=ALB PE=1 SV=2	ALB	3.41	3	2	2	2	609	69.3	6.28
#88	P21281	V-type proton ATPase subunit B, brain isoform OS=Homo sapiens OX=9606 GN=ATP6V1B2 PE=1 SV=3	ATP6V1B2	3.312	6	2	2	2	511	56.5	5.81
#89	P47914	60S ribosomal protein L29 OS=Homo sapiens OX=9606 GN=RPL29 PE=1 SV=2	RPL29	3.192	14	2	2	2	159	17.7	11.66
#90	P08865	40S ribosomal protein SA OS=Homo sapiens OX=9606 GN=RPSA PE=1 SV=4	RPSA	3.001	10	2	3	2	295	32.8	4.87
#91	P35241	Radixin OS=Homo sapiens OX=9606 GN=RDX PE=1 SV=1	RDX	2.967	3	1	1	1	583	68.5	6.37
#92	P62841	40S ribosomal protein S15 OS=Homo sapiens OX=9606 GN=RPS15 PE=1 SV=2	RPS15	2.8	9	1	1	1	145	17	10.39
#93	O00622	CCN family member 1 OS=Homo sapiens OX=9606 GN=CCN1 PE=1 SV=1	CYR61	2.763	5	2	2	2	381	42	8.21

#94	P49207	60S ribosomal protein L34 OS=Homo sapiens OX=9606 GN=RPL34 PE=1 SV=3	RPL34	2.749	21	3	6	3	117	13.3	11.47
#95	Q13151	Heterogeneous nuclear ribonucleoprotein A0 OS=Homo sapiens OX=9606 GN=HNRNPA0 PE=1 SV=1	HNRNP A0	2.678	5	1	2	1	305	30.8	9.29
#96	P62910	60S ribosomal protein L32 OS=Homo sapiens OX=9606 GN=RPL32 PE=1 SV=2	RPL32	2.674	13	2	2	2	135	15.9	11.33
#97	P61353	60S ribosomal protein L27 OS=Homo sapiens OX=9606 GN=RPL27 PE=1 SV=2	RPL27	2.623	11	2	2	2	136	15.8	10.56
#98	A0A075B 6R9	Probable non-functional immunoglobulin kappa variable 2D-24 OS=Homo sapiens OX=9606 GN=IGKV2D-24 PE=1 SV=1		2.603	13	2	7	1	120	13.1	8.87
#99	Q9BRJ6	Uncharacterized protein C7orf50 OS=Homo sapiens OX=9606 GN=C7orf50 PE=1 SV=1	C7orf50	2.559	11	1	1	1	194	22.1	9.64
#100	P23396	40S ribosomal protein S3 OS=Homo sapiens OX=9606 GN=RPS3 PE=1 SV=2	RPS3	2.532	9	2	2	2	243	26.7	9.66
#101	Q9NZI8	Insulin-like growth factor 2 mRNA-binding protein 1 OS=Homo sapiens OX=9606 GN=IGF2BP1 PE=1 SV=2	IGF2BP 1	2.514	4	2	4	2	577	63.4	9.2
#102	P62263	40S ribosomal protein S14 OS=Homo sapiens OX=9606 GN=RPS14 PE=1 SV=3	RPS14	2.472	15	2	4	2	151	16.3	10.05
#103	Q99729	Heterogeneous nuclear ribonucleoprotein A/B OS=Homo sapiens OX=9606 GN=HNRNPAB PE=1 SV=2	HNRNP AB	2.461	5	2	2	1	332	36.2	8.21

#104	Q99623	Prohibitin-2 OS=Homo sapiens OX=9606 GN=PHB2 PE=1 SV=2	PHB2	2.429	4	1	1	1	299	33.3	9.83
#105	E9PRG8	Uncharacterized protein C11orf98 OS=Homo sapiens OX=9606 GN=C11orf98 PE=4 SV=2		2.426	11	1	2	1	123	14.2	11.53
#106	P31040	Succinate dehydrogenase [ubiquinone] flavoprotein subunit, mitochondrial OS=Homo sapiens OX=9606 GN=SDHA PE=1 SV=2	SDHA	2.426	2	1	1	1	664	72.6	7.39
#107	Q92522	Histone H1.10 OS=Homo sapiens OX=9606 GN=H1-10 PE=1 SV=1	H1FX	2.363	12	2	2	2	213	22.5	10.76
#108	P61978	Heterogeneous nuclear ribonucleoprotein K OS=Homo sapiens OX=9606 GN=HNRNPK PE=1 SV=1	HNRNPK	2.22	3	1	1	1	463	50.9	5.54
#109	O95881	Thioredoxin domain-containing protein 12 OS=Homo sapiens OX=9606 GN=TXNDC12 PE=1 SV=1	TXNDC12	2.05	9	2	3	2	172	19.2	5.4
#110	P27797	Calreticulin OS=Homo sapiens OX=9606 GN=CALR PE=1 SV=1	CALR	1.995	3	1	1	1	417	48.1	4.44
#111	O00571	ATP-dependent RNA helicase DDX3X OS=Homo sapiens OX=9606 GN=DDX3X PE=1 SV=3	DDX3X	1.964	3	2	2	2	662	73.2	7.18
#112	P10599	Thioredoxin OS=Homo sapiens OX=9606 GN=TXN PE=1 SV=3	TXN	1.943	12	1	2	1	105	11.7	4.92
#113	P42766	60S ribosomal protein L35 OS=Homo sapiens OX=9606 GN=RPL35 PE=1 SV=2	RPL35	1.924	7	1	1	1	123	14.5	11.05

#114	P01859	Immunoglobulin heavy constant gamma 2 OS=Homo sapiens OX=9606 GN=IGHG2 PE=1 SV=2	IGHG2	1.884	3	1	4	1	326	35.9	7.59
#115	O00483	Cytochrome c oxidase subunit NDUFA4 OS=Homo sapiens OX=9606 GN=NDUFA4 PE=1 SV=1	NDUFA 4	1.816	12	1	2	1	81	9.4	9.38
#116	P61513	60S ribosomal protein L37a OS=Homo sapiens OX=9606 GN=RPL37A PE=1 SV=2	RPL37 A	1.743	10	1	3	1	92	10.3	10.43
#117	P48741	Putative heat shock 70 kDa protein 7 OS=Homo sapiens OX=9606 GN=HSPA7 PE=5 SV=2	HSPA7	1.714	4	1	1	1	367	40.2	7.87
#118	P38606	V-type proton ATPase catalytic subunit A OS=Homo sapiens OX=9606 GN=ATP6V1A PE=1 SV=2	ATP6V 1A	1.713	2	1	1	1	617	68.3	5.52
#119	P08708	40S ribosomal protein S17 OS=Homo sapiens OX=9606 GN=RPS17 PE=1 SV=2	RPS17; RPS17L	1.694	8	1	1	1	135	15.5	9.85
#120	P81605	Dermcidin OS=Homo sapiens OX=9606 GN=DCD PE=1 SV=2	DCD	1.674	10	1	1	1	110	11.3	6.54
#121	P62987	Ubiquitin-60S ribosomal protein L40 OS=Homo sapiens OX=9606 GN=UBA52 PE=1 SV=2	UBA52	1.662	7	1	1	1	128	14.7	9.83
#122	P18077	60S ribosomal protein L35a OS=Homo sapiens OX=9606 GN=RPL35A PE=1 SV=2	RPL35 A	1.636	11	1	2	1	110	12.5	11.06
#123	P02100	Hemoglobin subunit epsilon OS=Homo sapiens OX=9606 GN=HBE1 PE=1 SV=2	HBE1	1.59	7	1	2	1	147	16.2	8.63

#124	O14975	Very long-chain acyl-CoA synthetase OS=Homo sapiens OX=9606 GN=SLC27A2 PE=1 SV=2	SLC27 A2	1.441	2	1	1	1	620	70.3	8.51
#125	P32969	60S ribosomal protein L9 OS=Homo sapiens OX=9606 GN=RPL9 PE=1 SV=1	RPL9	1.441	5	1	1	1	192	21.9	9.95
#126	P05062	Fructose-bisphosphate aldolase B OS=Homo sapiens OX=9606 GN=ALDOB PE=1 SV=2	ALDOB	1.379	4	1	1	1	364	39.4	7.87
#127	Q9Y277	Voltage-dependent anion-selective channel protein 3 OS=Homo sapiens OX=9606 GN=VDAC3 PE=1 SV=1	VDAC3	1.347	4	1	1	1	283	30.6	8.66
#128	P10636	Microtubule-associated protein tau OS=Homo sapiens OX=9606 GN=MAPT PE=1 SV=5	MAPT	1.344	1	1	2	1	758	78.9	6.71
#129	Q9UM54	Unconventional myosin-VI OS=Homo sapiens OX=9606 GN=MYO6 PE=1 SV=4	MYO6	1.323	1	1	1	1	1294	149.6	8.53
#130	Q08211	ATP-dependent RNA helicase A OS=Homo sapiens OX=9606 GN=DHX9 PE=1 SV=4	DHX9	1.282	1	1	1	1	1270	140.9	6.84
#131	P50213	Isocitrate dehydrogenase [NAD] subunit alpha, mitochondrial OS=Homo sapiens OX=9606 GN=IDH3A PE=1 SV=1	IDH3A	1.263	3	1	1	1	366	39.6	6.92
#132	O75306	NADH dehydrogenase [ubiquinone] iron-sulfur protein 2, mitochondrial OS=Homo sapiens OX=9606 GN=NDUFS2 PE=1 SV=2	NDUFS 2	1.242	4	1	1	1	463	52.5	7.55
#133	P62854	40S ribosomal protein S26 OS=Homo sapiens OX=9606 GN=RPS26 PE=1 SV=3	RPS26; LOC10 192987	1.226	8	1	1	1	115	13	11

			6; RPS26P 25								
#134	P36957	Dihydrolipoyllysine-residue succinyltransferase component of 2-oxoglutarate dehydrogenase complex, mitochondrial OS=Homo sapiens OX=9606 GN=DLST PE=1 SV=4	DLST	1.214	3	1	1	1	453	48.7	8.95
#135	P07477	Trypsin-1 OS=Homo sapiens OX=9606 GN=PRSS1 PE=1 SV=1	PRSS1	1.174	3	1	3	1	247	26.5	6.51
#136	O14957	Cytochrome b-c1 complex subunit 10 OS=Homo sapiens OX=9606 GN=UQCR11 PE=1 SV=1	UQCR1 1	1.145	16	1	1	1	56	6.6	9.88
#137	P21741	Midkine OS=Homo sapiens OX=9606 GN=MDK PE=1 SV=1	MDK	1.11	7	1	1	1	143	15.6	9.79
#138	P60866	40S ribosomal protein S20 OS=Homo sapiens OX=9606 GN=RPS20 PE=1 SV=1	RPS20	1.108	6	1	1	1	119	13.4	9.94
#139	Q13427	Peptidyl-prolyl cis-trans isomerase G OS=Homo sapiens OX=9606 GN=PPIG PE=1 SV=2	PPIG	1.044	2	1	1	1	754	88.6	10.29
#140	P08574	Cytochrome c1, heme protein, mitochondrial OS=Homo sapiens OX=9606 GN=CYC1 PE=1 SV=3	CYC1	1.038	4	1	1	1	325	35.4	9
#141	Q15717	ELAV-like protein 1 OS=Homo sapiens OX=9606 GN=ELAVL1 PE=1 SV=2	ELAVL 1	1.037	4	1	1	1	326	36.1	9.17

#142	P49821	NADH dehydrogenase [ubiquinone] flavoprotein 1, mitochondrial OS=Homo sapiens OX=9606 GN=NDUFV1 PE=1 SV=4	NDUFV 1	0.988	3	1	1	1	464	50.8	8.21
#143	P27635	60S ribosomal protein L10 OS=Homo sapiens OX=9606 GN=RPL10 PE=1 SV=4	RPL10	0.988	4	1	2	1	214	24.6	10.08
#144	P52272	Heterogeneous nuclear ribonucleoprotein M OS=Homo sapiens OX=9606 GN=HNRNPM PE=1 SV=3	HNRNP M	0.973	3	1	1	1	730	77.5	8.7
#145	P40227	T-complex protein 1 subunit zeta OS=Homo sapiens OX=9606 GN=CCT6A PE=1 SV=3	CCT6A	0.968	2	1	1	1	531	58	6.68
#146	P40926	Malate dehydrogenase, mitochondrial OS=Homo sapiens OX=9606 GN=MDH2 PE=1 SV=3	MDH2	0.957	3	1	1	1	338	35.5	8.68
#147	P35637	RNA-binding protein FUS OS=Homo sapiens OX=9606 GN=FUS PE=1 SV=1	FUS	0.923	2	1	1	1	526	53.4	9.36
#148	P62805	Histone H4 OS=Homo sapiens OX=9606 GN=H4C1 PE=1 SV=2	HIST1H 4A; HIST1H 4F; HIST1H 4D; HIST1H 4J; HIST2H 4A;	0.905	10	1	1	1	103	11.4	11.36

			HIST2H4B; HIST1H4H; HIST1H4C; HIST4H4; HIST1H4E; HIST1H4I; HIST1H4B; HIST1H4K; HIST1H4L									
#149	Q9Y3U8	60S ribosomal protein L36 OS=Homo sapiens OX=9606 GN=RPL36 PE=1 SV=3	RPL36	0.897	10	1	1	1	105	12.2	11.59
#150	Q9C0A0	Contactin-associated protein-like 4 OS=Homo sapiens OX=9606 GN=CNTNAP4 PE=1 SV=3	CNTNAP4	0.887	1	1	1	1	1308	145.2	6.68
#151	P46087	Probable 28S rRNA (cytosine(4447)-C(5))-methyltransferase OS=Homo sapiens OX=9606 GN=NOP2 PE=1 SV=2	NOP2	0.85	1	1	1	1	812	89.2	9.23

#152	P62081	40S ribosomal protein S7 OS=Homo sapiens OX=9606 GN=RPS7 PE=1 SV=1	RPS7	0.807	5	1	1	1	194	22.1	10.1
#153	P35579	Myosin-9 OS=Homo sapiens OX=9606 GN=MYH9 PE=1 SV=4	MYH9	0.801	1	1	1	1	1960	226.4	5.6
#154	P45880	Voltage-dependent anion-selective channel protein 2 OS=Homo sapiens OX=9606 GN=VDAC2 PE=1 SV=2	VDAC2	0.752	3	1	1	1	294	31.5	7.56
#155	P39019	40S ribosomal protein S19 OS=Homo sapiens OX=9606 GN=RPS19 PE=1 SV=2	RPS19	0.749	6	1	1	1	145	16.1	10.32
#156	Q9BWX5	Transcription factor GATA-5 OS=Homo sapiens OX=9606 GN=GATA5 PE=1 SV=1	GATA5	0.747	2	1	1	1	397	41.3	8.9
#157	P38646	Stress-70 protein, mitochondrial OS=Homo sapiens OX=9606 GN=HSPA9 PE=1 SV=2	HSPA9	0.745	3	1	1	1	679	73.6	6.16
#158	A6NNA2	Serine/arginine repetitive matrix protein 3 OS=Homo sapiens OX=9606 GN=SRRM3 PE=2 SV=4	SRRM3	0.734	2	1	1	1	597	65.2	11.68
#159	P46777	60S ribosomal protein L5 OS=Homo sapiens OX=9606 GN=RPL5 PE=1 SV=3	RPL5	0.72	2	1	1	1	297	34.3	9.72
#160	P38935	DNA-binding protein SMUBP-2 OS=Homo sapiens OX=9606 GN=IGHMBP2 PE=1 SV=3	IGHMBP2	0.712	1	1	1	1	993	109.1	8.97
