# Supplementary material for: APOBEC3C coordinates DDX5 in R-loop resolution and dynamic control of Chk1-mediated stress-responsive circuitry as a prerequisite for gemcitabine resistance in p53-deficient cells
Source: Cell Death Dis. 2026 Jan 7;17(1):6. doi: 10.1038/s41419-025-08215-6 (PMC12780011; doi:10.1038/s41419-025-08215-6)

Fig. 2F

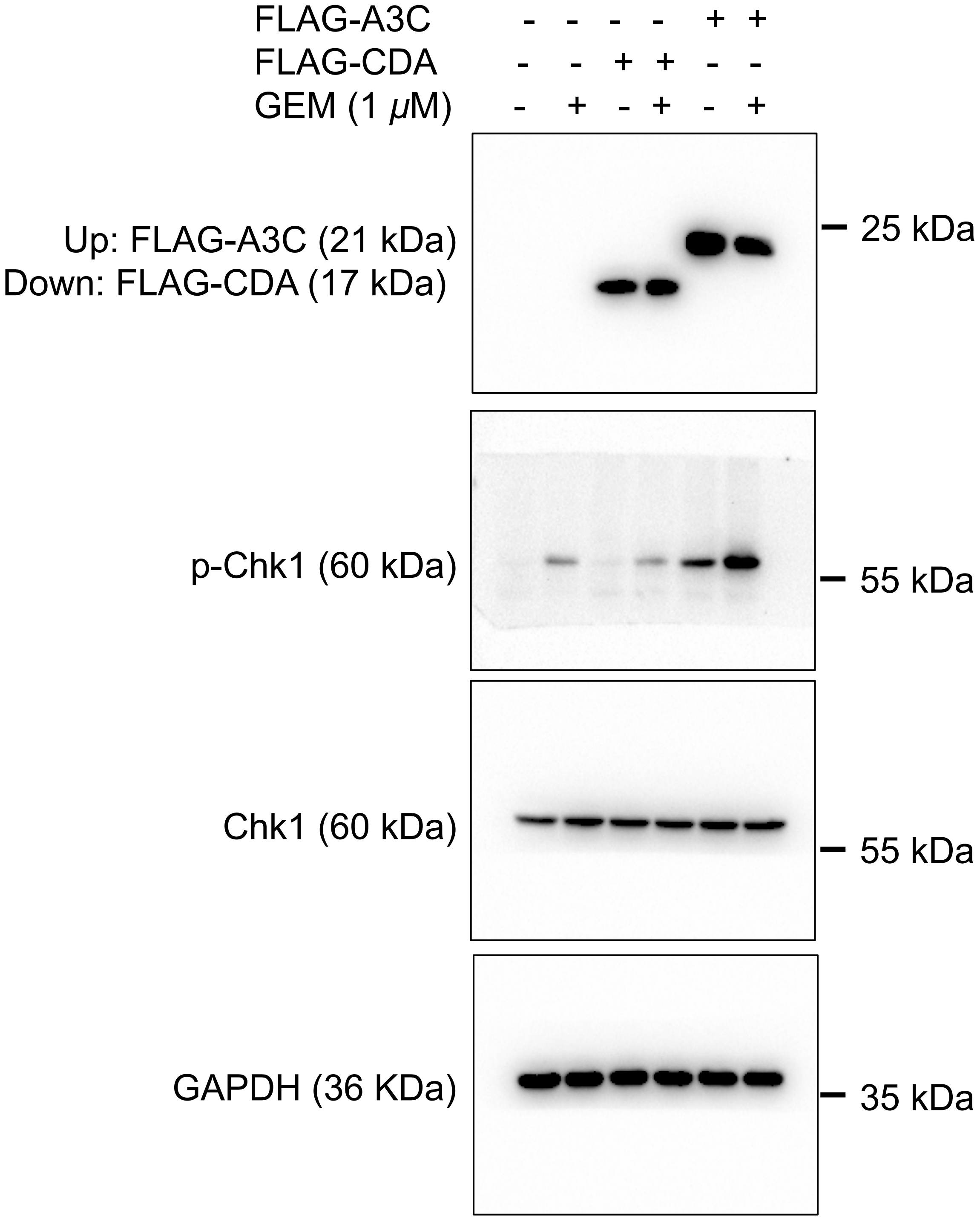
FLAG-A3C - - - - + +
FLAG-CDA - - + + - -
GEM (1 μM) - + - + - +
Up: FLAG-A3C (21 kDa)
Down: FLAG-CDA (17 kDa)
25 kDa
p-Chk1 (60 kDa)
55 kDa
Chk1 (60 kDa)
55 kDa
GAPDH (36 KDa)
35 kDa

Fig. 2G

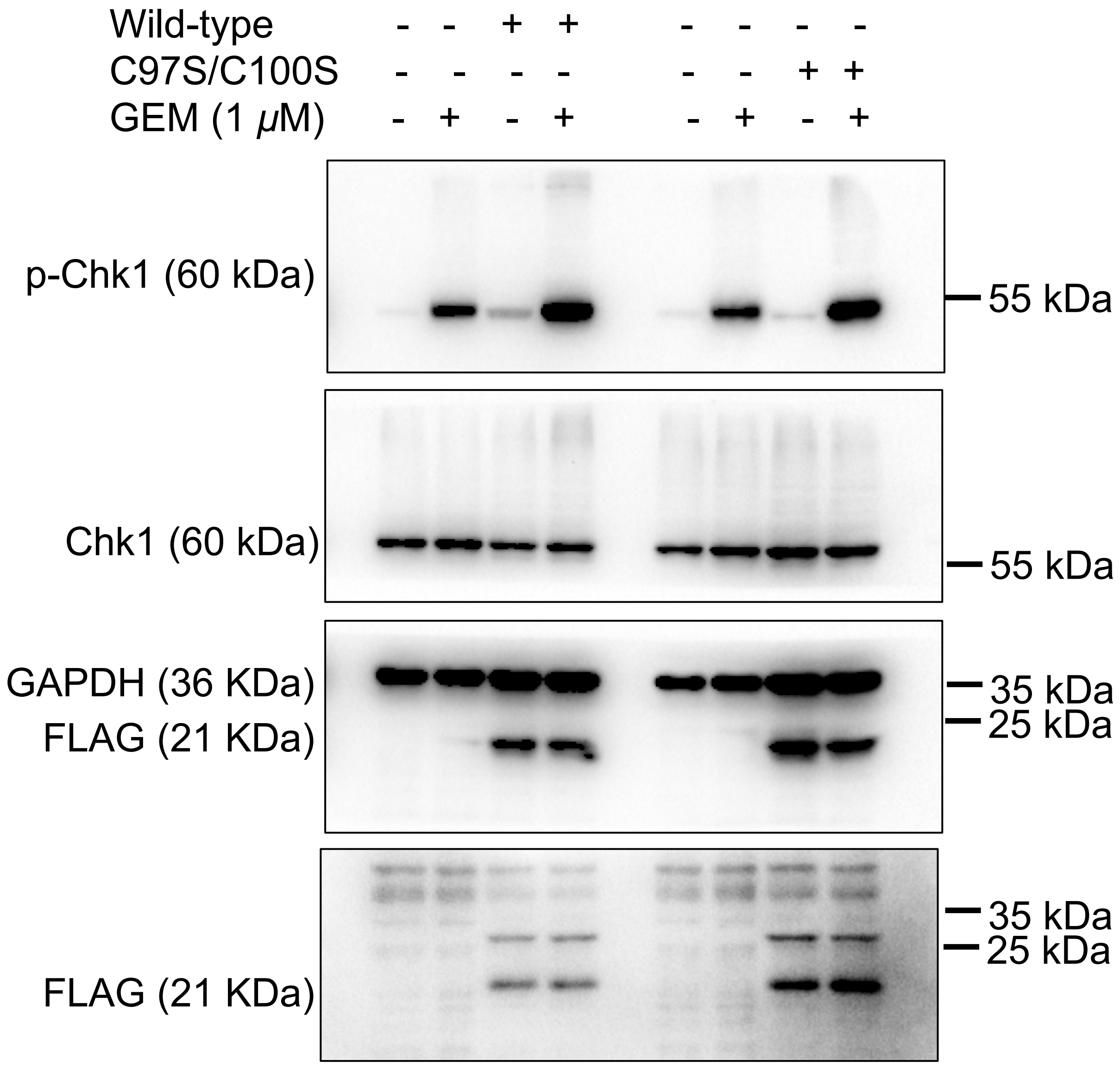
Wild-type - - + + - - - -
C97S/C100S - - - - - - + +
GEM (1 μM) - + - + - + - +
p-Chk1 (60 kDa)
55 kDa
Chk1 (60 kDa)
55 kDa
GAPDH (36 KDa)
FLAG (21 KDa)
35 kDa
25 kDa
FLAG (21 KDa)
35 kDa
25 kDa

Fig. 2H

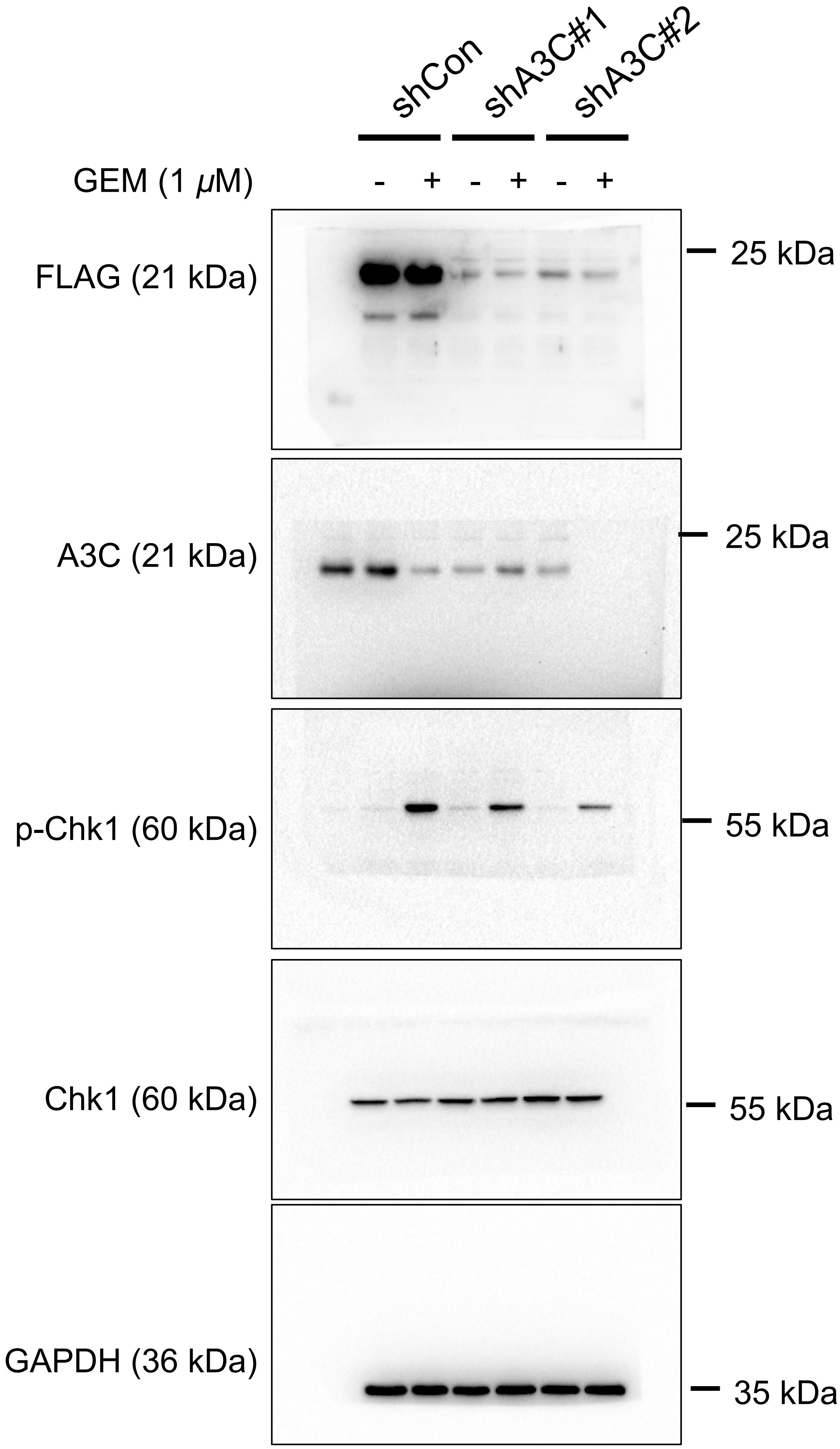
shCon
shA3C#1
shA3C#2
GEM (1 μM)
- + - + - +
FLAG (21 kDa)
25 kDa
A3C (21 kDa)
25 kDa
p-Chk1 (60 kDa)
55 kDa
Chk1 (60 kDa)
55 kDa
GAPDH (36 kDa)
35 kDa

Fig. 4D

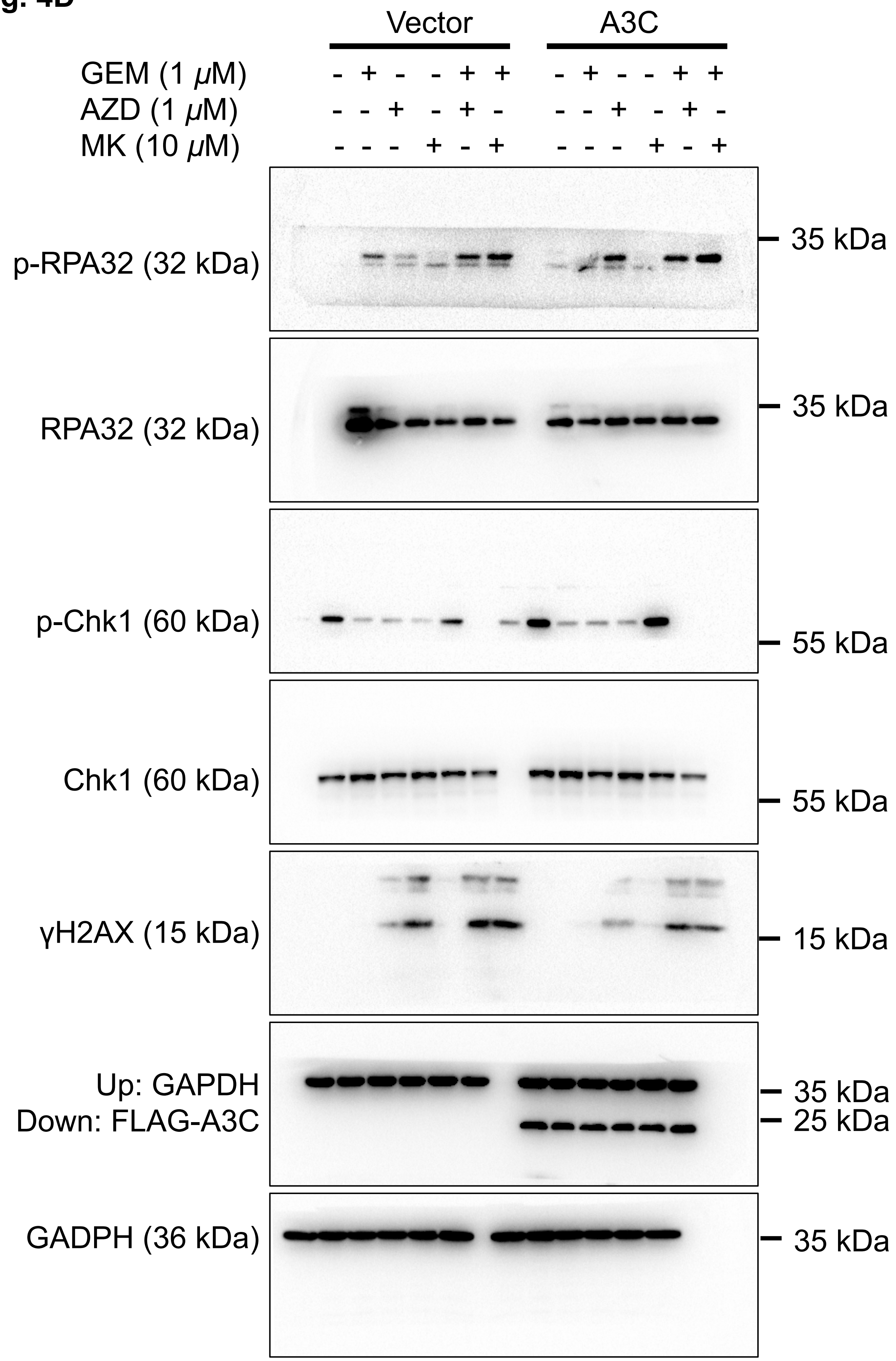
Vector
A3C
GEM (1 μM) - + - - + + - + - - + +
AZD (1 μM) - - + - + - - - + - + -
MK (10 μM) - - - + - + - - - + - +
p-RPA32 (32 kDa)
35 kDa
RPA32 (32 kDa)
35 kDa
p-Chk1 (60 kDa)
55 kDa
Chk1 (60 kDa)
55 kDa
γH2AX (15 kDa)
15 kDa
Up: GAPDH
Down: FLAG-A3C
35 kDa
25 kDa
GADPH (36 kDa)
35 kDa

Fig. 5F

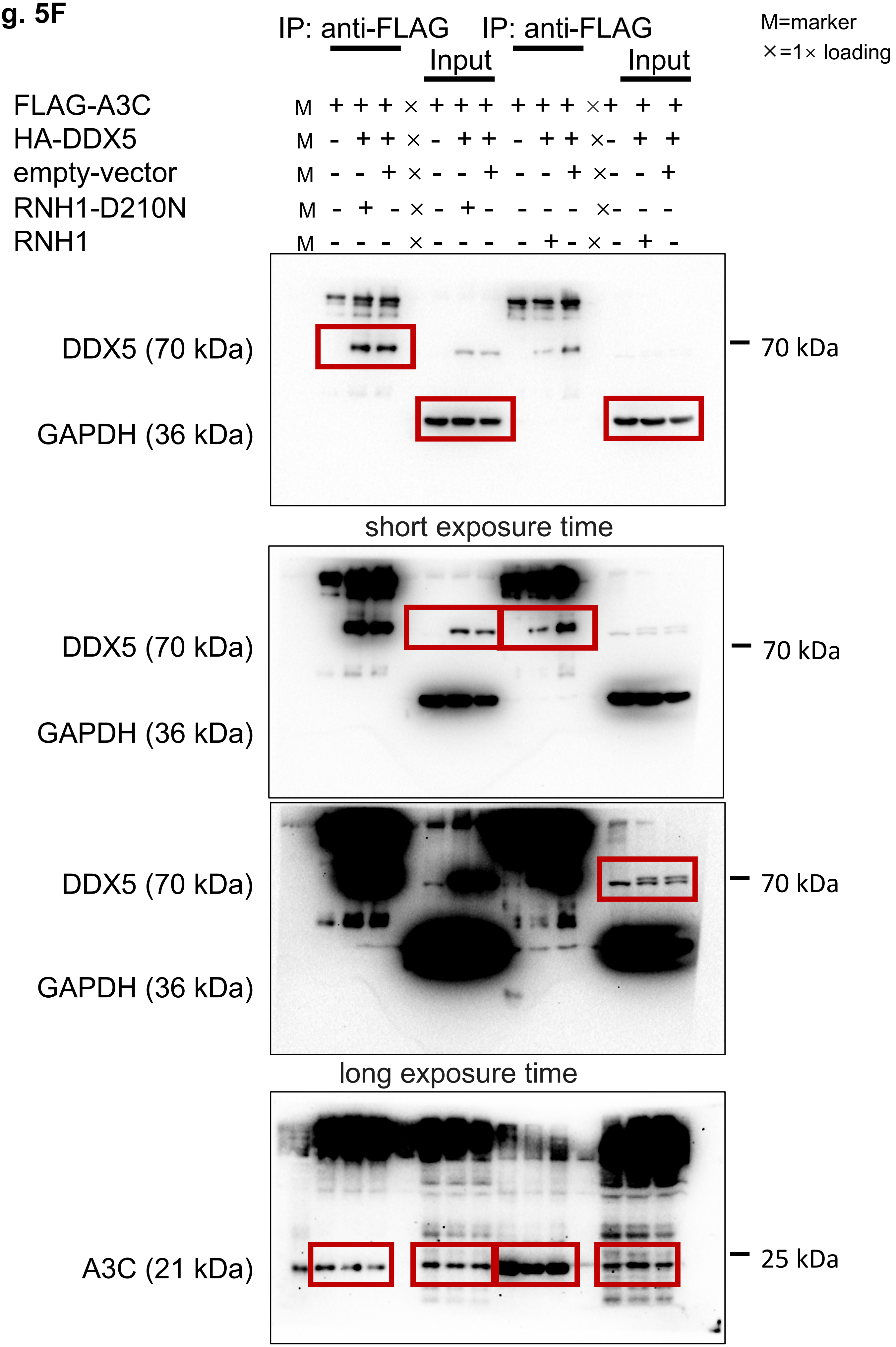
IP: anti-FLAG
Input
IP: anti-FLAG
Input
M=marker
×=1× loading
FLAG-A3C
HA-DDX5
empty-vector
RNH1-D210N
RNH1
DDX5 (70 kDa)
GAPDH (36 kDa)
70 kDa
short exposure time
DDX5 (70 kDa)
GAPDH (36 kDa)
70 kDa
DDX5 (70 kDa)
GAPDH (36 kDa)
70 kDa
long exposure time
A3C (21 kDa)
25 kDa

Fig. 5H

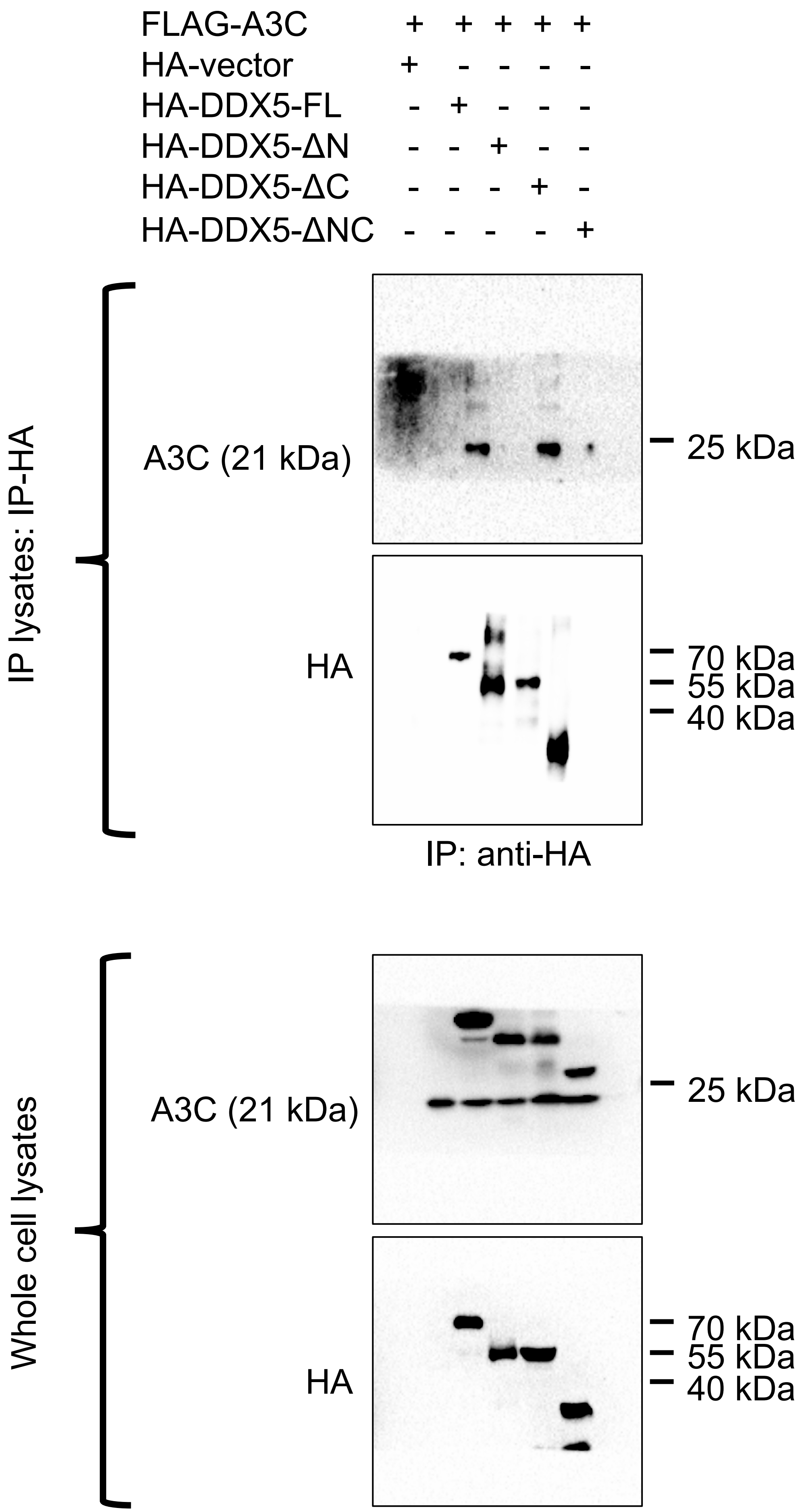

FLAG-A3C + + + + +
HA-vector + - - - -
HA-DDX5-FL - + - - -
HA-DDX5-ΔN - - + - -
HA-DDX5-ΔC - - - + -
HA-DDX5-ΔNC - - - - +
IP lysates: IP-HA
A3C (21 kDa)
25 kDa
HA
70 kDa
55 kDa
40 kDa
IP: anti-HA
Whole cell lysates
A3C (21 kDa)
25 kDa
HA
70 kDa
55 kDa
40 kDa

Fig. 6B

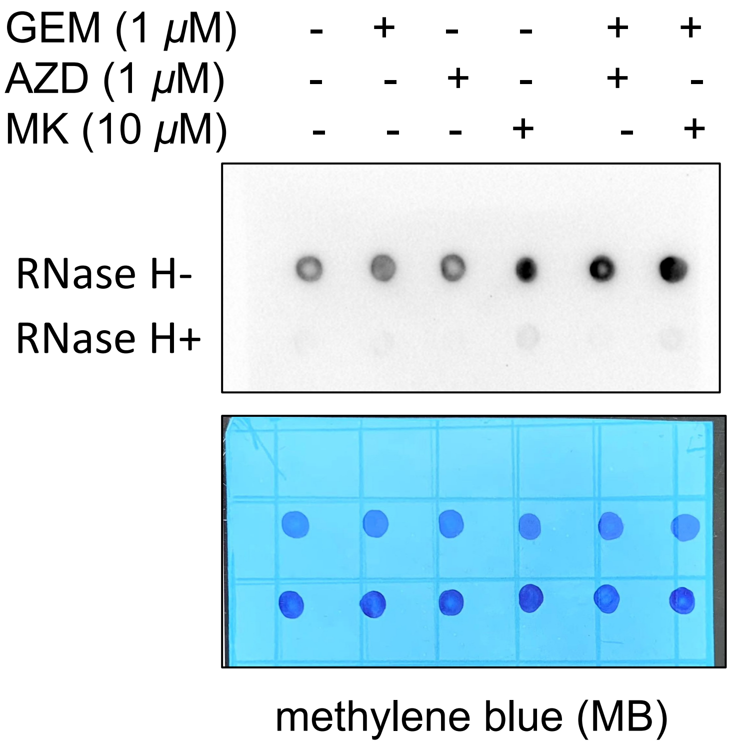
GEM (1 μM) - + - - + +
AZD (1 μM) - - + - + -
MK (10 μM) - - - + - +
RNase H-
RNase H+
methylene blue (MB)

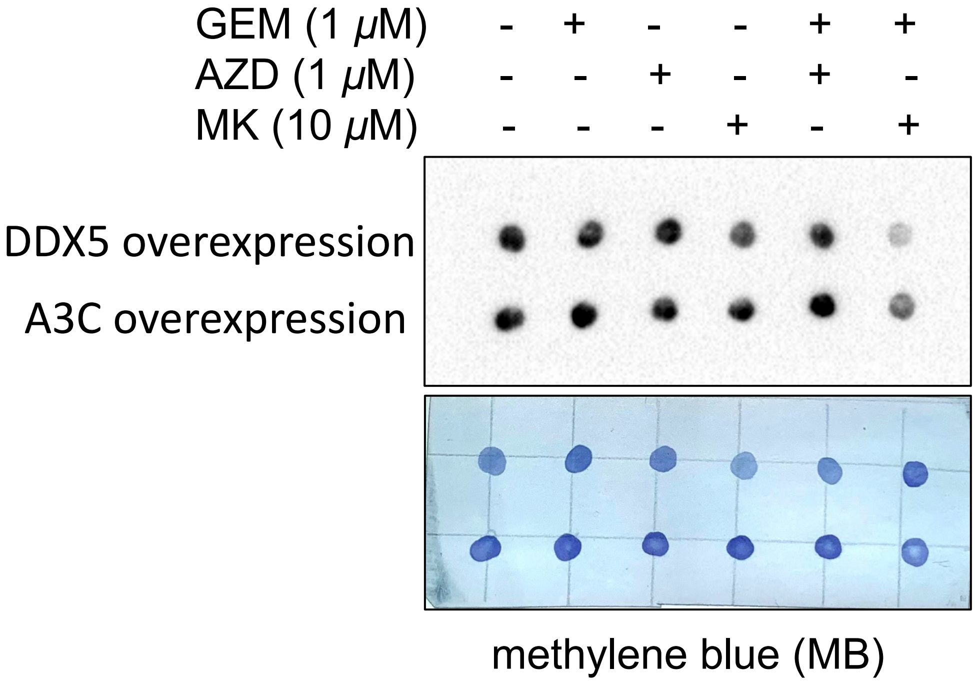
GEM (1 μM) - + - - + +
AZD (1 μM) - - + - + -
MK (10 μM) - - - + - +
DDX5 overexpression
A3C overexpression
methylene blue (MB)

Fig. 6D

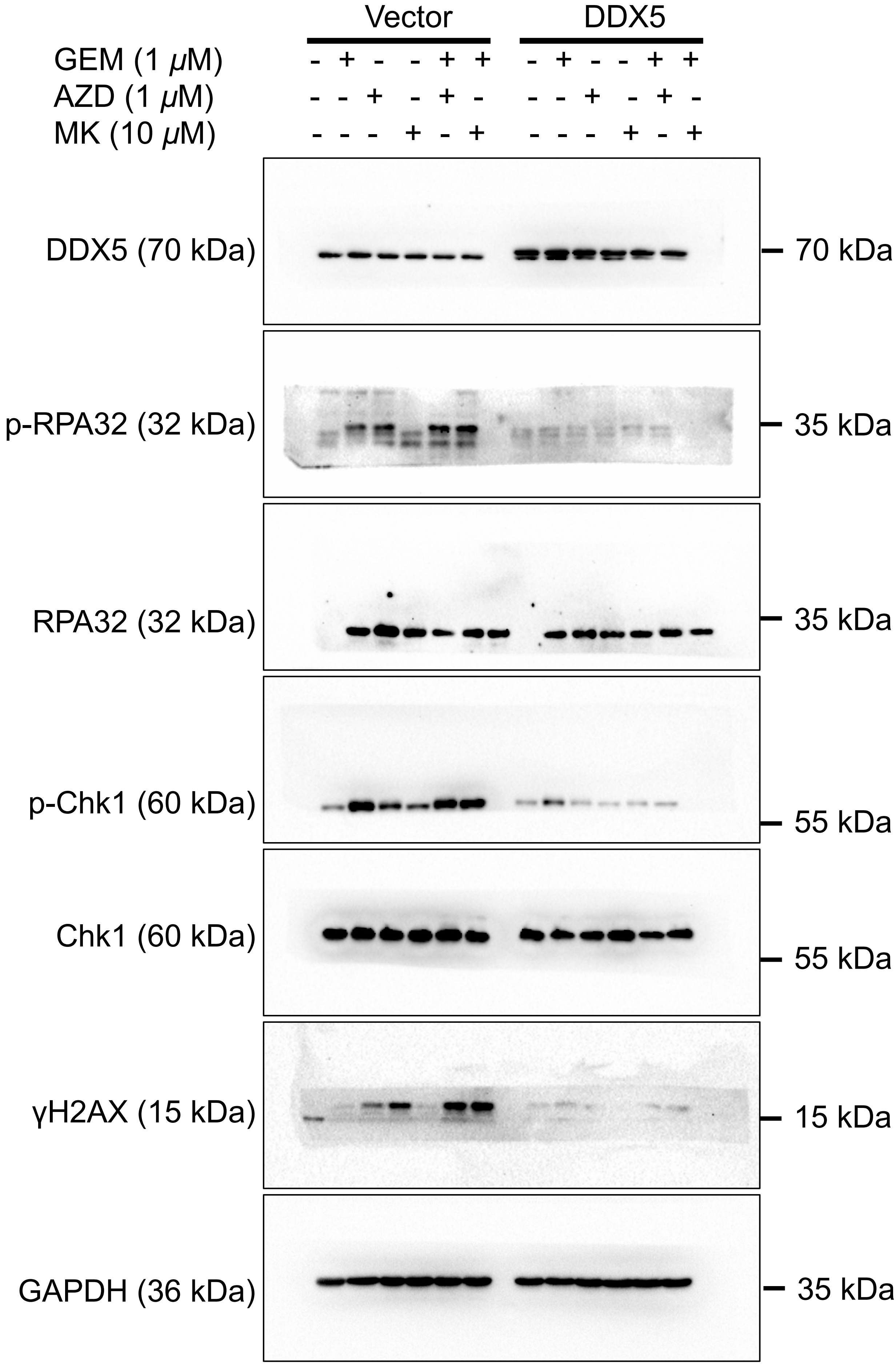

Vector
DDX5
GEM (1 μM) - + - - + + - + - - + +
AZD (1 μM) - - + - + - - - + - + -
MK (10 μM) - - - + - + - - - + - +
DDX5 (70 kDa)
70 kDa
p-RPA32 (32 kDa)
35 kDa
RPA32 (32 kDa)
35 kDa
p-Chk1 (60 kDa)
55 kDa
Chk1 (60 kDa)
55 kDa
γH2AX (15 kDa)
15 kDa
GAPDH (36 kDa)
35 kDa

Fig. 6E

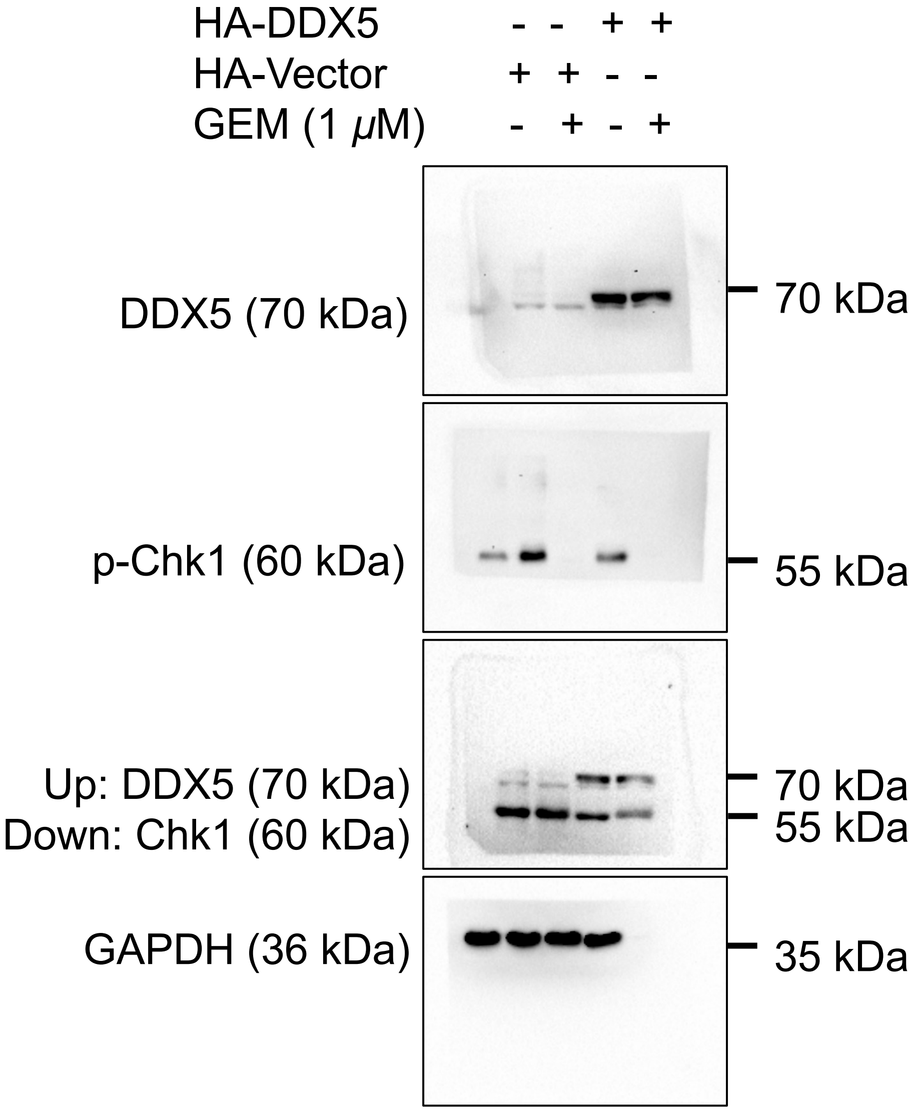

HA-DDX5 - - + +
HA-Vector + + - -
GEM (1 μM) - + - +
DDX5 (70 kDa)
70 kDa
p-Chk1 (60 kDa)
55 kDa
Up: DDX5 (70 kDa)
Down: Chk1 (60 kDa)
70 kDa
55 kDa
GAPDH (36 kDa)
35 kDa

Fig. 7A

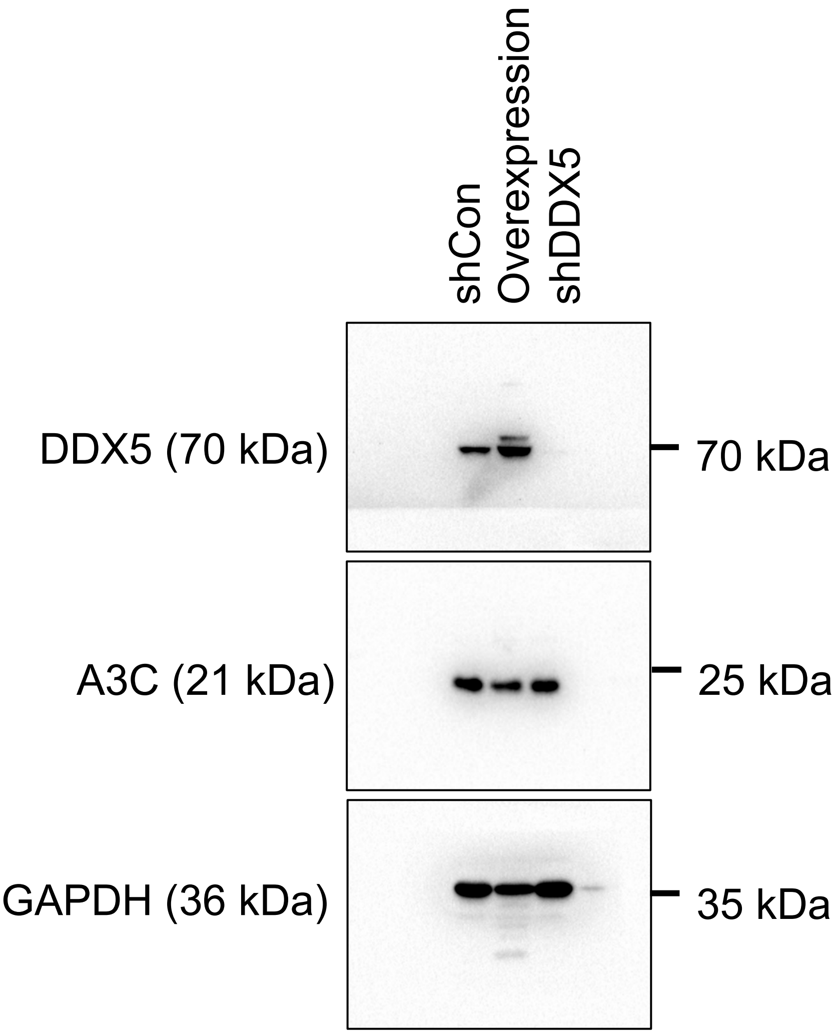
shCon
Overexpression
shDDX5
DDX5 (70 kDa)
70 kDa
A3C (21 kDa)
25 kDa
GAPDH (36 kDa)
35 kDa

Fig. 7B

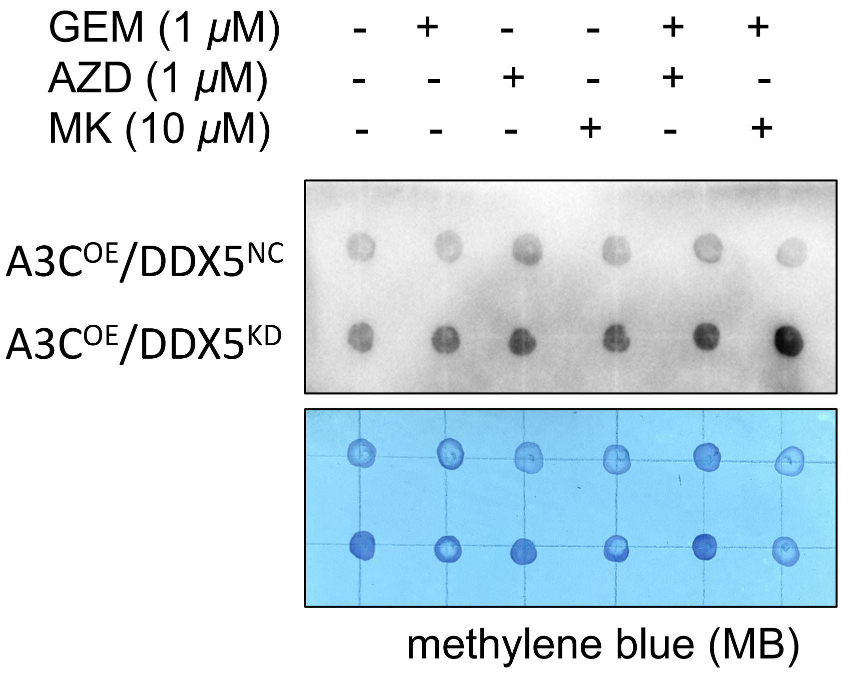

GEM (1 μM) - + - - + +
AZD (1 μM) - - + - + -
MK (10 μM) - - - + - +
A3COE/DDX5NC
A3COE/DDX5KD
methylene blue (MB)

Supplementary Fig. 2

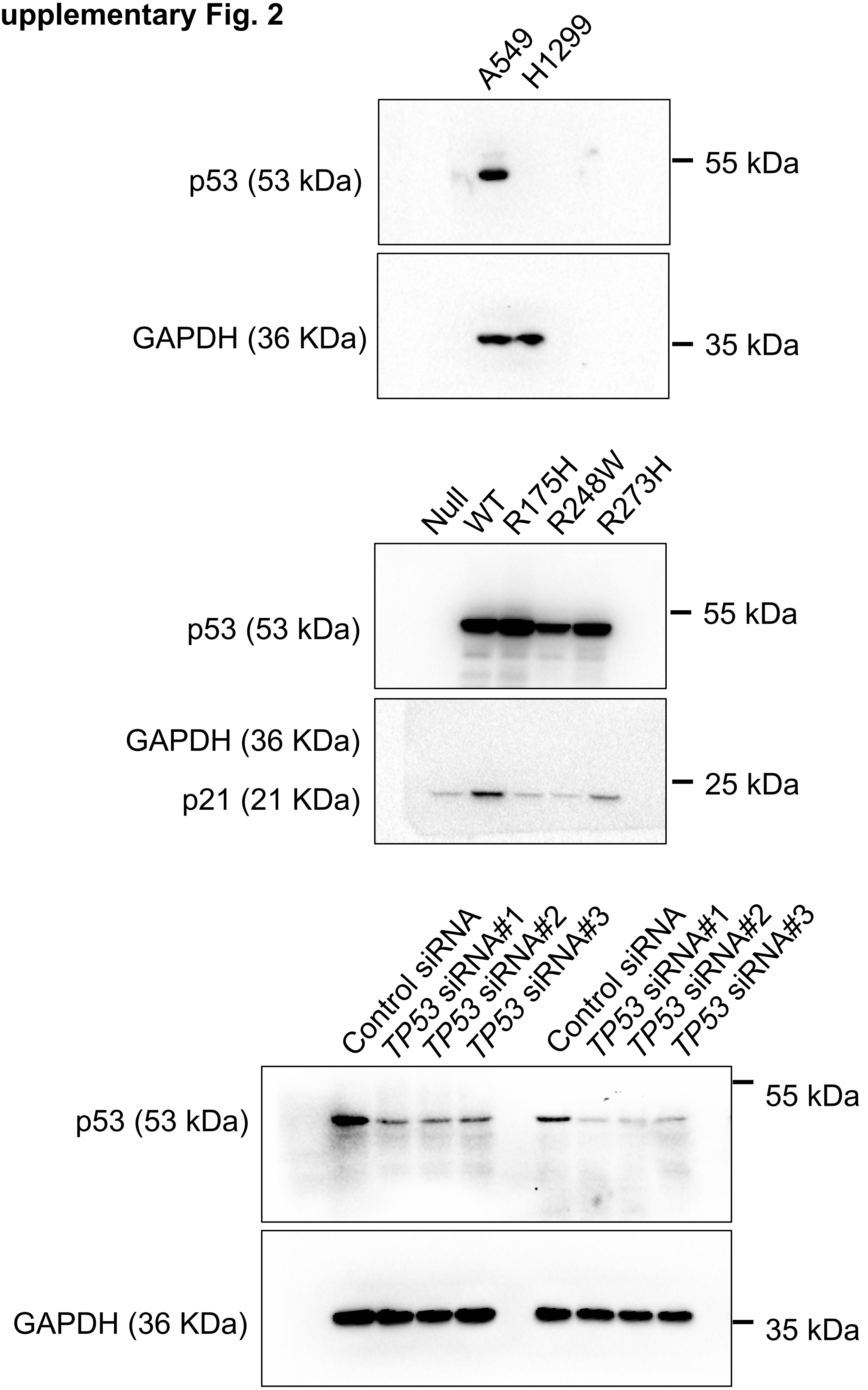

Supplement: Supplementary file 2 — Raw blots [file 41419_2025_8215_MOESM2_ESM.pdf]
